# Motif signatures in stretch enhancers are enriched for disease-associated genetic variants

**DOI:** 10.1186/s13072-015-0015-7

**Published:** 2015-07-16

**Authors:** Daniel X Quang, Michael R Erdos, Stephen C J Parker, Francis S Collins

**Affiliations:** Department of Computer Science, University of California, Irvine, Irvine, CA 92697 USA; Center for Complex Biological Systems, University of California, Irvine, Irvine, CA 92697 USA; National Human Genome Research Institute, National Institutes of Health, Bethesda, MD 20892 USA; Departments of Computational Medicine and Bioinformatics and Human Genetics, University of Michigan, Ann Arbor, MI 48109 USA

## Abstract

**Background:**

Stretch enhancers (SEs) are large chromatin-defined regulatory elements that are at least 3,000 base pairs (bps) long, in contrast to the median enhancer length of 800 bps. SEs tend to be cell-type specific, regulate cell-type specific gene expression, and are enriched in disease-associated genetic variants in disease-relevant cell types. Transcription factors (TFs) can bind to enhancers to modulate enhancer activity, and their sequence specificity can be represented by motifs. We hypothesize motifs can provide a biological context for how genetic variants contribute to disease.

**Results:**

We integrated chromatin state, gene expression, and chromatin accessibility [measured as DNase I Hypersensitive Sites (DHSs)] maps across nine different cell types. Motif enrichment analyses of chromatin-defined enhancer sequences identify several known cell-type specific “master” factors. Furthermore, de novo motif discovery not only recovers many of these motifs, but also identifies novel non-canonical motifs, providing additional insight into TF binding preferences. Across the length of SEs, motifs are most enriched in DHSs, though relative enrichment is also observed outside of DHSs. Interestingly, we show that single nucleotide polymorphisms associated with diseases or quantitative traits significantly overlap motif occurrences located in SEs, but outside of DHSs.

**Conclusions:**

These results reinforce the role of SEs in influencing risk for diseases and suggest an expanded regulatory functional role for motifs that occur outside highly accessible chromatin. Furthermore, the motif signatures generated here expand our understanding of the binding preference of well-characterized TFs.

**Electronic supplementary material:**

The online version of this article (doi:10.1186/s13072-015-0015-7) contains supplementary material, which is available to authorized users.

## Background

Chromatin immunoprecipitation combined with high-throughput sequencing (ChIP-seq) can identify the genome-wide locations of target proteins, including transcription factors (TFs), RNA Polymerase II, and covalently modified histones [1]. ChIP-seq datasets for several chromatin marks and the sequence-specific factor CTCF can be computationally integrated to discover combinatorial and spatial patterns that produce a consistent annotation of promoter, enhancer, insulator, transcribed, and repressed chromatin states. In a recent study, we profiled the chromatin states of several cell lines using ChromHMM [2], which revealed the presence of large gene control elements that we designated “stretch enhancers” (referred to as SEs in this paper) [3]. By our definition, SEs have a length of at least 3,000 DNA base pairs (bps) and are much larger than typical enhancers (TEs), which we defined to be any enhancer less than or equal to the median enhancer length of 800 bps. SEs are generally cell type specific, associated with increased cell-specific gene expression, and tend to harbor disease-relevant genetic variants derived from genome-wide association studies (GWASs). In our previous paper [3], we showed that enrichment for GWAS variants increases with the length of enhancers, but we did not try to define the precise relationship of GWAS variants to motifs located within the enhancers—that is the goal of this paper. SEs share several traits with another recently defined class of large enhancers designated super-enhancers by Young and colleagues [4, 5]. Like SEs, super-enhancers drive cell-type-specific gene expression; however, super-enhancers have been defined by the disproportionate abundance of Mediator or histone 3 lysine 27 acetylation (H3K27ac) signal [6], whereas SEs are defined by patterns of histone modifications, or chromatin states. Enhancers can function independently of their endogenous spatial contexts, which is a property exploited in luciferase assays to measure enhancer activity by placing enhancers upstream of reporter genes. This suggests that the information required for the enhancer activity is encoded in the underlying DNA sequences. We, therefore, hypothesize that the sequence content of the enhancers can provide additional insight into the relationship between enhancer function and enhancer length, which we address by studying how SEs differ from TEs.

Enhancer sequences are known to be enriched for transcription factor binding sites, which contribute to enhancer activity. Upon binding to their recognition motifs, some TF proteins can form complexes with other proteins, which can alter the 3D conformation of chromatin and recruit RNA Polymerase II to promote the transcription of target genes located in *cis*, sometimes at considerable distances. The motif, or sequence binding specificity, for a TF can be represented as a position weight matrix (PWM) that specifies the nucleotide frequency at each position along the binding sequence. Recently, less complex nucleotide patterns—like dinucleotide repeats—were shown to contribute to enhancer activity [7].

In this study, we analyze the motif signatures of SEs and how they differ from those of TEs. We scan enhancer sequences using known motifs from databases to identify TFs that are characteristic of each cell type studied, which we are also able to recover using de novo motif discovery. We investigate how GWAS single nucleotide polymorphisms (SNPs) can affect TF motif signatures in SEs, which can provide important clues about how genetic variations contribute to disease risk.

## Results and discussion

### Systematic chromatin state, DNase hypersensitivity, and gene expression profiling across nine diverse cell types

In our previous study, we used the ChromHMM algorithm to systematically integrate ChIP-seq histone modification and CTCF datasets and uniformly profile chromatin states across ten diverse cell types. These ChromHMM segmentations are used to profile SEs, which are defined as regions of at least 3,000 bps containing contiguous segments marked as enhancer states. TEs are defined similarly, but are less than or equal to 800 bps in length. Although SEs only constitute the top 10% of enhancers in terms of length, they represent a disproportionately large percentage of the total number of nucleotides among all enhancers (Additional file 1: Table S1).

Of the ten cell types profiled, nine also have DNase-seq data available. DNase-seq is a method used to identify the genome-wide locations of DHSs, which are regions of the genome that are highly sensitive to cleavage by DNase I and mark regulatory elements such as enhancers and promoters [8]. DHSs generally mark regions that are more accessible for TF binding and are enriched for TF binding motifs.

**Figure 1:**
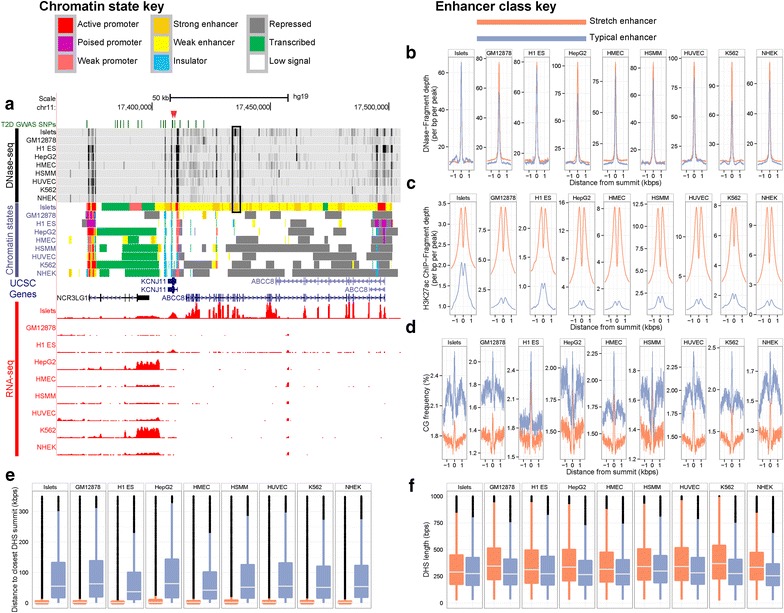
Systematic profiling of DNase I hypersensitivity, chromatin states, and gene expression across nine cell types. **a** Chromatin states and DNase I hypersensitivity density tracks in and around the ABCC8 locus. Human pancreatic islet chromatin states are similar to some of the other ENCODE cell types at the commonly expressed flanking gene NCR3LG1 and unique at the islet-specific expressed gene ABCC8. (*Upper*) DNase I Hypersensitivity Density Signal from ENCODE/Duke in dense format for each of nine human cell types (islets; GM12878, lymphoblastoid cells; H1 ES, embryonic stem cells; HepG2, hepatocellular carcinoma; HMEC, mammary epithelial cells; HSMM, smooth muscle myoblasts; HUVEC, umbilical vein endothelial cells, K562, erythroleukemia cells, NHEK, keratinocytes). Density graphs (wiggles) of signal enrichment calculated using F-Seq are displayed in grayscale. *Scale* is from 0 to 0.1. (*Middle*) ChromHMM-defined chromatin states. Chromatin state assignments are indicated in the *top-leftmost* key. (*Lower*) RNA-seq-based expression for each cell type is measured in reads per million mapped reads (RPM) per base pair. *Scale* is from 0 to 2 for each cell type. Index and tightly linked (r^2^ ≥ 0.8) SNPs associated with T2D are indicated in *green* in the T2D GWAS SNPs track and primarily reside in islet-specific SEs. Index SNPs rs5215 and rs5219 are marked with *red arrows*. The *black box* highlights a portion of the SE previously shown to direct tissue-specific expression patterns in a spatial and temporal manner in vivo [3]. All processed results are browsable and downloadable at http://research.nhgri.nih.gov/manuscripts/Collins/isletchromatin/. **b**–**d** Aggregate DNase-seq tag density (**b**), H3K27ac ChIP-seq tag density, (**c**), and (**d**) CG dinucleotide frequency profiles of 3kbp sequences centered on DNase-seq peak summits—the location of the highest DNase-seq signal—located within SEs or TEs. **e** DHSs within SEs are much closer together than they are within TEs (two-tailed Wilcoxon rank sum test, p < 10^−100^ for all cell types). *Boxplots* show the distance, for each SE or TE DNase-seq summit, to the nearest SE or TE DNase-seq summit, respectively, for all cell types. **f** DHSs within SEs are moderately longer than DHSs within TEs (two-tailed Wilcoxon rank sum test, p < 10^−5^ for all cell types). Boxplots show the length of each DHS whose summit overlaps a SE or TE. *Boxplo*t whiskers extend to 1.5× the interquartile range and outliers are shown as *block dots*, but the y-axis is truncated so that the *boxplots* can remain in view. Enhancer classes for **b**–**f** are indicated in the *top-rightmost* key.

Figure 1a displays the accessible chromatin, chromatin state, and gene expression profiles across the nine cell types around the *ABCC8* locus. Gene expression RNA-seq [9] tracks clearly show that the *ABCC8* transcript is exclusively expressed in the islet sample, and the chromatin state tracks show this gene body is covered by several islet-specific SEs. This integrative approach can identify cell-specific chromatin and expression profiles to provide a basis for further understanding the functional effects of SNPs in common, complex diseases. In the *ABCC8* example, lead type 2 diabetes (T2D) GWAS SNPs (red arrow heads) and several linked SNPs (r^2^ ≥ 0.8) (green bars) overlap islet-specific chromatin states. Enhancers have been shown to overlap multiple linked SNPs more often than expected at random [3, 10], suggesting that multiple enhancer variants work together in concert to alter gene expression and contribute to disease susceptibility.

From the *ABCC8* example, we can infer some properties about the interplay between chromatin states and DNase I hypersensitivity in SEs. First, we notice that the SEs encompass several DHSs, but the entire SE does not display DNase hypersensitivity. Unsurprisingly due to their length, SEs contain proportionately more DHSs than TEs do (Additional file 2: Figure S1). Second, strong enhancer states in islets in ABCC8, for example (Figure 1a), tend to overlap spikes in the islet DNase-seq signal. By aggregating the DNase-seq tag density relative to DNase-seq peak summits in either SEs or TEs, we find there is relatively little difference in DNase I hypersensitivity between the classes of enhancers (Figure 1b). However, there is a large difference in the H3K27ac ChIP-seq signal (Figure 1c). Notably, there is a dip in the H3K27ac signal in the center of the DNase peak summit, which is a reflection of bound TFs that can displace histones. This TF-induced configuration of chromatin architecture is much more pronounced in SEs than it is in TEs. Of relevance, this H3K27ac dip feature recently provided the central motivation for a novel computational algorithm to detect factor binding sites [11]. Given the pronounced dip in SEs versus TEs, this recent computational algorithm likely picked up mostly on SE-mediated factor binding sites. In generating the signal histogram plots, we accounted for the minor difference in mappability between SEs and TEs. Generally, SEs are slightly more mappable than TEs (Additional file 2: Figure S2), which suggests that SE sequences are less repetitive and more complex than TEs. Such sequence composition differences could be characteristic of the two enhancer classes. Nevertheless, even after accounting for differences in mappability, there is still a large statistical difference in H3K27ac signal across the length of the enhancers (Additional file 2: Figure S3).

To examine functional and sequence composition differences between the two enhancer classes more comprehensively, we plotted the average Genomic Evolutionary Rate Profiling (GERP) score [12] (Additional file 2: Figure S4), a metric that estimates position-specific evolutionary constraint across a multi-species sequence alignment, and dinucleotide frequencies (Figure 1d, Additional file 2: Figure S5) at each position relative to DNase-seq peak summits in either SEs or TEs. Generally, SEs and TEs show similar patterns of evolutionary constraint around DNase-seq peak summits. GERP scores are highest at the summit and decrease as one moves away from the summit. At the summits, SEs have a slightly higher average GERP score than TEs. SE and TE sequences are particularly different in terms of their CG dinucleotide frequencies: both SEs and TEs display a large spike in CG frequency in the DHS summits, but TE sequences are much more CG-rich and overlap CpG islands more often (Additional file 2: Figure S6). Notably, TFs tend to bind to CG-rich DNase I accessible regions, but the CG richness of some motifs do not account for this spike in CG frequency [13]. The variation in dinucleotide frequencies is largely a function of where these enhancers are located in the genome relative to gene models (Additional file 2: Figure S7). When restricted to transcription start site (TSS) distal regions, the dinucleotide differences are mitigated, but the difference in H3K27ac ChIP-seq signal persists (Additional file 2: Figure S8). In some of these histogram plots, we note that the differences can persist several hundred base pairs away from the center. One possible reason for this phenomenon is the proximity between DHSs in SEs. Although SEs are much longer than TEs, DHSs in SEs are still spaced comparatively close together (Figure 1e). DHSs in SEs are also significantly longer than DHSs in TEs (Figure 1f), which suggests that individual DHSs in SEs can accommodate more TF binding sites than DHSs in TEs can.

### Enhancer sequences are enriched for known motifs

Different cell types are regulated by sets of TFs that are important for establishing and maintaining cell identity. Enhancers are expected to be enriched for motifs that serve as putative TF binding sites. We hypothesize that DHSs in enhancers should be especially enriched for motif sites, because these regions are more accessible for protein–DNA interactions. In particular, enhancers would be expected to be bound by a specific class of TFs called activators, which increase gene transcription.

**Figure 2:**
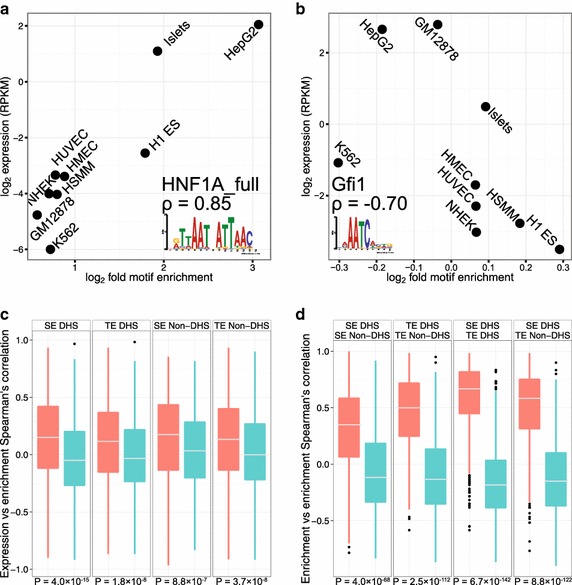
Motif enrichments in enhancers are correlated with the expression of the TFs that bind to these motifs. **a**, **b** TFs’ expressions are correlated against the enrichment of their respective binding motifs in SE DHS sequences across the nine cell types. The master activator HNF1A (**a**) has a positive relationship between gene expression and motif enrichment, while the repressor GFI1 (**b**) has a negative relationship. The name of the motif, Spearman’s correlation (ρ), and sequence logo of the motif are displayed in the corner of the plots. **c** *Boxplots* of Spearman’s correlations of enrichments in four different regions for all database motifs against gene expression in the nine cell types. **d** Boxplots of Spearman’s correlations of motif enrichments between two different regions (listed in the strip titles at the *top* of the facets). For each motif and each pair of regions, we computed the enrichments of the motif in both sets of sequences and then computed the Spearman’s correlation between the two sets of enrichments. Within each facet, the *boxplot* of Spearman’s correlations (*red*, *left*) is also displayed alongside a *boxplot* of a null distribution (*blue*, *right*) generated by recalculating the Spearman’s correlations after shuffling cell assignments for one of the variables. P-values below the *boxplots* represent the significance of the distribution compared to the respective null distributions and are calculated with the Wilcoxon rank sum test. Gene expression is measured in reads per kilobase per million (RPKM). Motif sites were identified with FIMO, a tool for searching occurrences of known motifs in biological sequences [34]. Motif enrichment is calculated as the ratio of FIMO hits in the positive sequence set to FIMO hits in dinucleotide shuffled negative control (“Methods”).

To identify activator motifs, we searched for a relationship between motif enrichment and gene expression, similar in nature to a previous approach [2], and demonstrated that the gene expression of activators (by mRNA quantification) correlates positively with the enrichment of its binding motif in SE DHS sequences across the nine cell types. For instance, HNF1A, an activator highly expressed in the liver, shows a very strong positive relationship between its expression across the nine cell types and the enrichment of its binding motif in SE DHS sequences (Figure 2a). In contrast, repressors such as GFI1 tend to have a negative correlation (Figure 2b). These observations reflect the general concept of TF binding cooperativity on the DNA scaffold whereby increases in the number of binding sites results in increased enhancer activity [14, 15]. We hypothesize that enhancer sequences are organized in a way such that an increase in expression of the relevant activator is accommodated by an increase in available binding sites, while reducing potential binding sites for any present repressors. To explore this idea more comprehensively, we studied the distribution of motif enrichment versus gene expression correlations across motif-TF pairs for SE DHS sequences, as well as SE non-DHS, TE DHS, and TE non-DHS sequences (Figure 2c). All four observed distributions (red) display a significant positive correlation bias relative to the null expectation (blue), indicating that SEs and TEs have well-organized motif architecture both inside and outside of DHSs. To determine whether the relative motif enrichments are the same across different enhancer regions, we examine the correlation between a motif’s enrichment in one genomic region against its enrichment in a different genomic region (e.g. CTCF motif enrichment in SE DHS sequences across cell types versus its enrichment in TE DHS sequences across cell types). We then measure the distribution of correlations between enrichments for all motifs in different pairs of regions (Figure 2d). Despite the difference in absolute motif enrichments in different enhancer regions, we find that the relative motif enrichments are strongly preserved. Together, these findings indicate that enhancer motif architecture is linked to TF gene expression and preserved within and outside DHSs in SEs and TEs.

**Figure 3:**
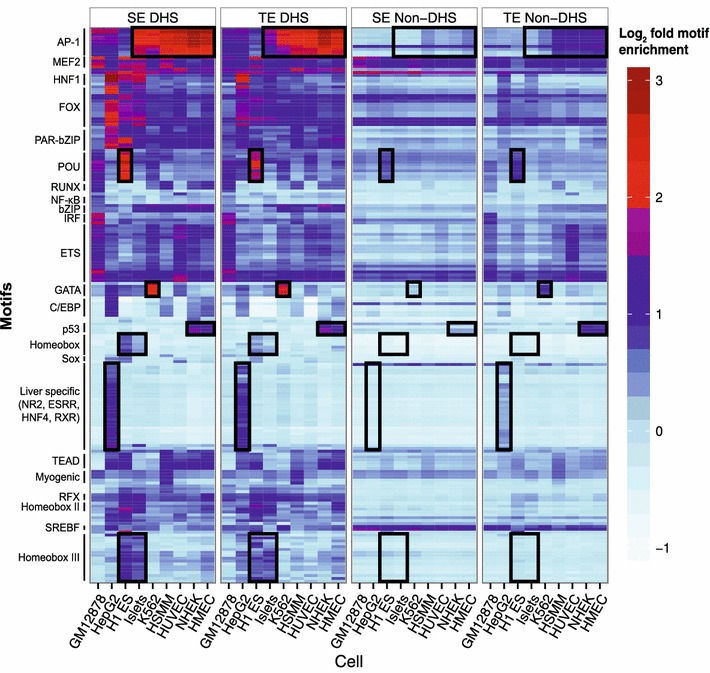
Heatmaps of enrichment of the binding motifs of activator TFs across nine cell types and four regions. Shading indicates log2 enrichment of motifs in sequences of the specified cell and region. (Clustering was done using SE DHS motifs). To improve visualization of the heatmaps, the original set of motifs is pruned through a strategy that includes removing any motifs for which the corresponding TF fails to be significantly expressed in any of the nine cell types. This pruning strategy reduces the motif set to primarily include motifs of activator and master factors. The remaining motifs are clustered and ordered by exemplar-based agglomerative clustering on the log2 enrichment values across the nine cell types (“Methods”). Groups or families of motifs are manually labeled on the *left side*. *Black boxes* highlight cell-specific motif enrichment clusters.

Next, we investigate how motif enrichments vary across different motifs, cell types, and enhancer regions. We perform agglomerative clustering on the log_2_ fold enrichment in SE DHS sequences of activator motifs that are significantly and differentially enriched in enhancer sequences, in order to group motifs in an unsupervised fashion (“Methods”). This analysis results in clusters of motifs that are grouped corresponding to TF families known to play important roles in the cell types considered, which we visualize with heatmaps of motif enrichment across four different enhancer categories (Figure 3, Additional file 2: Figure S9). The heatmaps highlight the relative motif enrichments in enhancers between and within the cell types, capturing known biologically relevant cell-TF associations. For example, the GATA cluster is K562-specific, which is appropriate because K562 is an erythroleukemia cell line, so we expect the motif of the erythroid fate determining TF GATA-1 [16] to be over-represented in K562 enhancers. As expected, motifs are most enriched in DHS sequences. Non-DHS regions in enhancers are also enriched for some motifs, but to a much lesser extent than their DHS counterparts. Despite how clustering was performed on enrichment values focused on the SE DHS sequences, many of the enrichment clusterings in the other enhancer regions are preserved (Figure 3, black boxes), further supporting the concept of common motif architecture across the different enhancer regions.

**Figure 4:**
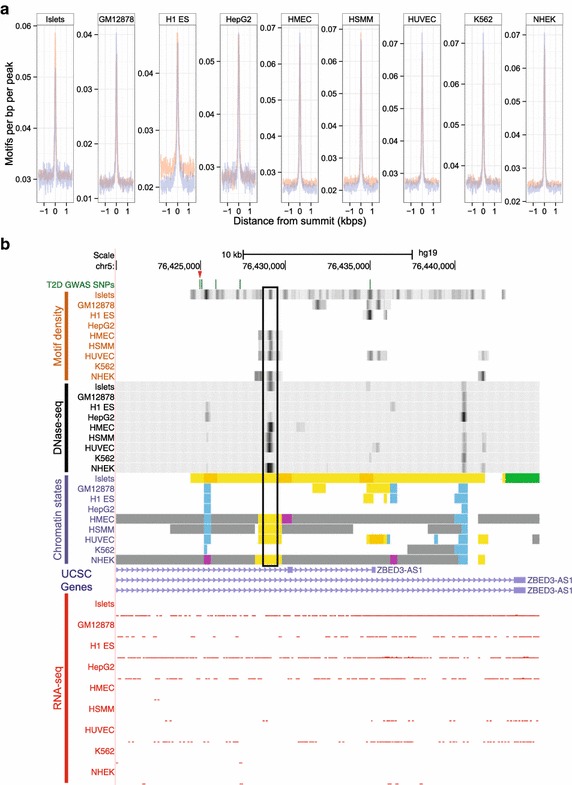
Motifs are most enriched around the summits of DNase-seq peaks. **a** Aggregate motif density plots of 3kbp sequences centered on DNase-seq peak summits within SEs or TEs. *Line plots* are *colored* as in the enhancer class key in Figure 1. Cell-specific motif sets are generated by selecting motifs that are significantly enriched in SE DHS sequences and correspond to TFs that are significantly expressed. Individual motif density plots are generated by the Homer annotate Peaks tool [39] with motifs called at a threshold of 40% of the maximum log likelihood score. These individual motif density plots are summed together position-wise to generate the aggregate motif density plots (“Methods”). Motif densities are generally similar between the SE and TE sequences. **b** UCSC Genome browser view of the ZBED3-AS1 locus. Motif density tracks (*Upper*) identify portions of the genome assigned an enhancer state that are relatively rich in motifs. These tracks measure the number of non-overlapping motif sites in 150 bp windows at 10 bp steps and are auto-scaled for each cell type. Potentially functional SNPs that overlap SEs may not overlap any DHS, but may overlap portions of the SEs that are dense in motifs, such as the rightmost T2D tightly linked SNP. Chromatin state colors and scales for the other tracks are assigned as in Figure 1a. The *black box* highlights a strong overlap of cell-specific accessible chromatin and enhancer chromatin states that is also dense in motifs. These tracks are downloadable at http://fusion.nhgri.nih.gov/files/se-motifs/motifsBedgraphs.tar.

We next investigate how motifs are spatially distributed in SEs and TEs. For each cell type, we generated a set of activator motifs that are highly enriched in the respective SE DHS sequences and computed the density histograms of motif occurrences in SEs or TEs relative to the summits of DNase-seq peaks. Motif density is proportional to DNase I hypersensitivity, being greatest at the summits of DNase I hypersensitivity peaks and decreasing away from the summits (Figure 4a, Additional file 2: Figure S10). Based on these aggregate plots and heatmaps, SEs and TEs display similar motif enrichment and density patterns in their DHSs. However, SEs represent a disproportionately large fraction of enhancers by nucleotide count (Additional file 1: Table S1). Furthermore, SEs contain more DHSs per enhancer (Additional file 2: Figure S1). Therefore, SEs are further set apart from shorter enhancers by a proportional increase in putative binding sites, but not by differences in motif density or enrichment within or close by to individual DHSs.

To examine how motifs are distributed across individual enhancers instead of aggregating across groups of enhancers, we generated tracks that display motif densities in enhancers (Figure 4b, Additional file 2: Figure S11) for motifs that are significantly and differentially enriched in enhancer sequences of a given cell type, which we refer to as cell identity motifs. Again, we observe that cell identity motifs cluster in DHSs, but we also find that these motif clusters often appear outside of DHSs. In this particular example, none of the linked T2D SNPs overlapped any DHSs, but they did overlap portions of the islet-specific SE that is relatively dense in islet cell identity activator motifs. Interestingly, the RNA-seq tracks show a lack of expression of the surrounding *ZBED3*-*AS1* gene, implying that the SE is likely acting on a distant gene. The lack of DNase hypersensitivity does not necessarily exclude the possibility that TFs are binding to these parts of SEs, since the specific chromatin marks extending across an SE suggest that the entire region is considerably more open than the average genomic segment. One possibility is that DNase-seq may not be able to identify chromatin accessibility in these particular locations. For example, in a comparative study with FAIRE-seq, another assay for mapping open chromatin sites, some open chromatin sites are unique to either DNase-seq or FAIRE-seq. Multiple lines of evidence suggest that these DNase-only and FAIRE-only sites correspond to real chromatin features [17]. It is also of note that some TFs are able to localize partially or predominantly within inaccessible chromatin [18].

### De novo motif discovery in enhancers

Computing enrichment of known motifs in enhancer sequences can provide insight into the motif landscape of these sequences, but it does not necessarily identify the actual set of TFs that bind to the enhancers. This is because different TFs can bind to very similar motifs, leading to ambiguity. Moreover, motif scanning analysis is limited to motifs that are available in databases, which are incomplete. Even for the TFs that are represented, databases often neglect infrequent non-canonical motifs. For example, one study showed that the protein Neuron Restrictive Silencer Factor can have, in addition to its prominent canonical motif, nine infrequent non-canonical motifs that are not present in any motif database [19]. In fact, motifs in databases may not accurately represent the in vivo binding context of TFs. For example, many of the database motifs we consider in this study were generated by high-throughput SELEX on single TFs [20], an in vitro assay that can miss important in vivo binding contexts. Additionally, DNA shape-based readout by TFs is not well captured by traditional PWMs [21] and can, therefore, result in missed target sites. Finally, results from a recent study show that disease-associated non-coding SNPs are not well captured by PWMs and may instead be better explained by non-conical sequence determinants [22].

**Figure 5:**
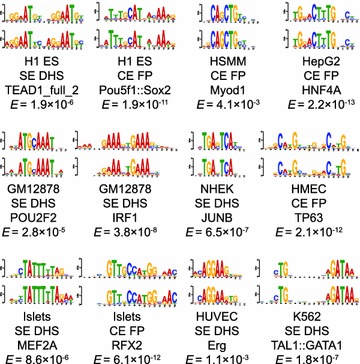
De novo motif discovery accurately recovers known motifs. Examples of sequence motifs that are enriched in Stretch Enhancer (SE) DNase Hypersensitive Site (DHS) sequences or within DNase footprints (FP) across all ChromHMM concatenated enhancers (CEs). Sequences of motifs derived from EXTREME (*bottom*) are aligned to known motifs from two databases [20, 37] (*top*). Motif similarity was measured with TOMTOM, a tool that reports the significance of a match between a query motif and a database motif [43]. Below each sequence logo, the cell type, sequence set, motif name, and significance of the TOMTOM match (*E*_*T*_) are displayed.

To further explore the motif landscape of enhancers, we apply de novo motif discovery on DHS sequences in SEs, which we showed are highly enriched for cell specific regulatory motifs. We also apply de novo motif discovery to DNase I “footprints” within all enhancers. Footprints are local dips in the DNase I cleavage signal, which are predicted to demarcate TF occupancy because TFs protect accessible chromatin from DNase I cleavage [23–25]. Therefore, footprint sequences should be much more enriched for motifs than the larger DHS sequences and can significantly improve the quality of de novo motif discovery. Due to the large number of nucleotides in the sequences we scan, we use EXTREME [19], a fast motif discovery algorithm designed for large sequence datasets. As a demonstration of the effectiveness of this approach, EXTREME is able to recover motifs that significantly match known signatures (Figure 5, Additional file 2: Figure S12). These motifs often correspond to TFs known to be associated with cellular differentiation and reprogramming (Additional file 3).

**Figure 6:**
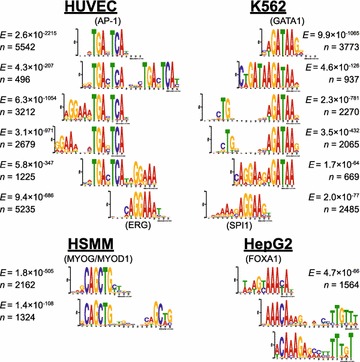
De novo motif discovery in enhancer footprint sequences reveals novel binding patterns of well-characterized TFs. Motifs of known activators in the HUVEC, K562, HSMM, and HepG2 cell lines can co-occur together. For example, in the HUVEC enhancer footprint sequences, the ERG motif, a member of the ETS family that is characterized by a “GGAA” binding paper, often co-occurs with the AP-1 motif. In the presence of the AP-1 motif, the degree of resemblance of a predicted site to the ERG motif is weaker. SPI1, another member of the ETS family, shares a similar relationship with the GATA1 motif in K562. In other examples, activator TFs appear to often homodimerize and form palindromic motifs. Sequence logos of examples of de novo motifs in the cell types are displayed alongside, if available, CentriMo E-values and number of matches in SE DHS sequences (“Methods”). For two of the HepG2 examples, the motifs are so infrequent that CentriMo failed to find a significant number of matches in SE sequences.

The de novo motif discovery analysis does not yield any prominent examples of novel motif families, possibly because the systems we consider have already been studied extensively. However, we do find that de novo motif discovery can provide novel insight into the spatial arrangement of motif combinations at nucleotide resolution. Some of the motifs discovered in footprint sequences appear as combinations of two known motifs in close spatial proximity (Figure 6). In HUVEC enhancers, for example, we find a significant number of activator protein 1 (AP-1) and ERG motif matches. AP-1 is a heterodimeric protein that recognizes and binds to the enhancer heptamer motif 5′-TGA[CG]TCA-3′. ERG is a subfamily of the ETS family of TFs, which have a strong 5′-GGAA-3′ core binding sequence within their binding motifs. The ERG subfamily includes TFs such as ERG and FLI1, which are known to be functionally active in HUVEC. Through our de novo motif analysis, we find these two classes of motifs are significantly co-enriched, but the frequency of the combination depends on the relative orientation of these two motifs. Furthermore, sequence-specific constraints for the ERG binding motif are relaxed when an AP-1 motif is nearby. These results suggest a motif regulatory “grammar” governed by physical constraints that dictate the in vivo spatial arrangements and frequencies of combinations of motifs, which is consistent with a previous report [26], and may uncover some of the non-canonical sequence determinants that underly disease-associated SNPs. Similarly, another previous study showed the sequence-specific constraints of some TFs can decrease as a function of the number of co-occupying factors [27]. Although these motifs contain binding preferences of well-characterized TFs, most of them are novel, lacking any database matches. As a resource to the community, we provide all de novo discovered motifs in MEME Minimal Motif Format (Additional file 4).

### GWAS SNPs significantly overlap SEs and alter motifs outside of DHSs

**Figure 7:**
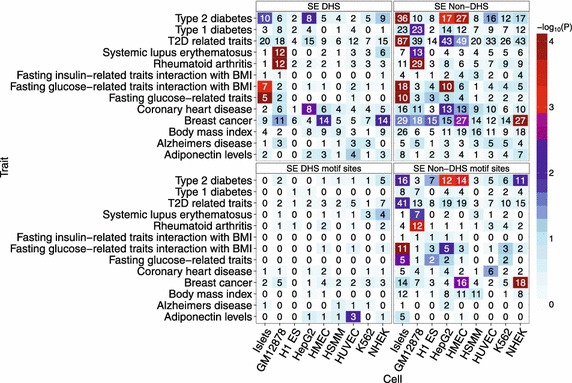
SEs show significant enrichment of GWAS SNPs associated with diseases or quantitative in a cell-specific manner. Positions of index and tightly linked (r^2^ ≥ 0.8) SNPs for different diseases or traits (y-axis) are overlapped with those of DHSs within SEs, non-DHSs with SEs, and motif sites in either DHSs or non-DHSs within SEs for each cell type (x-axis). Only motifs from the cell-specific motif sets used to generate the motif density tracks in Figure 4 are considered for each cell type. Motif sites were identified with FIMO at the default threshold of p < 10^−4^. *Text* in the *boxes* indicates the number of overlapping SNP loci in each cell type. *Shading* indicates the significance of SNP locus enrichment relative to a null distribution (“Methods”). Only SNPs that meet the genome-wide significance threshold (p < 5 × 10^−8^) are considered.

Motivated by the co-occurrence of T2D GWAS SNP loci and islet SEs, we test whether GWAS SNPs associated with several diseases and traits are generally enriched in SEs. A SNP locus consists of a lead SNP and all SNPs in strong LD with that lead SNP (r^2^ ≥ 0.8), carefully accounting for the possibility that the lead SNP is not causative but is instead in LD with the true, causative SNP. Indeed, SNP loci are enriched both inside and outside of DHSs in SEs, replicating previous disease- and trait-associated SNP enrichment in cell-specific enhancer states [2, 28], including rheumatoid arthritis in GM12878 (Figure 7, Additional file 2: Figure S13). While we know that SEs are enriched for SNPs associated with complex diseases, the mechanism by which these SNPs contribute to disease risk is not clear. One reasonable mechanism is through altering TF binding sites in SEs. To assess this idea, we also test for GWAS SNP enrichment within cell identity motif sites in SEs. Interestingly, GWAS SNP loci are much more enriched and abundant in SE non-DHS cell identity motif sites than they are in SE DHS cell identity motif sites. We posit that our earlier proposition that SNPs are disrupting putative TF binding sites in less accessible chromatin portions of SEs may in fact be a prevalent mechanism for driving common diseases. A previous study suggested that most non-coding GWAS SNP loci intersect DHS regions [29], but did not consider motifs in SEs that reside outside DHSs, as we do here. Our findings are consistent with another study that found enhancer-associated chromatin marks can be more informative for tissue-specific disease SNP enrichments than DHSs can [30].

Although our analysis provides a possible mechanism for many disease SNPs, several SNPs are left unaccounted for. Notably, our motif sets are limited, including only motifs from two databases, which are far from complete and are missing motifs that were discovered through our de novo motif discovery analysis, for example. Our GWAS analysis also focuses on motifs of activators, which excludes TFs like CTCF whose binding motif is slightly enriched in enhancers. We also do not consider variants that generate new motif sites. A recent study, for example, showed that a small insertion introducing binding motifs can lead to the formation of an aberrant oncogenic super-enhancer [31]. Including these other pieces of information may account for the remaining SNP loci.

## Conclusions

In this study, we analyzed the motif architecture of SEs, which we hypothesized can distinguish SEs from TEs. In general, enhancers are highly enriched for TF binding sites, especially those corresponding to activators. SEs are characterized by multiple motif-rich DHSs in close spatial proximity, unlike shorter TEs which typically have at most one DHS. SEs also display much higher H3K27ac ChIP-seq signal, a marker for active enhancers. This result complements a recent study which found that enhancer reporter activity from sequences in H3K27ac peaks within super enhancers is considerably stronger than enhancer reporter activity from sequences in H3K27ac peaks outside of super enhancers [32]. We conjecture that the exceptional length of SEs is in part due to the spatial coordination of accessible chromatin where clusters of activators can bind. Expression of tissue-specific TFs, particularly activators, also correlates with presence of their binding sites in enhancers that are active in that same tissue. Although these TF binding sites are most enriched in the punctate DHSs, SEs contain dense clusters of motifs outside of their DHSs. Notably, the SE motif architecture within and outside DHSs is significantly correlated, suggesting an orchestrated mechanism of regulation across the entire length of the element.

Through de novo motif discovery analysis in the motif-rich DNase I footprint sequences, we identified non-canonical binding sites and predicted which pairs of TFs bind adjacently more than expected by chance. Many of these composite motifs are not present in any motif database, having only been identified in this study, revealing large gaps in current databases.

Disease-associated SNPs identified through GWAS are known to co-localize with SEs in a cell-specific manner, but the exact mechanism by which these SNPs perturb genome function is not well characterized. One possibility is that these SNPs are disrupting TF binding in regions of open chromatin. Indeed, we find that DHSs within SEs are modestly enriched for GWAS SNPs. Interestingly, however, our most notable finding is that GWAS SNPs more often co-localize outside of the SE DHSs, and motif sites that are within SEs but outside of DHSs capture many of these SNPs. This finding may explain why a recent large study reported better GWAS SNP associations with chromatin-marked enhancers versus DHSs [30].

The functional role of these motif sites in less accessible chromatin is not well-understood. They could represent actual binding sites, despite lacking the extreme chromatin openness marked by DHSs. From an evolutionary perspective, it is possible that common disease SNPs may be preferentially found in such sites because disruption of TF binding in the most highly accessible chromatin would not be normally tolerated in the population due to selective pressure. Another possibility is based on the idea that SEs are lineage-specifying regulatory hubs that orchestrate a chromatin environment that is permissive to rapid cellular response. The GWAS SNPs enriched in motifs in SEs but outside DHSs could represent response elements that become active (and potentially hypersensitive to DNase I) in different developmental stages or under different physiologic conditions. Such a scenario would be an efficient solution to tune dynamic changes rapidly and precisely in lineage-specific gene expression, consistent with the rheostat model of SE function that was recently proposed [33]. Testing these hypotheses will require additional TF profiling experiments, such as ChIP-seq and DNase-seq, across diverse environmental, developmental, and genetic backgrounds.

## Methods

### DNase I hypersensitivity, chromatin state and gene expression profiling

Chromatin states and gene expression were integrated as previously described [3]. Sources of sequencing reads from ChIP-seq and RNA-seq experiments used in the integrative analysis are found in the supplementary materials of [3]. Single-hit DNase-seq data from the ENCODE Duke University group were used for the genome browser shots and calling DHSs. DHSs were based on narrow peak calls of single-hit DNase-seq data from the ENCODE Duke University group. Narrow peak files also contain the genome coordinates of the summits. Wiggle signal tracks for these single-hit data were also used for the UCSC genome browser shots.

### Motif scanning and calculating motif enrichment

We performed position weight matrix (PWM) motif scanning of FASTA sequence sets using FIMO [34]. Motif occurrences were called at the default p value threshold of 10^−4^. Based on empirical results, we found the default threshold to be a good compromise between a stringent threshold that called very few motif sites and a relaxed threshold that called too many motif sites, as well as computationally efficient. Motif enrichment in a set of sequences is calculated as the ratio of the number of occurrences of the motif in the set of sequences relative to the number of motif occurrences in dinucleotide shuffled versions of the sequences. Dinucleotide shuffled sequences were generated using the dinucleotide shuffling script in the MEME Suite [35]. This measure of enrichment allows direct comparison between sequence sets of varying number of nucleotides.

We also performed PWM scanning using CentriMo [36], which computes the central enrichment of motifs. For each motif, CentriMo finds an optimal score above the minimum threshold 5 bits at which to call motifs. 1 kbp sequences centered on DNase-seq peak summits in SEs or TEs were extracted from the hg19 reference genome as input sequences to CentriMo. CentriMo outputs log adjusted p-values measuring the significance of motif enrichment in the center of sequences.

When scanning sequences with known motifs, we take human or mouse motifs from the JASPAR 2014 [37] or the high-throughput SELEX [20] databases. We selected these two databases because of the quality of their binding models and their coverage of TFs. These two databases contain 943 motifs altogether.

### Motif enrichment heatmaps

Heatmaps plotting the enrichment of motifs in enhancer sequences were generated on a subset of the 943 motifs to focus on motifs that likely play an important role in enhancers and improve the visualization of the heatmaps. From the original 943 database motifs, we selected motifs that are highly (log_2_ fold enrichment >1.5 in at least one cell type) and differentially (log_2_ fold enrichment range >0.75) enriched in SE DHS sequences. We also select motifs that correspond to TFs that are expressed in any one of the nine cell types (>2 RPKM). Finally, we further condense the motifs by selecting motifs whose enrichment in SE DHS sequences correlates positively (ρ > 0) with the expression of the TF it corresponds to (Figure 2a), which we found to be an indication of activators. The remaining motifs are ordered using agglomerative clustering on the log_2_ fold enrichment values in SE DHSs. Agglomerative clustering was implemented using the aggExCluster method in the apcluster R package with the mutual pairwise similarities of data vectors as negative distances [38]. Visualization and clustering were based on log_2_ fold enrichment values instead of directly on fold enrichment values due to the relatively large range of fold enrichment values across all motifs (typically between 1 and 8). Using log_2_ values makes direct comparisons between motifs more manageable across this range.

### Assigning motifs to cells

For each cell type, we generated a list of activator motifs that are important in enhancers. These are the same motifs that we used for our motif density tracks (Figure 4b, Additional file 2: Figure S11) and GWAS enrichment analysis (Figure 7, Additional file 2: Figure S13). The selection procedure is similar to the filtering steps for the motif enrichment heatmaps. A motif is assigned to a cell type if its respective TF is expressed in that cell type (>2 RPKM), it is highly enriched in the central region of the cell’s SE DHS sequences (CentriMo log adjusted p-value < −50), and its enrichments in SE DHS sequences correlate positively with gene expression across the nine cell types (ρ > 0). If a motif has multiple versions in the databases, such as the DNA binding domain and full transcript versions in the high-throughput SELEX database [20] we only select the motif with the lowest CentriMo log adjusted p-value. The number of motifs in each set ranges from as few as 25 for H1 ES to as many as 56 for HMEC and NHEK.

### Aggregate histogram plots

We used the Homer annotatePeaks tool [39] to generate aggregate histogram plots documenting the dinucleotide frequency, motif density, and DNase-seq and H3K27ac ChIP-seq tag density ±1,500 bps with 10 bp bins relative to DNase-seq narrow peak summits overlapping SEs or TEs. ChIP tags were centered based on their estimated ChIP-fragment length. DNase tags were kept in their original positions by fixing the estimated DNase-fragment length to 1. Mappability (36 bp) and GERP [12] aggregate histogram plots were similarly generated with the bwtool aggregate command [40] instead.

### De novo footprinting

De novo footprinting requires deep sequenced DNase-seq data. Unfortunately, the data available are highly heterogeneous. Not only are the deep sequence DNase-seq datasets available at varying sequence depth, they are also generated by two different experimental protocols: a single-hit version [8] and a double-hit version [25] of DNase-seq. Therefore, we adopted four different methods to call footprints de novo.

Four of the nine cell types considered in this study have deep sequenced double-hit DNase-seq data (K562, HSMM, HepG2, HUVEC). Of these four, three were previously processed by a de novo footprinting algorithm that searches DHSs for locations with high footprint occupancy scores, and genome coordinates of the footprints were made openly available [24]. For the remaining cell type, HUVEC, footprints were called on the double-hit data with Wellington [41] at a stringent threshold of p < 10^−20^.

For the remaining five cell types, footprints were called on deep sequenced single-hit DNase-seq data, but these datasets were not sequenced as deeply as the double-hit for the other four cell types. We called footprints in four of the single-hit DNase-seq data with the “1D” version of Wellington on pooled replicates. Footprints were called at a threshold of p < 10^−5^, except for the GM12878 cell line for which we used a threshold of p < 10^−10^ due to the deeper sequencing depth available. For the remaining cell line, NHEK, the sequencing depth of the available dataset was too shallow to reliably call footprints with Wellington, so we extracted only high confidence footprints previously called by a Hidden Markov Model (HMM) algorithm [23], which were originally based in the hg18 genome but later lifted over to the hg19 genome.

Footprints are further processed prior to downstream analysis by extending footprint genome coordinates equally on both ends by 5 bps (20 bps for HMM-based footprints) and then merging overlapping coordinates using the BEDTools [42] mergeBed tool with parameter d = 10 (d = 40 for HMM-based footprints).

Of the nine cell types considered in this study, only the K562 cell line had enough data to apply all four footprinting methods on. Hence, we applied all four footprinting methods on the K562 for comparison in the downstream de novo motif discovery (see next subsection). The thresholds and parameters were selected to yield sequence datasets of 5–10 million bps, which we found to be optimal for motif discovery.

### De novo motif discovery

De novo motif discovery was applied to SE DHS sequences and enhancer footprint sequences in all enhancers using the EXTREME algorithm [19]. FASTA sequence sets were generated by extracting hg19 masked genome sequences from BED file coordinates using the BEDTools fastafrombed command [42]. SE DHS FASTA sequences were further preprocessed by replacing instances of AAAAAAAA, ACACACAC, and their reverse complements with capital N’s. Such repetitive subsequences are ubiquitous throughout the genome and are often missed in the genome masking process. Masked sequences were inputted to the EXTREME pipeline with the parameters l = 5 and q = 0.02 (all other parameters were set to the default or recommended values). We selected these parameters empirically based on the quality of motifs generated. Although footprints were generated from a variety of algorithms and experimental protocols, EXTREME can still find a consistent set of high-quality enhancer-associated motifs (Additional file 2: Figure S12). Discovered motifs were compared to known motifs from the JASPAR 2014 [37] or the high-throughput SELEX [20] databases using TOMTOM [43] keeping only significant matches (*E* < *0.1*).

### GWAS variant enrichment

We calculated GWAS variant enrichment exactly as we did in our previous study of SEs [3] on an updated set of SNPs from the NHGRI GWAS catalog (http://www.genome.gov/gwastudies/; downloaded on October 9, 2014). Briefly, we calculated enrichment by performing a permutation test that measures SNP loci and enhancer overlaps as previously described [2]. A SNP locus consists of a lead SNP and all SNPs in strong LD with that lead SNP. SNPs in LD with the lead SNP were defined as those with r^2^ ≥ 0.8. We ran 10,000 iterations of the permutation test and estimated the maximal P-value as the number of permutations equal to or greater than the observed overlap value plus one divided by the number of iterations plus one (10,001). Our enrichment analysis was performed on both the entire set of NHGRI GWAS catalog SNPs per trait and a filtered subset of genome-wide significant SNPs (p < 5 × 10^−8^) per trait.

## Additional files

Additional file 1:
**Table S1.** Staitistics of enhancers. Additional file 2: Supplementary Figures S1 to S13. Additional file 3: CentriMo scans of DHS sequences with database motifs. Zip archive of text outputs from CentriMo scans on DHS sequences within SEs or TEs using database motifs. File names specify the cell type and enhancer class. Can be downloaded at http://fusion.nhgri.nih.gov/files/se-motifs/AdditionalFile3.zip. Additional file 4: De novo motif disocvery results. Zip archive of *de novo* motif discovery results. Discovered motifs are condensed in MEME Minimal Motif Format. TOMTOM output HTML files documenting the comparison of the discovered motifs to known motifs and CentriMo output text files detailing the enrichment of the discovered motifs in SE or TE DHS sequences are also included. File names indicate the type of sequences used for the motif discovery, including the method of footprinting. Can be downloaded at http://fusion.nhgri.nih.gov/files/se-motifs/AdditionalFile4.zip.

## Abbreviations

SE: stretch enhancer
TE: typical enhancer
CE: concatenated enhancer
DHS: DNase I hypersensitive site
FP: footprint
TF: transcription factor
TSS: transcription start site
bp: base pair
T2D: type 2 diabetes
H3K27ac: histone 3 lysine 27 acetylation
GWAS: genome-wide association study
SNP: single nucleotide polymorphism
LD: linkage disequilibrium
PWM: position weight matrix
HMM: Hidden Markov Model
GERP: Genomic Evolutionary Rate Profiling
