# Supplementary material for: Motif signatures in stretch enhancers are enriched for disease-associated genetic variants
Source: Epigenetics Chromatin. 2015 Jul 16;8:23. doi: 10.1186/s13072-015-0015-7 (PMC4502539; doi:10.1186/s13072-015-0015-7)
Supplement: Additional file 2: — Supplementary Figures S1 to S13. [file 13072_2015_15_MOESM2_ESM.pdf]

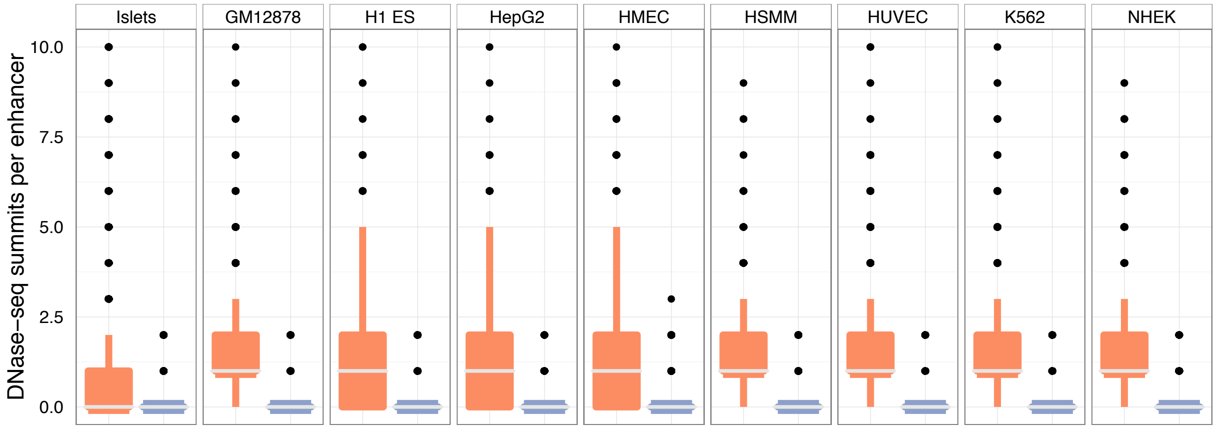

Figure S1. Comparison of DNase-seq summit frequency per SE and TE shows that SEs are more enriched in DHSs than TEs are for all 9 cell types (two-tailed Wilcoxon rank sum test test, $p < 10^{-100}$ for all cell types). Boxplots are colored by enhancer type according to the same coloring scheme in **Figure 1**. Whiskers extend to 1.5x the interquartile range and outliers are shown as block dots, but the y-axis is truncated so that the boxplots can remain in view.

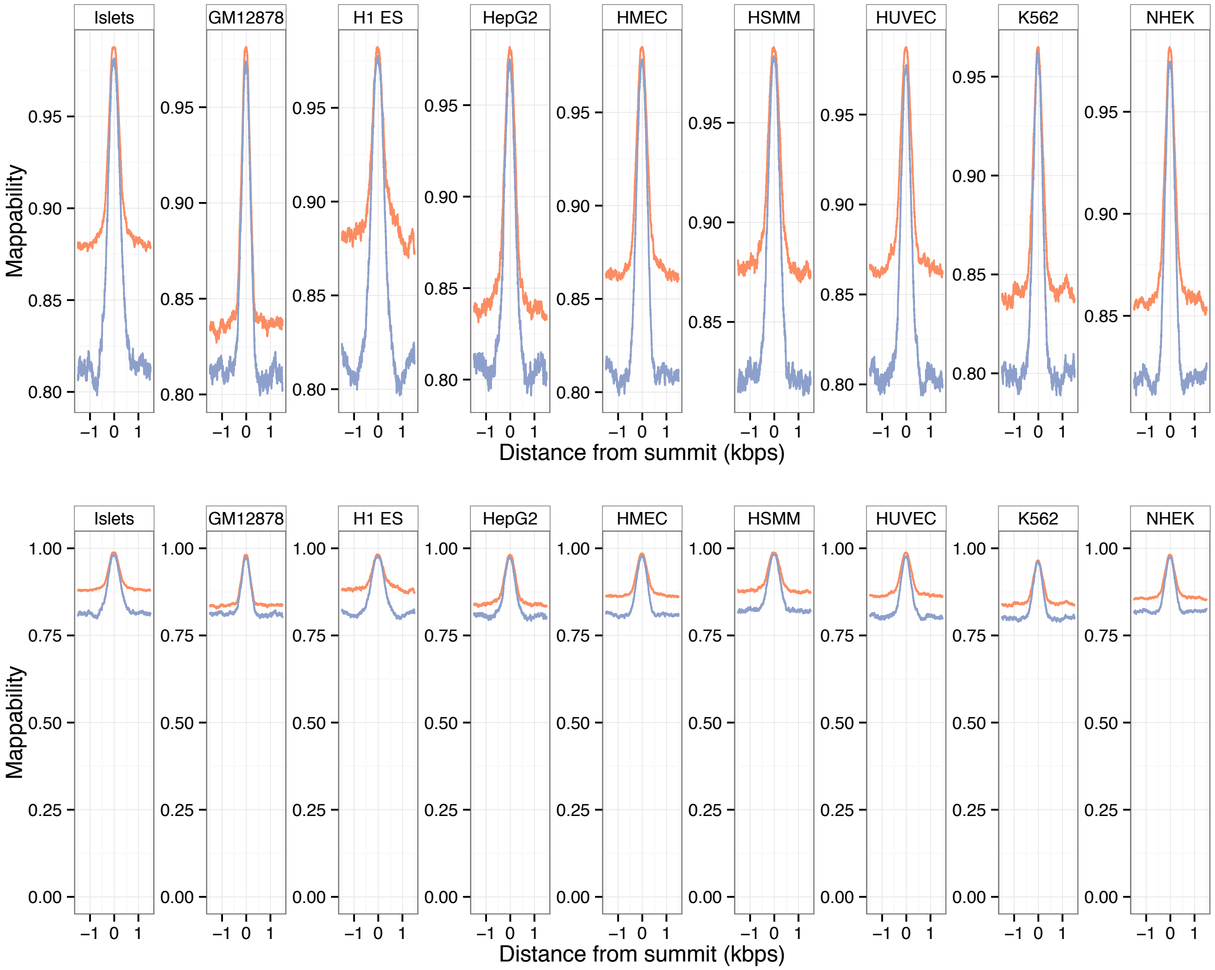

Figure S2. Comparison of aggregate mappability profiles of sequences centered on DNase-seq summits located in SEs or TEs reveals that SEs are generally more mappable than TEs, especially in the regions flanking DNase-seq summits. The top plots display free scales for each cell type, while the bottom plots display the same data with the y-axis fixed between 0 and 1.

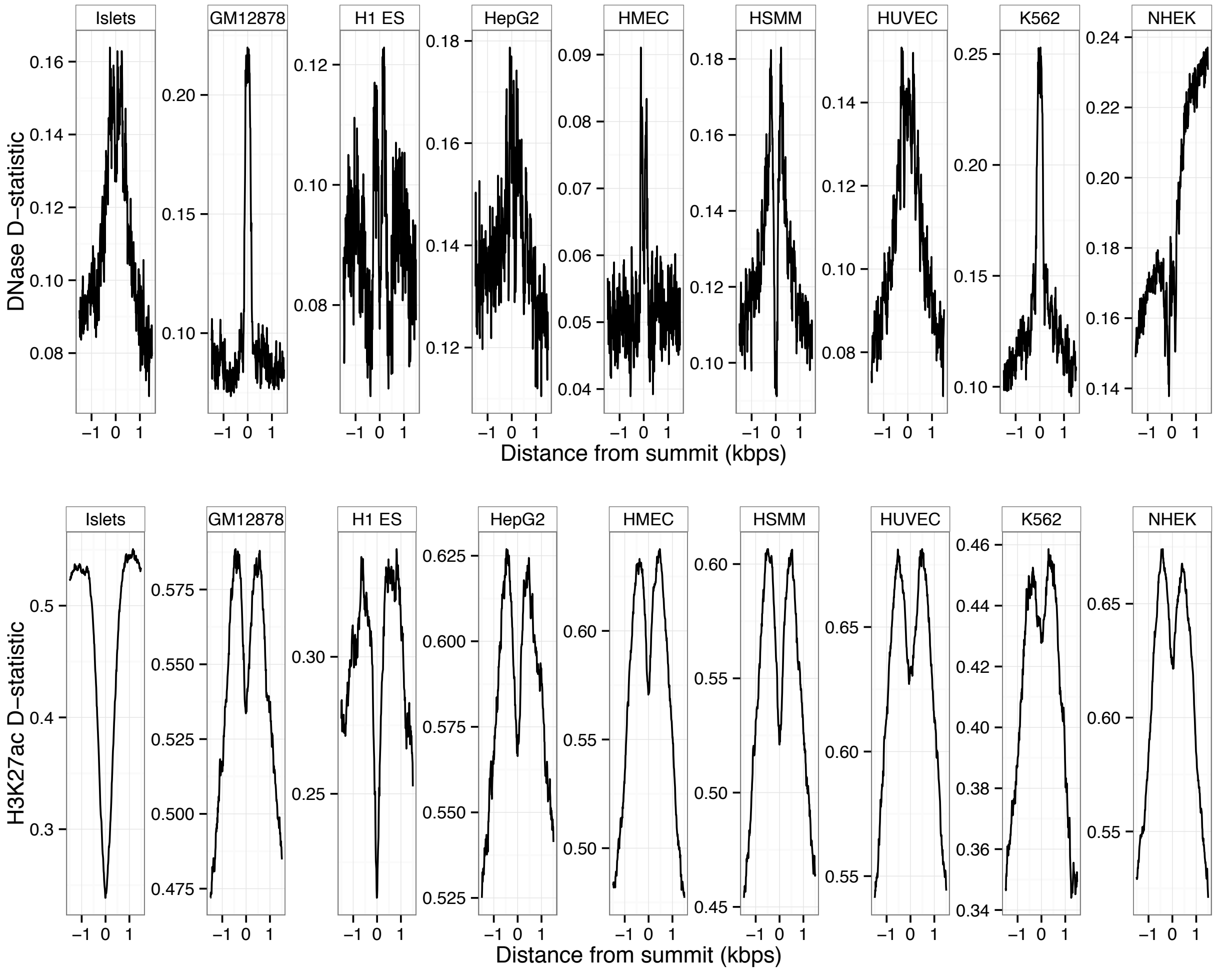

Figure S3. Measuring statistical significance of difference in DNase-seq (top) and H3K27ac ChIP-seq (bottom) tag density along 3kb sequences centered on DNase-seq summits in SEs vs. TEs. SEs and TEs clearly show significant differences in H3K27ac ChIP-seq tag density along the entire length of the sequences; this difference is strongest about 100 bps upstream and downstream from the center, but the difference dips in the center. The center is the location of greatest chromatin accessibility, which is where TFs can bind and displace histones, lowering the presence of H3K27 acetylation and mitigating the difference in H3K27ac ChIP-seq tag density, resulting in the dip. Statistical significance is measured with the two-sided Kolmogorov-Smirnov test D-statistic.

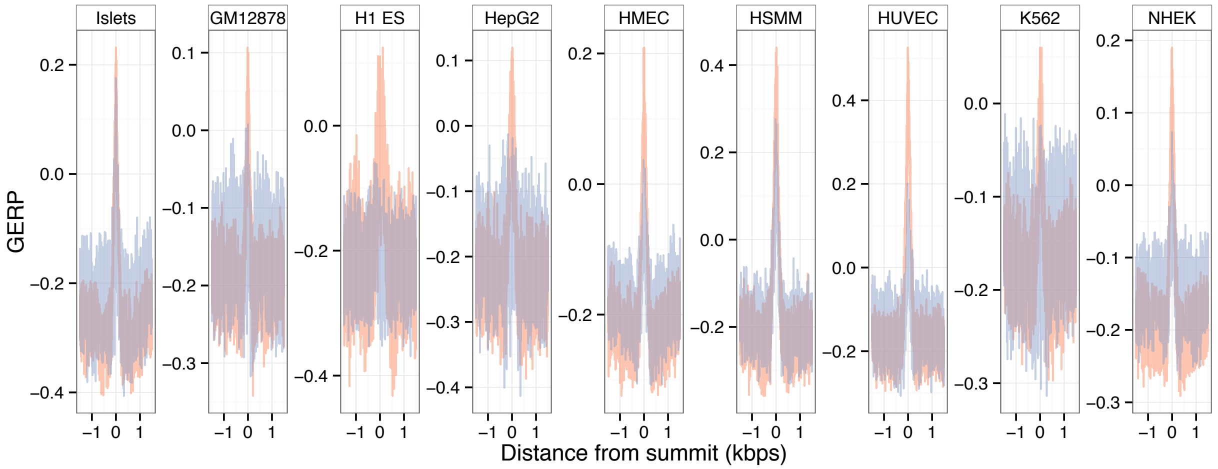

Figure S4. Aggregate GERP scores along 3kbp sequences centered on DNase-seq summits located within SEs or TEs. SEs are typically more evolutionary conserved than SEs at the summit of DNase-seq peaks, but this relationship is generally reversed in the flanking sequences.

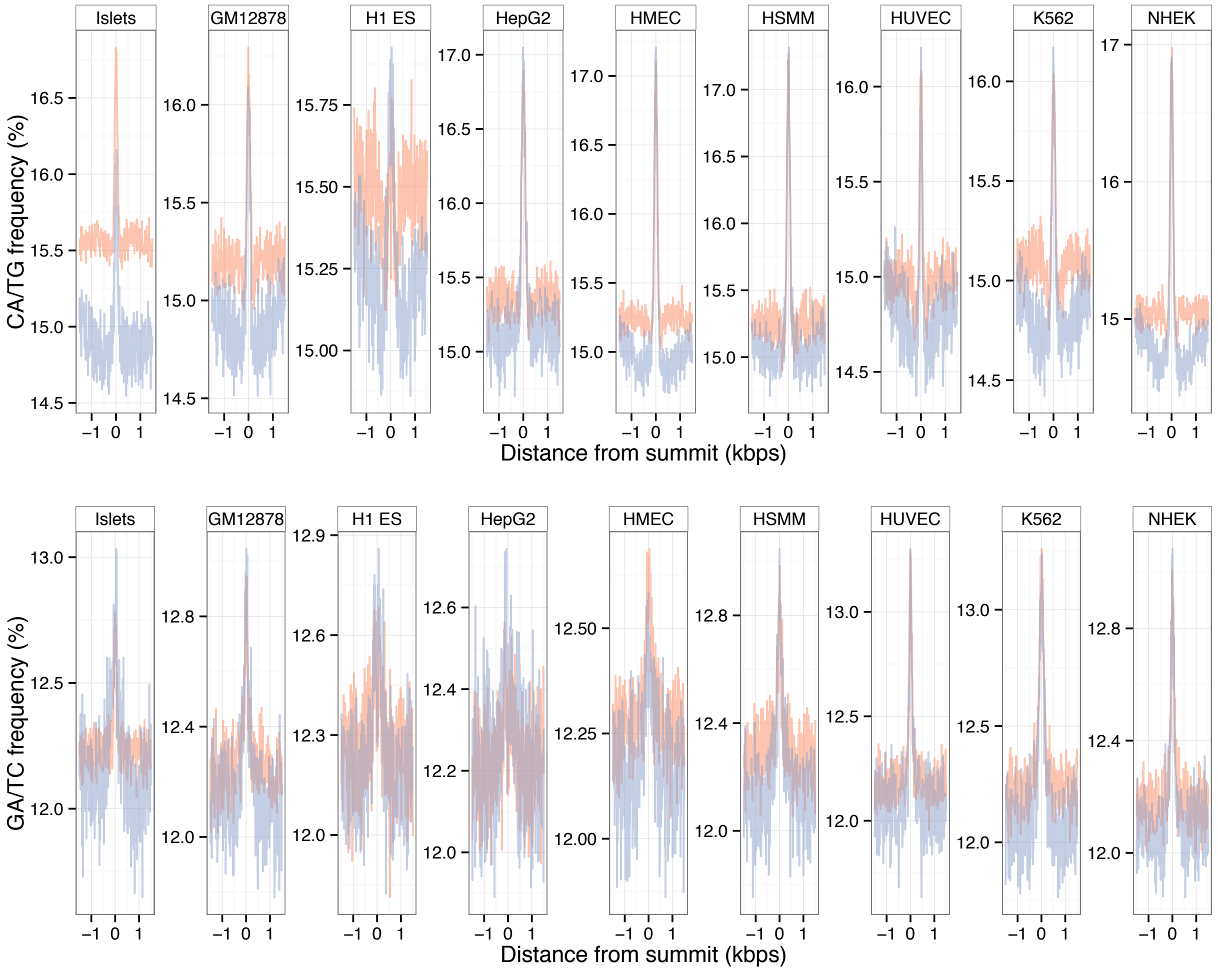

Figure S5. Aggregate CA (top) and GA (bottom) dinucleotide frequencies of sequences centered on DNase-seq summits located in SEs or TEs.

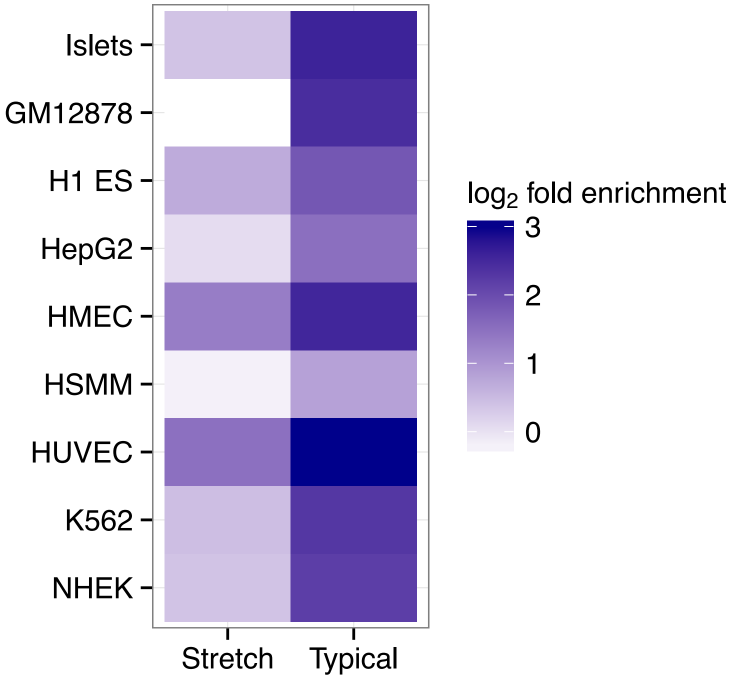

Figure S6. DNase-seq summits in TEs overlap CpG islands (unmasked) more often than DNase-seq summits in SEs do. Null sets were generated by randomly shuffling summit locations in the genome 10,000 times with the Pybedtools intersection_matrix command. Enrichments were calculated by the ratio of observed TE/SE DNase-seq summits and CpG islands overlaps in each cell type to the median number of overlaps in the respective null sets.

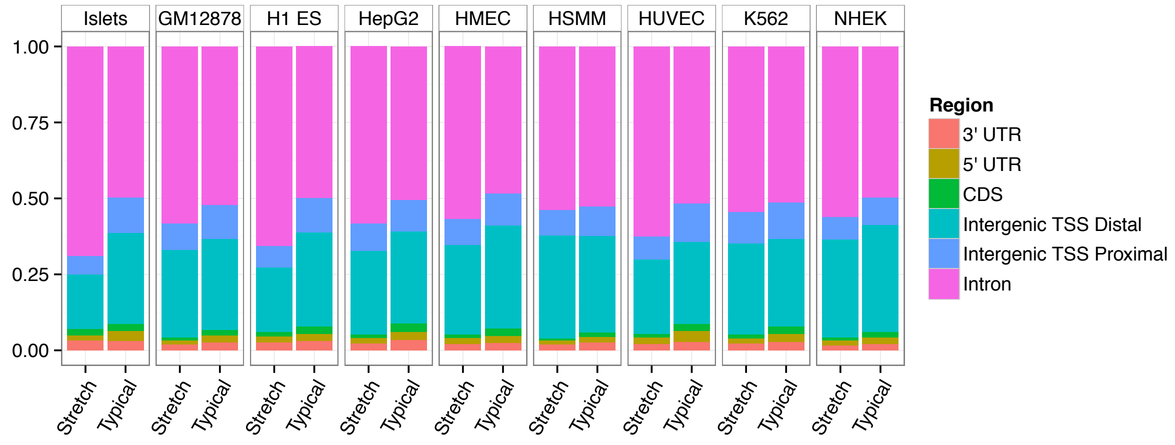

Figure S7. DNase-seq summits are distributed differently throughout the genome based on the type of enhancer they overlap. SE DNase-seq summits show a very strong preference for introns. Gene annotations are based on GENCODE v19 for the hg19 human reference genome.

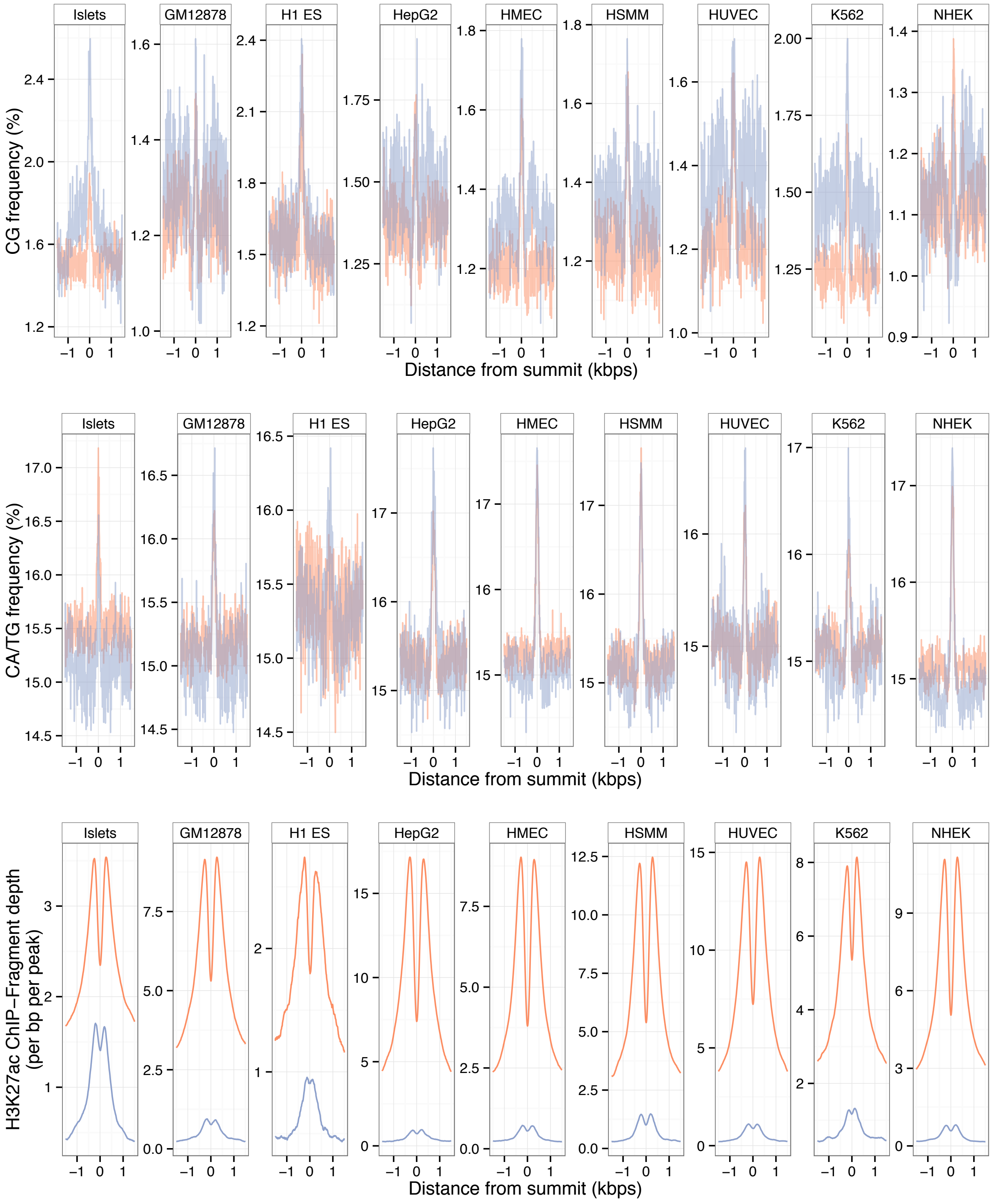

Figure S8. CA and CG dinucleotide differences are not as strong when restricted to DNase-seq summits located in intergenic TSS distal regions; however, the stark contrast in H3K27 acetylation is preserved.

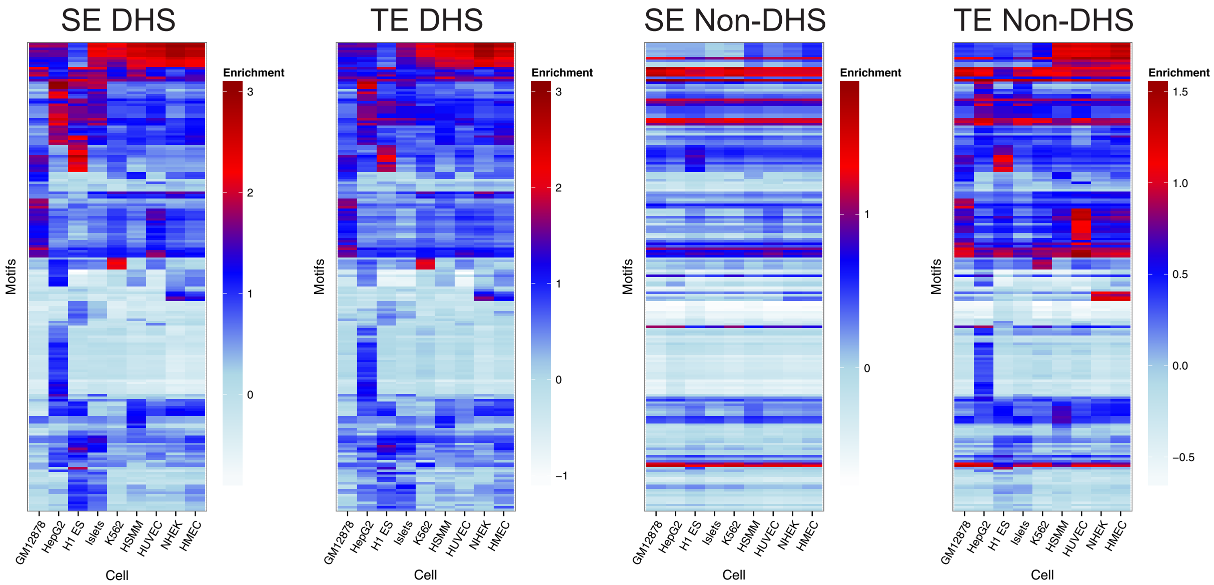

Figure S9. Heatmaps of motif enrichments in each of the 4 regions considered across the 9 cell types. The data are the same as shown in **Figure 3**, except that the heatmaps do not share the same coloring scale.

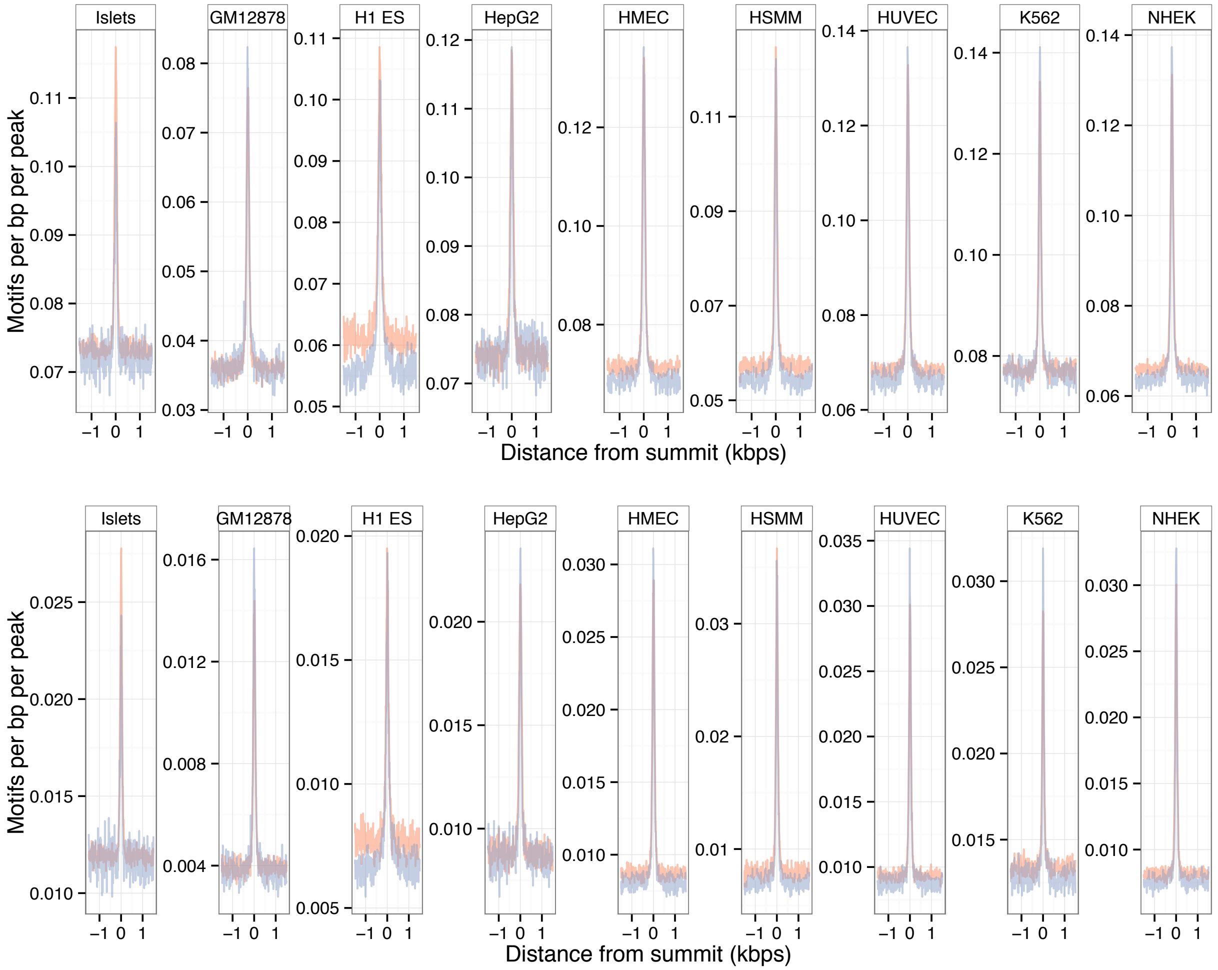

Figure S10. Histogram plots of motif density relative to DNase-seq peak summits at different thresholds. Motifs were called at 30% (top) or 50% (bottom) of the maximum log likelihood score. The same sets of motifs were used as those used to generate **Figure 4A**. Despite the different thresholds used to call motifs, the relative enrichments with respect to nucleotide position remain generally the same.

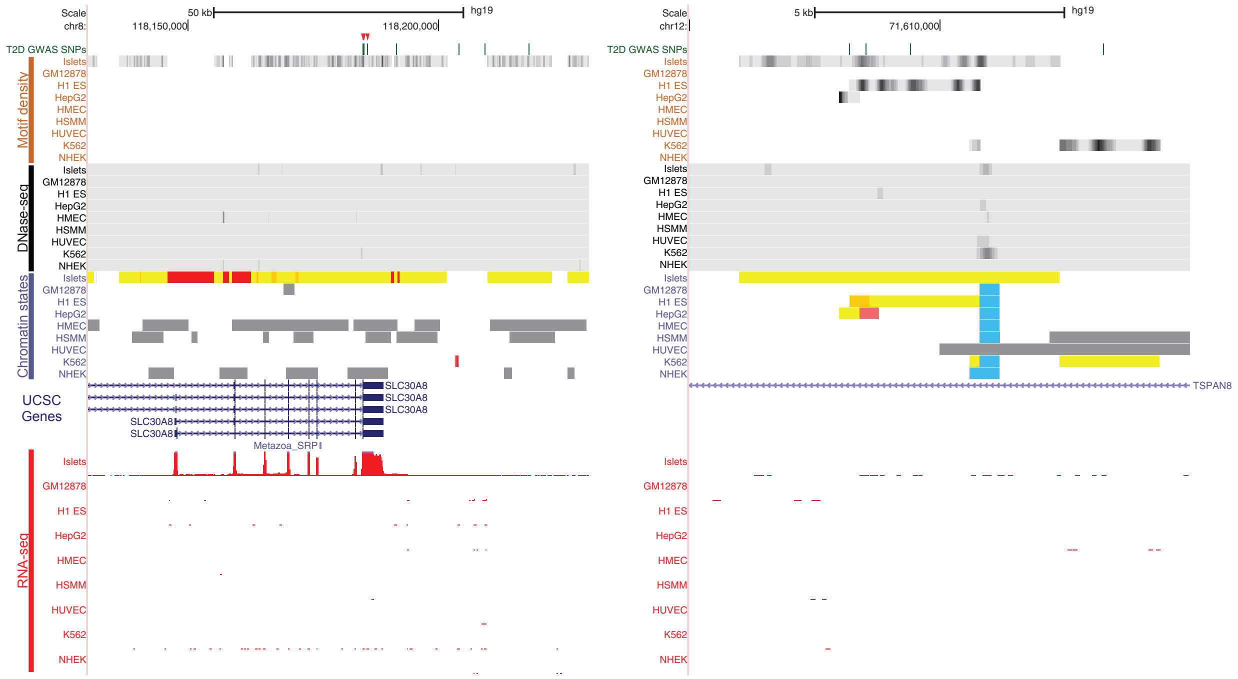

Figure S11. UCSC Genome browser view of *SLC30A8* (left) and *TSPAN8* (B) loci. Locations of annotated genes are indicated in the UCSC Genes portion of the view. Index and tightly-linked ($r^2 \geq 0.8$) SNPs associated with T2D are indicated in green in the T2D GWAS SNPs track. Index SNPs rs13266634, rs3802177, rs11558471 are marked with red arrows in the left view. No index SNPs are within view in the right browser shot, but the SNPs in the *TSPAN8* loci are tightly linked with index SNP rs4760790. Motif density tracks show the frequency of motif sites in 150 bp windows at 10 bp steps within sequences assigned enhancer states. Motifs are assigned to cell types based on the expression of the respective TFs and enrichment in SE DHS sequences (Materials and Methods). Chromatin state colors and scales are assigned as in Figure 1A. RNA-seq data (red) demonstrates the robust islet-specific expression of *SLC30A8*.

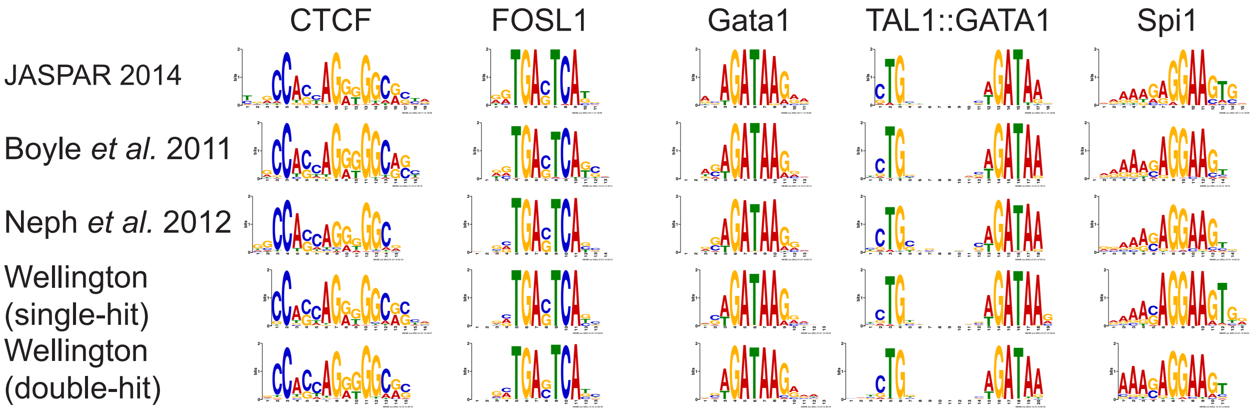

Figure S12. EXTREME *de novo* motif discovery is robust with respect to the method of *de novo* footprinting. Despite the difference in algorithms and experimental protocols used for the footprinting, EXTREME is consistent in recovering the most prominent motifs in K562 enhancers. Sequence logos and their internal names in the JASPAR 2014 vertebrates database are displayed in the top row.

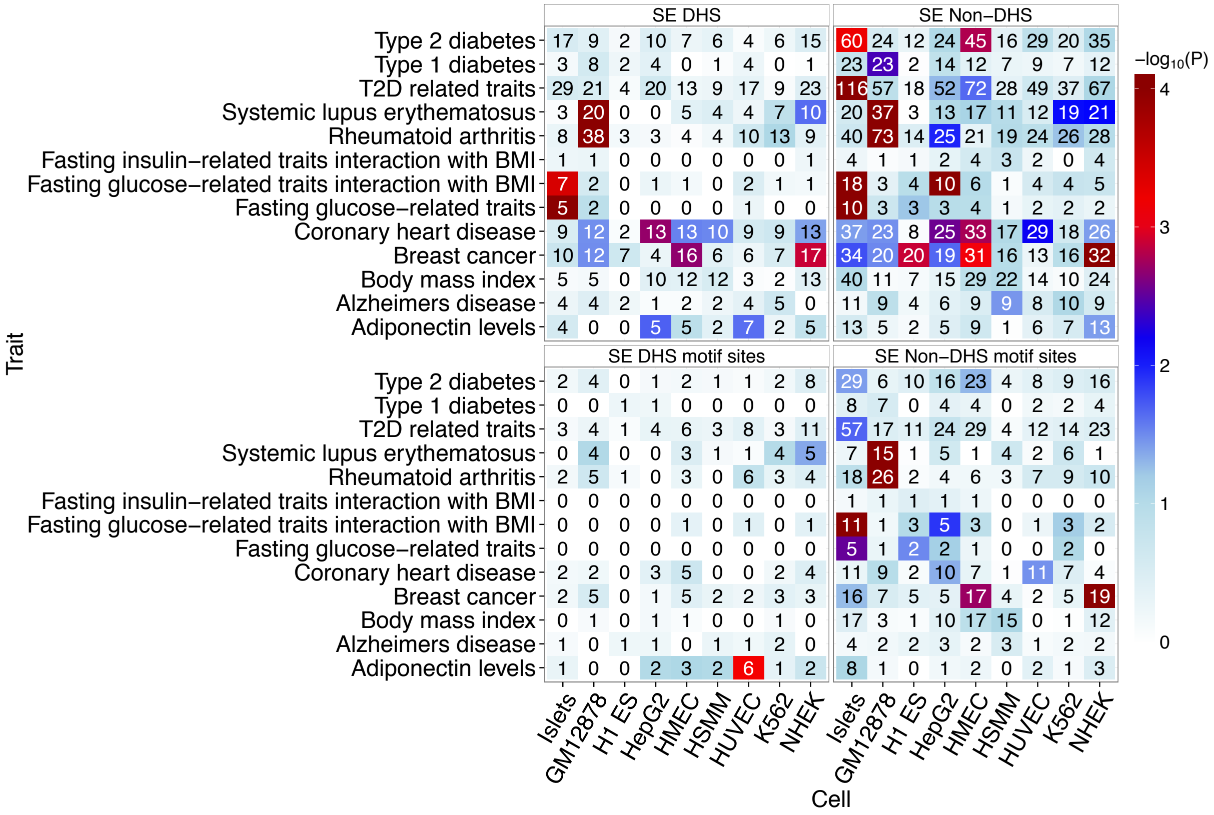

Figure S13. GWAS SNP loci associated with diseases or traits are enriched in various portions of SEs in a cell specific manner. Four different types of regions are considered: portions of SEs also labeled as DHSs, portions of SEs not labeled as DHSs, and motif occurrences in one of these two regions. Positions of index and tightly linked ($r^2 \geq 0.8$) SNPs for different diseases or traits (y-axis) are overlapped with the four different regions for each cell type (x-axis). Numbers in the boxes indicate the number of SNP loci overlapping enhancer states in each cell type. Coloring indicates the significance of SNP locus enrichment relative to a null distribution (**Materials and Methods**). All GWAS SNPs associated with these diseases or traits in the entire NHGRI Catalog of Published Genome-Wide Association Studies are considered in this analysis.
