# Supplementary material for: Motif signatures in stretch enhancers are enriched for disease-associated genetic variants
Source: Epigenetics Chromatin. 2015 Jul 16;8:23. doi: 10.1186/s13072-015-0015-7 (PMC4502539; doi:10.1186/s13072-015-0015-7)
Supplement: Additional file 4: — De novo motif disocvery results. Zip archive of de novo motif discovery results. Discovered motifs are condensed in MEME Minimal Motif Format. TOMTOM output HTML files documenting the comparison of the discovered motifs to known motifs and CentriMo output text files detailing the enrichment of the discovered motifs in SE or TE DHS sequences are also included. File names indicate the type of sequences used for the motif discovery, including the method of footprinting. Can be downloaded at http://fusion.nhgri.nih.gov/files/se-motifs/AdditionalFile4.zip. [file 13072_2015_15_MOESM4_ESM.zip › AdditionalFile4/Islets_StretchEnhancersDHS_TOMTOM.html]

TOMTOM


The name of the query motif.

[close ]

The alternate name of the query motif.

[close ]

A link to more information about the query motif.

[close ]

The motif preview. On supporting browsers this will display as a motif
logo, otherwise the consensus sequence will be displayed.

[close ]

The number of significant matches of the query motif to a motif in
the target database.

[close ]

Links to the first 20 matches of the query motif to a motif in the
target database.

[close ]

The database name.

[close ]

The number of motifs read from the motif database minus the number that
had to be discarded due to conflicting IDs.

[close ]

The number of motifs that had a match with at least one of the query
motifs.

[close ]

The summary gives information about the matched motif. Mouse over each
row to show further help buttons for each specific title.

[close ]

The name of the matched motif.

[close ]

The alternative name of the matched motif.

[close ]

The database containing the matched motif.

[close ]

The probability that the match occurred by random chance according to the null model.

[close ]

The expected number of false positives in the matches up to this point.

[close ]

The minimum **F**alse **D**iscovery **R**ate required to include the match.

[close ]

The number of letters that overlaped in the optimal alignment.

[close ]

The offset of the query motif to the matched motif in the optimal alignment.

[close ]

The orientation of the matched motif that gave the optimal alignment.
A value of "normal" means that the matched motif is as it appears in
the database otherwise the matched motif has been reverse complemented.

[close ]

The image shows the alignment of the two motifs. The matched motif is
shown on the top and the query motif is shown on the bottom.

[close ]

By clicking the link "Create custom LOGO ↧" a form to make custom logos
will be displayed. The download button can then be clicked to generate a motif
matching the selected specifications.

[close ]

Two image formats, png and eps, are avaliable. The pixel based portable
network graphic (png) format is commonly used on the Internet and the
Encapsulated PostScript (eps) format is more suitable for publications
that might require scaling.

[close ]

Toggle error bars indicating the confidence of a motif based on the
number of sites used in its creation.

[close ]

Toggle adding pseudocounts for **S**mall **S**ample
**C**orrection.

[close ]

Toggle a full reverse complement of the alignment.

[close ]

Specify the width of the generated logo.

[close ]

Specify the height of the generated logo.

[close ]

[close ]

[close ]

# Tomtom

## Motif Comparison Tool

For further information on how to interpret these results or to get a
copy of the MEME software please access
http://meme.nbcr.net.

If you use TOMTOM in your research, please cite the following paper:  
Shobhit Gupta, JA Stamatoyannopolous, Timothy Bailey and William Stafford Noble,
"Quantifying similarity between motifs",
*Genome Biology*, **8**(2):R24, 2007.
[full text]

Query Motifs  |  Target Databases  |  Matches  |  Program information

|  |  |
| --- | --- |
| Query Motifs | Next Top |

| Name | Alt. Name | Preview | Matches | List |
| --- | --- | --- | --- | --- |
| M1 | O1 | CAGTGACTCAGCC | 10 | JASPAR2014\_FOSL1,  JASPAR2014\_JUND,  JASPAR2014\_NFE2::MAF,  JASPAR2014\_FOS,  JASPAR2014\_Bach1::Mafk,  JASPAR2014\_JUN::FOS,  JASPAR2014\_JUNB,  JASPAR2014\_Nfe2l2,  JOLMA2013\_MAFK\_full\_2,  JASPAR2014\_FOSL2 |
| M2 | O2 | GGCTGAGTCACAG | 9 | JASPAR2014\_JUND,  JASPAR2014\_FOS,  JASPAR2014\_FOSL1,  JASPAR2014\_FOSL2,  JASPAR2014\_Meis1,  JASPAR2014\_JUNB,  JOLMA2013\_MAFK\_full\_2,  JASPAR2014\_JUN::FOS,  JASPAR2014\_NFE2::MAF |
| M3 | O3 | AAAATGACTCATTT | 10 | JOLMA2013\_JDP2\_full,  JOLMA2013\_JDP2\_DBD,  JOLMA2013\_Jdp2\_DBD,  JASPAR2014\_BATF::JUN,  JASPAR2014\_JUNB,  JASPAR2014\_FOS,  JOLMA2013\_NFE2\_DBD,  JASPAR2014\_FOSL2,  JASPAR2014\_JUN::FOS,  JASPAR2014\_JUND |
| M4 | O4 | GGCCCTGCCTGCCTG | 0 |  |
| M5 | O5 | GGGCCCAGCCCCA | 2 | JASPAR2014\_TFAP2C,  JASPAR2014\_SP1 |
| M6 | O6 | CAGGGCTGGGCTGGG | 0 |  |
| M7 | O7 | TAAAAATAAATAA | 5 | JOLMA2013\_FOXL1\_full\_2,  JOLMA2013\_FOXC1\_DBD,  JOLMA2013\_FOXC2\_DBD\_2,  JOLMA2013\_ONECUT3\_DBD,  JOLMA2013\_ONECUT1\_DBD |
| M8 | O8 | ATCAAATATTTGCT | 3 | JOLMA2013\_FOXD2\_DBD,  JOLMA2013\_FOXD3\_DBD,  JOLMA2013\_FOXC1\_DBD\_2 |
| M9 | O9 | TTTTTAAAAAAAA | 0 |  |
| M10 | O10 | TTTTATTTTTAAA | 0 |  |
| M11 | O11 | TAAATAAATAAATA | 1 | JOLMA2013\_FOXC1\_DBD |
| M12 | O12 | CCTCCTCCTCCTC | 1 | JASPAR2014\_ZNF263 |
| M13 | O13 | CCCACCCCCACCCCA | 5 | JASPAR2014\_KLF5,  JASPAR2014\_E2F3,  JASPAR2014\_Klf4,  JASPAR2014\_SP2,  JASPAR2014\_RREB1 |
| M14 | O14 | TTTTAATAATTAAA | 2 | JASPAR2014\_HNF1A,  JASPAR2014\_HNF1B |
| M15 | O15 | AGTTATTTTTAGAA | 5 | JASPAR2014\_MEF2A,  JASPAR2014\_MEF2C,  JOLMA2013\_MEF2A\_DBD,  JOLMA2013\_MEF2D\_DBD,  JOLMA2013\_MEF2B\_full |
| M16 | O16 | CCCCCAGCCCCAGC | 2 | JASPAR2014\_EGR1,  JASPAR2014\_E2F3 |
| M17 | O17 | TTTAAAATTAAAAA | 0 |  |
| M18 | O18 | AGGGAGGGAGGGAGG | 3 | JASPAR2014\_EWSR1-FLI1,  JASPAR2014\_ZNF263,  JASPAR2014\_E2F6 |
| M19 | O19 | TCCTCCTCCTCCT | 1 | JASPAR2014\_ZNF263 |
| M20 | O20 | CCTGCCAGGCCTGGC | 0 |  |
| M21 | O21 | CCTGCCTGGGCCCTG | 0 |  |
| M22 | O22 | GGCAGGCCCGGGCTGG | 1 | JASPAR2014\_Klf4 |
| M23 | O23 | TTAAAAATAAAAA | 0 |  |
| M24 | O24 | CCTCCCCACCCCCCAG | 0 |  |
| M25 | O25 | TTTTAAAAATAAA | 0 |  |
| M26 | O26 | CACACAGACACAC | 0 |  |
| M27 | O27 | CCAGCCAGGCCTG | 0 |  |
| M28 | O28 | CTGCCTCCTGC | 0 |  |
| M29 | O29 | CACACACATGCACACAC | 0 |  |
| M30 | O30 | GGCAGGGGCCTGGGCAGGG | 2 | JASPAR2014\_SP2,  JASPAR2014\_SP1 |
| M31 | O31 | GCCATGGCAACAG | 10 | JASPAR2014\_RFX2,  JASPAR2014\_RFX5,  JASPAR2014\_Rfx1,  JOLMA2013\_Rfx2\_DBD,  JOLMA2013\_RFX2\_DBD,  JOLMA2013\_RFX3\_DBD,  JOLMA2013\_Rfx3\_DBD,  JOLMA2013\_RFX5\_DBD,  JOLMA2013\_RFX5\_DBD\_2,  JOLMA2013\_RFX4\_DBD |
| M32 | O32 | AATGAGTCATT | 8 | JOLMA2013\_JDP2\_full,  JOLMA2013\_Jdp2\_DBD,  JOLMA2013\_JDP2\_DBD,  JASPAR2014\_FOS,  JOLMA2013\_NFE2\_DBD,  JASPAR2014\_JUND,  JASPAR2014\_JUN::FOS,  JASPAR2014\_JUNB |
| M33 | O33 | CCAGGCCTGGG | 0 |  |
| M34 | O34 | CAGCAGCAGCAGG | 1 | JASPAR2014\_Tcf12 |
| M35 | O35 | CTGCCTCCTGCCTG | 0 |  |
| M36 | O36 | CCAGCCAGGCCCTGG | 0 |  |
| M37 | O37 | GCCCAGCCCTG | 0 |  |
| M38 | O38 | GAGAGAGAGAGAGA | 0 |  |
| M39 | O39 | TTAAAAATAAAAA | 0 |  |
| M40 | O40 | TATTATTATTAT | 0 |  |
| M41 | O41 | AAAATAAATAAA | 4 | JOLMA2013\_FOXC1\_DBD,  JOLMA2013\_FOXC2\_DBD\_3,  JOLMA2013\_FOXL1\_full\_2,  JASPAR2014\_FOXP1 |
| M42 | O42 | CCTGCCTGCCT | 0 |  |
| M43 | O43 | ATTTTTTAAAAA | 0 |  |
| M44 | O44 | AAAAACAAACAAA | 2 | JASPAR2014\_FOXP1,  JASPAR2014\_Foxd3 |
| M45 | O45 | TATAAATAAATAAAAA | 1 | JOLMA2013\_FOXC1\_DBD |
| M46 | O46 | GGAGGGAGGAGGAAG | 0 |  |
| M47 | O47 | TTAAAAAAGAAAAA | 0 |  |
| M50 | O50 | ACAGAGACAGAGAGAG | 0 |  |
| M52 | O52 | TTTTTCTTTTTTTAAAAAA | 0 |  |
| M53 | O53 | AAAAAAAATAAATAAAAAAAA | 2 | JOLMA2013\_ONECUT3\_DBD,  JOLMA2013\_ONECUT1\_DBD |
| M54 | O54 | AATATTTACTGT | 5 | JOLMA2013\_FOXD2\_DBD,  JOLMA2013\_FOXB1\_DBD\_2,  JOLMA2013\_FOXD3\_DBD,  JOLMA2013\_FOXC1\_DBD\_2,  JOLMA2013\_FOXC2\_DBD |
| M55 | O55 | AGTGACTCATT | 10 | JASPAR2014\_FOS,  JASPAR2014\_JUND,  JOLMA2013\_JDP2\_full,  JOLMA2013\_Jdp2\_DBD,  JOLMA2013\_JDP2\_DBD,  JASPAR2014\_JUNB,  JASPAR2014\_FOSL1,  JOLMA2013\_NFE2\_DBD,  JASPAR2014\_JUN::FOS,  JASPAR2014\_FOSL2 |
| M56 | O56 | AAATAAATAAATA | 1 | JOLMA2013\_FOXC1\_DBD |
| M57 | O57 | TGGCCAGCAGGGC | 0 |  |
| M58 | O58 | CCCTGGCCAGCTG | 0 |  |
| M59 | O59 | GGGAAACTGAGGC | 0 |  |
| M60 | O60 | CCCAGGCCTGGC | 0 |  |
| M61 | O61 | AGCCCAGCCCTGG | 0 |  |
| M62 | O62 | CAGCCCCTGCC | 0 |  |
| M63 | O63 | TTTTTTTAAAAAA | 0 |  |
| M64 | O64 | AAATGTTTTTTAAAA | 0 |  |
| M65 | O65 | AGAGAGAGAGAGAGAGA | 0 |  |
| M67 | O67 | CCTGCCAGGCCCCTCCT | 0 |  |
| M68 | O68 | AAAAGTAAATAAATACAT | 4 | JOLMA2013\_FOXC1\_DBD,  JOLMA2013\_Foxc1\_DBD,  JOLMA2013\_FOXC2\_DBD\_2,  JOLMA2013\_FOXC2\_DBD\_3 |
| M71 | O71 | TCTGTCCCAGGCCTGCCCTC | 0 |  |
| M74 | O74 | AAACATTTATTTTAAAAAAC | 0 |  |
| M75 | O75 | GCTGAGCTCAG | 1 | JOLMA2013\_Mafb\_DBD\_3 |
| M76 | O76 | CCCAGAGCTGG | 0 |  |
| M77 | O77 | AATGACTCATT | 6 | JOLMA2013\_JDP2\_full,  JOLMA2013\_Jdp2\_DBD,  JOLMA2013\_JDP2\_DBD,  JOLMA2013\_NFE2\_DBD,  JASPAR2014\_JUN::FOS,  JASPAR2014\_FOS |
| M78 | O78 | CCTGCCTGCCT | 0 |  |
| M79 | O79 | CCCAGCCCAGCC | 0 |  |
| M80 | O80 | TTAAAAAAGAAA | 0 |  |
| M81 | O81 | TTTTTTTAAAA | 0 |  |
| M82 | O82 | TTTAAAAATAAA | 0 |  |
| M83 | O83 | TTATTTTTTAAAAG | 0 |  |
| M84 | O84 | CACAGGGCCAGGG | 0 |  |
| M85 | O85 | CAGGTGGCCTCAG | 0 |  |
| M86 | O86 | CCCTGGGCCCAG | 0 |  |
| M87 | O87 | AATGACTCATCAT | 8 | JOLMA2013\_NFE2\_DBD,  JOLMA2013\_JDP2\_full,  JOLMA2013\_Jdp2\_DBD,  JOLMA2013\_JDP2\_DBD,  JASPAR2014\_JUND,  JASPAR2014\_Nfe2l2,  JASPAR2014\_FOS,  JASPAR2014\_FOSL1 |
| M88 | O88 | CAGCTGCCCTCCCC | 0 |  |
| M89 | O89 | GTTTCCATGGCAAC | 11 | JASPAR2014\_Rfx1,  JASPAR2014\_RFX2,  JOLMA2013\_RFX3\_DBD,  JOLMA2013\_RFX2\_DBD,  JOLMA2013\_RFX5\_DBD\_2,  JOLMA2013\_Rfx3\_DBD,  JOLMA2013\_Rfx2\_DBD,  JOLMA2013\_RFX5\_DBD,  JOLMA2013\_RFX4\_DBD,  JOLMA2013\_NFATC1\_full\_3 |
| M90 | O90 | CCAGCCAGGCCCTG | 0 |  |
| M91 | O91 | AAAATGACTCATTT | 10 | JOLMA2013\_JDP2\_DBD,  JOLMA2013\_JDP2\_full,  JOLMA2013\_Jdp2\_DBD,  JASPAR2014\_FOS,  JASPAR2014\_BATF::JUN,  JASPAR2014\_JUNB,  JASPAR2014\_JUND,  JASPAR2014\_FOSL2,  JOLMA2013\_NFE2\_DBD,  JASPAR2014\_JUN::FOS |
| M92 | O92 | TGTTTTTTAAAAAAT | 0 |  |
| M93 | O93 | CTCAGGAGCCAGCAG | 0 |  |
| M94 | O94 | CCAGGAGCCTGCCTG | 0 |  |
| M95 | O95 | TTTTTTAAAAAAAAAA | 0 |  |
| M96 | O96 | GCAGGGCAGGGGCTGGG | 1 | JASPAR2014\_SP1 |
| M97 | O97 | ATAAATATTTGTTGAGTC | 0 |  |
| M98 | O98 | AAATAATTTCTTTTTT | 0 |  |
| M99 | O99 | CAGGAGACAGGCCTGGC | 0 |  |
| M100 | O100 | GGCAGGGGCTCCTGGGG | 0 |  |
| M101 | O101 | TTTTTTTTGTTTTTTTAAA | 0 |  |
| M102 | O102 | TTATTTTTATTTTTAAAA | 0 |  |
| M103 | O103 | CTTCCCAGGAAGGGCAGG | 0 |  |
| M104 | O104 | TTAAATCCATATATTTTAA | 0 |  |
| M106 | O106 | TTTTTTTCCTTTTTAAAGAA | 0 |  |
| M108 | O108 | TTTTTTTCTTTTTAAAAAA | 0 |  |
| M110 | O110 | AAAAAGGTTTTTAAAAAATA | 0 |  |
| M112 | O112 | TTTTTTAAAA | 0 |  |
| M113 | O113 | AAATAAATAA | 1 | JOLMA2013\_FOXC1\_DBD |
| M114 | O114 | GCCTGGGGCT | 6 | JASPAR2014\_TFAP2C,  JOLMA2013\_TFAP2C\_full\_3,  JOLMA2013\_TFAP2B\_DBD\_2,  JOLMA2013\_Tcfap2a\_DBD\_2,  JOLMA2013\_TFAP2A\_DBD\_2,  JOLMA2013\_TFAP2C\_DBD\_2 |
| M115 | O115 | TATTTTTAAA | 0 |  |
| M116 | O116 | ATTTTTAAAA | 0 |  |
| M117 | O117 | TTAAAAATAA | 0 |  |
| M118 | O118 | AGGCAGCTGC | 1 | JASPAR2014\_NHLH1 |
| M119 | O119 | TTTTTTAAAA | 0 |  |
| M120 | O120 | ATTTTTTAAA | 0 |  |
| M121 | O121 | TTTTTTAAAA | 0 |  |
| M122 | O122 | GAGTCACAGC | 1 | JASPAR2014\_Meis1 |
| M123 | O123 | GCTGAGCTCA | 0 |  |
| M124 | O124 | CAGGAAGCTG | 0 |  |
| M125 | O125 | ACAGATTTCA | 0 |  |
| M126 | O126 | AGCAGCCCCAG | 0 |  |
| M127 | O127 | TTAATGTTTAA | 4 | JASPAR2014\_HNF1B,  JOLMA2013\_HNF1A\_full,  JOLMA2013\_HNF1B\_full,  JOLMA2013\_HNF1B\_full\_2 |
| M128 | O128 | CCTCTGCCCAG | 0 |  |
| M129 | O129 | TCTTTTTAAAA | 0 |  |
| M130 | O130 | AGGTCCCTGGG | 0 |  |
| M131 | O131 | CAGCTGCTGGG | 2 | JASPAR2014\_Tcf3,  JASPAR2014\_Tcf12 |
| M132 | O132 | CTGGCCCCAGC | 0 |  |
| M133 | O133 | AGAGACAGACAG | 1 | JASPAR2014\_SMAD2::SMAD3::SMAD4 |
| M134 | O134 | TTAAAAAATAAA | 0 |  |
| M135 | O135 | CAGCTGCTGCCA | 3 | JASPAR2014\_Tcf12,  JASPAR2014\_Tcf3,  JASPAR2014\_Myog |
| M136 | O136 | CCTGGGCCCAGC | 0 |  |
| M137 | O137 | TTTAAAAACAAA | 0 |  |
| M138 | O138 | CCAGCTGCCTGC | 0 |  |
| M139 | O139 | GCTGCTGAGTCA | 3 | JASPAR2014\_Bach1::Mafk,  JASPAR2014\_NFE2::MAF,  JASPAR2014\_Nfe2l2 |
| M140 | O140 | CTGAGGCCCCTG | 0 |  |
| M141 | O141 | TGTGCACACACA | 0 |  |
| M142 | O142 | CCAGCCCTGCCC | 0 |  |
| M143 | O143 | AAAAATAAATAT | 2 | JOLMA2013\_ONECUT3\_DBD,  JOLMA2013\_ONECUT1\_DBD |
| M144 | O144 | CAGCTCTGCAGA | 0 |  |
| M145 | O145 | ATGAATGAATGA | 0 |  |
| M146 | O146 | GTTGCCTGGCAAC | 5 | JOLMA2013\_RFX4\_DBD\_2,  JOLMA2013\_Rfx2\_DBD\_2,  JOLMA2013\_RFX2\_DBD\_2,  JOLMA2013\_RFX3\_DBD\_2,  JASPAR2014\_RFX5 |
| M147 | O147 | TTTTTAAAAAACA | 0 |  |
| M148 | O148 | CCAGGCCAGCAGC | 0 |  |
| M149 | O149 | AATTTTAAAAACA | 0 |  |
| M150 | O150 | AAAAAATAAATAA | 5 | JOLMA2013\_ONECUT3\_DBD,  JOLMA2013\_ONECUT1\_DBD,  JOLMA2013\_ONECUT2\_DBD,  JOLMA2013\_ONECUT1\_full,  JOLMA2013\_FOXL1\_full\_2 |
| M151 | O151 | CTGGGGCCCCAGC | 0 |  |
| M152 | O152 | TTTTGTTTTCATTT | 3 | JASPAR2014\_FOXP1,  JASPAR2014\_FOXP2,  JOLMA2013\_FOXC2\_DBD\_3 |
| M153 | O153 | AAATGAATCACTGT | 0 |  |
| M154 | O154 | AATAAATAAATAAA | 3 | JOLMA2013\_ONECUT3\_DBD,  JOLMA2013\_ONECUT1\_DBD,  JOLMA2013\_FOXL1\_full\_2 |
| M155 | O155 | CTGACTCACTCTGA | 1 | JASPAR2014\_Nfe2l2 |
| M156 | O156 | AAATATTTATTTAA | 0 |  |
| M157 | O157 | TTTAAAAATAAAAA | 0 |  |
| M158 | O158 | GGGCCTGGGTCCCA | 0 |  |
| M159 | O159 | TTTTTTAAAAAAAT | 0 |  |
| M160 | O160 | GGTTAATGATTAAC | 5 | JOLMA2013\_HNF1B\_full,  JOLMA2013\_HNF1B\_full\_2,  JASPAR2014\_HNF1A,  JOLMA2013\_HNF1A\_full,  JASPAR2014\_HNF1B |
| M162 | O162 | GGCCCTGCCTGCCC | 0 |  |
| M164 | O164 | ATTATTTACTTTTAA | 1 | JASPAR2014\_FOXF2 |
| M165 | O165 | CAGGGCAGCTCTGCC | 0 |  |
| M166 | O166 | AAAATCACTGAGTCA | 4 | JASPAR2014\_MAFF,  JASPAR2014\_FOS,  JASPAR2014\_NFE2::MAF,  JASPAR2014\_JUN::FOS |
| M167 | O167 | TTAAAAAATGAAAAA | 0 |  |
| M168 | O168 | AAATAAATAAATAAA | 3 | JOLMA2013\_FOXC1\_DBD,  JOLMA2013\_FOXL1\_full\_2,  JOLMA2013\_ONECUT3\_DBD |
| M169 | O169 | CACATGTGCACACAT | 0 |  |
| M170 | O170 | AGGTGGAGCTCTCCT | 0 |  |
| M171 | O171 | CTGTGTCCACAGAAA | 0 |  |
| M172 | O172 | ACTTTTAAAAAATAA | 0 |  |
| M173 | O173 | ACACAGGAAGCTGTG | 0 |  |
| M174 | O174 | CCAGGCCCAGCCAGG | 0 |  |
| M175 | O175 | CCTGCTCCCCAGGAG | 0 |  |
| M176 | O176 | ACAGTGGTGCCCTGG | 0 |  |
| M177 | O177 | CAGCCCCGGCCCTGG | 1 | JASPAR2014\_KLF5 |
| M178 | O178 | TTTCTTTTTTAAAAAA | 0 |  |
| M179 | O179 | AAATAAATAAATAAAT | 2 | JOLMA2013\_FOXC1\_DBD,  JOLMA2013\_ONECUT3\_DBD |
| M180 | O180 | TTTTTCTTTTCTTTTT | 1 | JASPAR2014\_FOXP1 |
| M181 | O181 | TAAAATGATTACATTT | 0 |  |
| M182 | O182 | CCAGCCCTGCCCCAGC | 0 |  |
| M183 | O183 | AAAAACAAAATAAAAC | 0 |  |
| M184 | O184 | AAAACAGATGTTTTCT | 1 | JOLMA2013\_TFAP4\_full |
| M185 | O185 | CCCAGCCCCGCCCCTG | 5 | JASPAR2014\_KLF5,  JASPAR2014\_SP1,  JASPAR2014\_Klf4,  JASPAR2014\_SP2,  JASPAR2014\_EGR1 |
| M186 | O186 | CAGCTGGCAGGCAGAGC | 0 |  |
| M187 | O187 | TCATTTTTTAAAAGAAT | 0 |  |
| M188 | O188 | AAATATTTGTTTATTGC | 0 |  |
| M189 | O189 | CCAGGCAGGGCAGGGGG | 1 | JASPAR2014\_EGR1 |
| M190 | O190 | TACATTATTTTTAAAAA | 0 |  |
| M191 | O191 | ATTAAATGAAAAATTAA | 0 |  |
| M193 | O193 | CTGAGCCCAGGTGAGTC | 0 |  |
| M194 | O194 | CCAGAGAGCACCCAGGA | 0 |  |
| M195 | O195 | AGGGCTGGGGCTGCAGC | 0 |  |
| M196 | O196 | GGGCAGCTGGGGCCAGG | 0 |  |
| M197 | O197 | ATTTTTTTAAAAAGAAA | 0 |  |
| M200 | O200 | CCCACCCACTTGCTGGGA | 0 |  |
| M201 | O201 | CCTGCCCCGGCCCCAGCC | 4 | JASPAR2014\_EGR1,  JASPAR2014\_SP2,  JASPAR2014\_SP1,  JOLMA2013\_KLF16\_DBD |
| M202 | O202 | AAAAAAAAAACAGCTGTG | 0 |  |
| M204 | O204 | CTTTTGTTTTTAAAAAGA | 0 |  |
| M205 | O205 | TTTCTTTTTTTAAAAAAA | 0 |  |
| M206 | O206 | TAAAATGTCTTTTTTAAA | 0 |  |
| M207 | O207 | CTGCCCTGCCAGCCCCAG | 0 |  |
| M208 | O208 | TTTCTTAAAAAAGGAAAAA | 0 |  |
| M212 | O212 | CTATAAAAAACAAAACAAA | 0 |  |
| M213 | O213 | AAATTATTATTTTTATTCT | 0 |  |
| M217 | O217 | TTTTTTTTTAAAAAAATAA | 0 |  |
| M218 | O218 | CAGGCCCCACCCCCAGCTC | 6 | JASPAR2014\_SP1,  JASPAR2014\_KLF5,  JOLMA2013\_SP1\_DBD,  JASPAR2014\_Zfx,  JOLMA2013\_KLF16\_DBD,  JASPAR2014\_EGR1 |
| M219 | O219 | TGCCTCTGAGGACCCAGGC | 0 |  |
| M220 | O220 | TTTTTTAAAATTTTTTAAA | 0 |  |
| M221 | O221 | ACACAGACAGAGAGACACA | 0 |  |
| M222 | O222 | AAAAATTTTTTAAAATGAA | 0 |  |
| M225 | O225 | ATTTAATTAAAATGTAAAAT | 2 | JOLMA2013\_LMX1A\_DBD,  JOLMA2013\_LMX1B\_DBD |
| M226 | O226 | CTCTCTGCCCTTCCTCCTGC | 1 | JASPAR2014\_ZNF263 |
| M228 | O228 | ATACATTGTTTTAGAAAATA | 0 |  |
| M229 | O229 | GCAGCAGCGCAAGGGCCAGG | 0 |  |
| M230 | O230 | CCCCACCCCCAGGCCTGGCC | 1 | JASPAR2014\_EGR1 |
| M231 | O231 | AAAAAAAAAAAAAAAAAAAA | 0 |  |
| M232 | O232 | CTCTGGGCCCCTGGGAGCAG | 1 | JASPAR2014\_EBF1 |
| M233 | O233 | GAGGAGGCTGGGAGGCTGGG | 2 | JASPAR2014\_ZNF263,  JASPAR2014\_EWSR1-FLI1 |
| M234 | O234 | GCAGGGAGCCCCCGCCCAGG | 0 |  |
| M235 | O235 | GCCTGGCCCCAGGCCACAGC | 5 | JASPAR2014\_TFAP2C,  JOLMA2013\_Tcfap2a\_DBD\_3,  JOLMA2013\_TFAP2C\_full\_2,  JASPAR2014\_TFAP2A,  JOLMA2013\_TFAP2C\_DBD\_3 |
| M238 | O238 | TATTTTTTTTTTTTTAAAAA | 0 |  |

|  |  |
| --- | --- |
| Target Databases | Previous Next Top |

| Database | Number of Motifs | Motifs Matched |
| --- | --- | --- |
| JASPAR\_JOLMA.meme | 940 | 93 |

|  |  |
| --- | --- |
| Matches to Query: M1 (O1) | Previous Next Top |

| Summary | Alignment |
| | Name | JASPAR2014\_FOSL1 | | Database | JASPAR\_JOLMA.meme | | *p*-value | 1.75888e-08 | | *E*-value | 1.65335e-05 | | *q*-value | 3.25084e-05 | | Overlap | 11 | | Offset | -1 | | Orientation | Normal | |  |
| Create custom LOGO ↧ | [Next Match] [Query Top] |
| Summary | Alignment |
| | Name | JASPAR2014\_JUND | | Database | JASPAR\_JOLMA.meme | | *p*-value | 3.23764e-07 | | *E*-value | 0.000304338 | | *q*-value | 0.000299197 | | Overlap | 11 | | Offset | -1 | | Orientation | Normal | |  |
| Create custom LOGO ↧ | [Previous Match] [Next Match] [Query Top] |
| Summary | Alignment |
| | Name | JASPAR2014\_NFE2::MAF | | Database | JASPAR\_JOLMA.meme | | *p*-value | 1.98536e-05 | | *E*-value | 0.0186624 | | *q*-value | 0.00906016 | | Overlap | 11 | | Offset | -2 | | Orientation | Normal | |  |
| Create custom LOGO ↧ | [Previous Match] [Next Match] [Query Top] |
| Summary | Alignment |
| | Name | JASPAR2014\_FOS | | Database | JASPAR\_JOLMA.meme | | *p*-value | 2.82069e-05 | | *E*-value | 0.0265145 | | *q*-value | 0.00906016 | | Overlap | 11 | | Offset | -1 | | Orientation | Normal | |  |
| Create custom LOGO ↧ | [Previous Match] [Next Match] [Query Top] |
| Summary | Alignment |
| | Name | JASPAR2014\_Bach1::Mafk | | Database | JASPAR\_JOLMA.meme | | *p*-value | 3.0959e-05 | | *E*-value | 0.0291015 | | *q*-value | 0.00906016 | | Overlap | 13 | | Offset | 1 | | Orientation | Normal | |  |
| Create custom LOGO ↧ | [Previous Match] [Next Match] [Query Top] |
| Summary | Alignment |
| | Name | JASPAR2014\_JUN::FOS | | Database | JASPAR\_JOLMA.meme | | *p*-value | 3.40488e-05 | | *E*-value | 0.0320058 | | *q*-value | 0.00906016 | | Overlap | 7 | | Offset | -3 | | Orientation | Normal | |  |
| Create custom LOGO ↧ | [Previous Match] [Next Match] [Query Top] |
| Summary | Alignment |
| | Name | JASPAR2014\_JUNB | | Database | JASPAR\_JOLMA.meme | | *p*-value | 3.43143e-05 | | *E*-value | 0.0322555 | | *q*-value | 0.00906016 | | Overlap | 11 | | Offset | 0 | | Orientation | Normal | |  |
| Create custom LOGO ↧ | [Previous Match] [Next Match] [Query Top] |
| Summary | Alignment |
| | Name | JASPAR2014\_Nfe2l2 | | Database | JASPAR\_JOLMA.meme | | *p*-value | 4.01868e-05 | | *E*-value | 0.0377756 | | *q*-value | 0.00928437 | | Overlap | 13 | | Offset | 2 | | Orientation | Normal | |  |
| Create custom LOGO ↧ | [Previous Match] [Next Match] [Query Top] |
| Summary | Alignment |
| | Name | JOLMA2013\_MAFK\_full\_2 | | Database | JASPAR\_JOLMA.meme | | *p*-value | 7.95203e-05 | | *E*-value | 0.0747491 | | *q*-value | 0.0157653 | | Overlap | 13 | | Offset | 1 | | Orientation | Reverse Complement | |  |
| Create custom LOGO ↧ | [Previous Match] [Next Match] [Query Top] |
| Summary | Alignment |
| | Name | JASPAR2014\_FOSL2 | | Database | JASPAR\_JOLMA.meme | | *p*-value | 8.52989e-05 | | *E*-value | 0.080181 | | *q*-value | 0.0157653 | | Overlap | 11 | | Offset | 0 | | Orientation | Normal | |  |
| Create custom LOGO ↧ | [Previous Match] [Query Top] |

|  |  |
| --- | --- |
| Matches to Query: M2 (O2) | Previous Next Top |

| Summary | Alignment |
| | Name | JASPAR2014\_JUND | | Database | JASPAR\_JOLMA.meme | | *p*-value | 1.84615e-07 | | *E*-value | 0.000173538 | | *q*-value | 0.000341398 | | Overlap | 11 | | Offset | -1 | | Orientation | Reverse Complement | |  |
| Create custom LOGO ↧ | [Next Match] [Query Top] |
| Summary | Alignment |
| | Name | JASPAR2014\_FOS | | Database | JASPAR\_JOLMA.meme | | *p*-value | 4.09047e-07 | | *E*-value | 0.000384504 | | *q*-value | 0.0003565 | | Overlap | 11 | | Offset | -1 | | Orientation | Reverse Complement | |  |
| Create custom LOGO ↧ | [Previous Match] [Next Match] [Query Top] |
| Summary | Alignment |
| | Name | JASPAR2014\_FOSL1 | | Database | JASPAR\_JOLMA.meme | | *p*-value | 5.78343e-07 | | *E*-value | 0.000543642 | | *q*-value | 0.0003565 | | Overlap | 11 | | Offset | -1 | | Orientation | Reverse Complement | |  |
| Create custom LOGO ↧ | [Previous Match] [Next Match] [Query Top] |
| Summary | Alignment |
| | Name | JASPAR2014\_FOSL2 | | Database | JASPAR\_JOLMA.meme | | *p*-value | 1.04214e-05 | | *E*-value | 0.00979612 | | *q*-value | 0.00481793 | | Overlap | 11 | | Offset | 0 | | Orientation | Normal | |  |
| Create custom LOGO ↧ | [Previous Match] [Next Match] [Query Top] |
| Summary | Alignment |
| | Name | JASPAR2014\_Meis1 | | Database | JASPAR\_JOLMA.meme | | *p*-value | 3.15637e-05 | | *E*-value | 0.0296698 | | *q*-value | 0.00994737 | | Overlap | 13 | | Offset | 0 | | Orientation | Reverse Complement | |  |
| Create custom LOGO ↧ | [Previous Match] [Next Match] [Query Top] |
| Summary | Alignment |
| | Name | JASPAR2014\_JUNB | | Database | JASPAR\_JOLMA.meme | | *p*-value | 3.51211e-05 | | *E*-value | 0.0330138 | | *q*-value | 0.00994737 | | Overlap | 11 | | Offset | 0 | | Orientation | Normal | |  |
| Create custom LOGO ↧ | [Previous Match] [Next Match] [Query Top] |
| Summary | Alignment |
| | Name | JOLMA2013\_MAFK\_full\_2 | | Database | JASPAR\_JOLMA.meme | | *p*-value | 3.7654e-05 | | *E*-value | 0.0353948 | | *q*-value | 0.00994737 | | Overlap | 13 | | Offset | 1 | | Orientation | Normal | |  |
| Create custom LOGO ↧ | [Previous Match] [Next Match] [Query Top] |
| Summary | Alignment |
| | Name | JASPAR2014\_JUN::FOS | | Database | JASPAR\_JOLMA.meme | | *p*-value | 8.39392e-05 | | *E*-value | 0.0789028 | | *q*-value | 0.0192797 | | Overlap | 7 | | Offset | -3 | | Orientation | Reverse Complement | |  |
| Create custom LOGO ↧ | [Previous Match] [Next Match] [Query Top] |
| Summary | Alignment |
| | Name | JASPAR2014\_NFE2::MAF | | Database | JASPAR\_JOLMA.meme | | *p*-value | 0.000103055 | | *E*-value | 0.0968719 | | *q*-value | 0.0192797 | | Overlap | 11 | | Offset | 4 | | Orientation | Reverse Complement | |  |
| Create custom LOGO ↧ | [Previous Match] [Query Top] |

|  |  |
| --- | --- |
| Matches to Query: M3 (O3) | Previous Next Top |

| Summary | Alignment |
| | Name | JOLMA2013\_JDP2\_full | | Database | JASPAR\_JOLMA.meme | | *p*-value | 3.56789e-10 | | *E*-value | 3.35382e-07 | | *q*-value | 6.65529e-07 | | Overlap | 9 | | Offset | -3 | | Orientation | Normal | |  |
| Create custom LOGO ↧ | [Next Match] [Query Top] |
| Summary | Alignment |
| | Name | JOLMA2013\_JDP2\_DBD | | Database | JASPAR\_JOLMA.meme | | *p*-value | 3.75583e-09 | | *E*-value | 3.53048e-06 | | *q*-value | 2.33528e-06 | | Overlap | 9 | | Offset | -3 | | Orientation | Normal | |  |
| Create custom LOGO ↧ | [Previous Match] [Next Match] [Query Top] |
| Summary | Alignment |
| | Name | JOLMA2013\_Jdp2\_DBD | | Database | JASPAR\_JOLMA.meme | | *p*-value | 3.75583e-09 | | *E*-value | 3.53048e-06 | | *q*-value | 2.33528e-06 | | Overlap | 9 | | Offset | -3 | | Orientation | Normal | |  |
| Create custom LOGO ↧ | [Previous Match] [Next Match] [Query Top] |
| Summary | Alignment |
| | Name | JASPAR2014\_BATF::JUN | | Database | JASPAR\_JOLMA.meme | | *p*-value | 8.23419e-06 | | *E*-value | 0.00774014 | | *q*-value | 0.00213625 | | Overlap | 10 | | Offset | -4 | | Orientation | Reverse Complement | |  |
| Create custom LOGO ↧ | [Previous Match] [Next Match] [Query Top] |
| Summary | Alignment |
| | Name | JASPAR2014\_JUNB | | Database | JASPAR\_JOLMA.meme | | *p*-value | 1.26532e-05 | | *E*-value | 0.011894 | | *q*-value | 0.00262248 | | Overlap | 11 | | Offset | -1 | | Orientation | Normal | |  |
| Create custom LOGO ↧ | [Previous Match] [Next Match] [Query Top] |
| Summary | Alignment |
| | Name | JASPAR2014\_FOS | | Database | JASPAR\_JOLMA.meme | | *p*-value | 3.20526e-05 | | *E*-value | 0.0301294 | | *q*-value | 0.00543532 | | Overlap | 11 | | Offset | -2 | | Orientation | Normal | |  |
| Create custom LOGO ↧ | [Previous Match] [Next Match] [Query Top] |
| Summary | Alignment |
| | Name | JOLMA2013\_NFE2\_DBD | | Database | JASPAR\_JOLMA.meme | | *p*-value | 3.20526e-05 | | *E*-value | 0.0301294 | | *q*-value | 0.00543532 | | Overlap | 11 | | Offset | -2 | | Orientation | Normal | |  |
| Create custom LOGO ↧ | [Previous Match] [Next Match] [Query Top] |
| Summary | Alignment |
| | Name | JASPAR2014\_FOSL2 | | Database | JASPAR\_JOLMA.meme | | *p*-value | 3.90273e-05 | | *E*-value | 0.0366856 | | *q*-value | 0.00559989 | | Overlap | 11 | | Offset | -1 | | Orientation | Normal | |  |
| Create custom LOGO ↧ | [Previous Match] [Next Match] [Query Top] |
| Summary | Alignment |
| | Name | JASPAR2014\_JUN::FOS | | Database | JASPAR\_JOLMA.meme | | *p*-value | 4.47675e-05 | | *E*-value | 0.0420814 | | *q*-value | 0.00596471 | | Overlap | 7 | | Offset | -4 | | Orientation | Normal | |  |
| Create custom LOGO ↧ | [Previous Match] [Next Match] [Query Top] |
| Summary | Alignment |
| | Name | JASPAR2014\_JUND | | Database | JASPAR\_JOLMA.meme | | *p*-value | 5.20621e-05 | | *E*-value | 0.0489384 | | *q*-value | 0.00647419 | | Overlap | 11 | | Offset | -2 | | Orientation | Normal | |  |
| Create custom LOGO ↧ | [Previous Match] [Query Top] |

|  |  |
| --- | --- |
| Matches to Query: M4 (O4) | Previous Next Top |

|  |  |
| --- | --- |
| Matches to Query: M5 (O5) | Previous Next Top |

| Summary | Alignment |
| | Name | JASPAR2014\_TFAP2C | | Database | JASPAR\_JOLMA.meme | | *p*-value | 3.79102e-05 | | *E*-value | 0.0356356 | | *q*-value | 0.0702843 | | Overlap | 12 | | Offset | 3 | | Orientation | Normal | |  |
| Create custom LOGO ↧ | [Next Match] [Query Top] |
| Summary | Alignment |
| | Name | JASPAR2014\_SP1 | | Database | JASPAR\_JOLMA.meme | | *p*-value | 9.41507e-05 | | *E*-value | 0.0885017 | | *q*-value | 0.0872762 | | Overlap | 11 | | Offset | -1 | | Orientation | Normal | |  |
| Create custom LOGO ↧ | [Previous Match] [Query Top] |

|  |  |
| --- | --- |
| Matches to Query: M6 (O6) | Previous Next Top |

|  |  |
| --- | --- |
| Matches to Query: M7 (O7) | Previous Next Top |

| Summary | Alignment |
| | Name | JOLMA2013\_FOXL1\_full\_2 | | Database | JASPAR\_JOLMA.meme | | *p*-value | 4.94116e-06 | | *E*-value | 0.00464469 | | *q*-value | 0.0091636 | | Overlap | 13 | | Offset | 0 | | Orientation | Normal | |  |
| Create custom LOGO ↧ | [Next Match] [Query Top] |
| Summary | Alignment |
| | Name | JOLMA2013\_FOXC1\_DBD | | Database | JASPAR\_JOLMA.meme | | *p*-value | 1.74683e-05 | | *E*-value | 0.0164202 | | *q*-value | 0.0161979 | | Overlap | 12 | | Offset | 1 | | Orientation | Normal | |  |
| Create custom LOGO ↧ | [Previous Match] [Next Match] [Query Top] |
| Summary | Alignment |
| | Name | JOLMA2013\_FOXC2\_DBD\_2 | | Database | JASPAR\_JOLMA.meme | | *p*-value | 3.5493e-05 | | *E*-value | 0.0333634 | | *q*-value | 0.0181696 | | Overlap | 11 | | Offset | -1 | | Orientation | Normal | |  |
| Create custom LOGO ↧ | [Previous Match] [Next Match] [Query Top] |
| Summary | Alignment |
| | Name | JOLMA2013\_ONECUT3\_DBD | | Database | JASPAR\_JOLMA.meme | | *p*-value | 3.91893e-05 | | *E*-value | 0.036838 | | *q*-value | 0.0181696 | | Overlap | 13 | | Offset | 0 | | Orientation | Normal | |  |
| Create custom LOGO ↧ | [Previous Match] [Next Match] [Query Top] |
| Summary | Alignment |
| | Name | JOLMA2013\_ONECUT1\_DBD | | Database | JASPAR\_JOLMA.meme | | *p*-value | 0.000103901 | | *E*-value | 0.0976669 | | *q*-value | 0.0351484 | | Overlap | 13 | | Offset | 0 | | Orientation | Normal | |  |
| Create custom LOGO ↧ | [Previous Match] [Query Top] |

|  |  |
| --- | --- |
| Matches to Query: M8 (O8) | Previous Next Top |

| Summary | Alignment |
| | Name | JOLMA2013\_FOXD2\_DBD | | Database | JASPAR\_JOLMA.meme | | *p*-value | 1.06568e-05 | | *E*-value | 0.0100174 | | *q*-value | 0.0180473 | | Overlap | 14 | | Offset | 0 | | Orientation | Normal | |  |
| Create custom LOGO ↧ | [Next Match] [Query Top] |
| Summary | Alignment |
| | Name | JOLMA2013\_FOXD3\_DBD | | Database | JASPAR\_JOLMA.meme | | *p*-value | 3.54937e-05 | | *E*-value | 0.0333641 | | *q*-value | 0.0180473 | | Overlap | 14 | | Offset | 0 | | Orientation | Normal | |  |
| Create custom LOGO ↧ | [Previous Match] [Next Match] [Query Top] |
| Summary | Alignment |
| | Name | JOLMA2013\_FOXC1\_DBD\_2 | | Database | JASPAR\_JOLMA.meme | | *p*-value | 0.000104296 | | *E*-value | 0.0980382 | | *q*-value | 0.0391817 | | Overlap | 14 | | Offset | 0 | | Orientation | Reverse Complement | |  |
| Create custom LOGO ↧ | [Previous Match] [Query Top] |

|  |  |
| --- | --- |
| Matches to Query: M9 (O9) | Previous Next Top |

|  |  |
| --- | --- |
| Matches to Query: M10 (O10) | Previous Next Top |

|  |  |
| --- | --- |
| Matches to Query: M11 (O11) | Previous Next Top |

| Summary | Alignment |
| | Name | JOLMA2013\_FOXC1\_DBD | | Database | JASPAR\_JOLMA.meme | | *p*-value | 1.61504e-05 | | *E*-value | 0.0151813 | | *q*-value | 0.0296743 | | Overlap | 13 | | Offset | -1 | | Orientation | Normal | |  |
| Create custom LOGO ↧ | [Query Top] |

|  |  |
| --- | --- |
| Matches to Query: M12 (O12) | Previous Next Top |

| Summary | Alignment |
| | Name | JASPAR2014\_ZNF263 | | Database | JASPAR\_JOLMA.meme | | *p*-value | 2.45904e-07 | | *E*-value | 0.00023115 | | *q*-value | 0.000461905 | | Overlap | 13 | | Offset | 7 | | Orientation | Reverse Complement | |  |
| Create custom LOGO ↧ | [Query Top] |

|  |  |
| --- | --- |
| Matches to Query: M13 (O13) | Previous Next Top |

| Summary | Alignment |
| | Name | JASPAR2014\_KLF5 | | Database | JASPAR\_JOLMA.meme | | *p*-value | 4.13982e-06 | | *E*-value | 0.00389143 | | *q*-value | 0.00770964 | | Overlap | 10 | | Offset | -4 | | Orientation | Normal | |  |
| Create custom LOGO ↧ | [Next Match] [Query Top] |
| Summary | Alignment |
| | Name | JASPAR2014\_E2F3 | | Database | JASPAR\_JOLMA.meme | | *p*-value | 3.19536e-05 | | *E*-value | 0.0300364 | | *q*-value | 0.0287657 | | Overlap | 13 | | Offset | 2 | | Orientation | Normal | |  |
| Create custom LOGO ↧ | [Previous Match] [Next Match] [Query Top] |
| Summary | Alignment |
| | Name | JASPAR2014\_Klf4 | | Database | JASPAR\_JOLMA.meme | | *p*-value | 5.78678e-05 | | *E*-value | 0.0543957 | | *q*-value | 0.0287657 | | Overlap | 10 | | Offset | -4 | | Orientation | Reverse Complement | |  |
| Create custom LOGO ↧ | [Previous Match] [Next Match] [Query Top] |
| Summary | Alignment |
| | Name | JASPAR2014\_SP2 | | Database | JASPAR\_JOLMA.meme | | *p*-value | 6.1785e-05 | | *E*-value | 0.0580779 | | *q*-value | 0.0287657 | | Overlap | 14 | | Offset | 1 | | Orientation | Normal | |  |
| Create custom LOGO ↧ | [Previous Match] [Next Match] [Query Top] |
| Summary | Alignment |
| | Name | JASPAR2014\_RREB1 | | Database | JASPAR\_JOLMA.meme | | *p*-value | 9.75181e-05 | | *E*-value | 0.091667 | | *q*-value | 0.0328618 | | Overlap | 14 | | Offset | 6 | | Orientation | Normal | |  |
| Create custom LOGO ↧ | [Previous Match] [Query Top] |

|  |  |
| --- | --- |
| Matches to Query: M14 (O14) | Previous Next Top |

| Summary | Alignment |
| | Name | JASPAR2014\_HNF1A | | Database | JASPAR\_JOLMA.meme | | *p*-value | 1.20747e-05 | | *E*-value | 0.0113502 | | *q*-value | 0.0214983 | | Overlap | 13 | | Offset | -1 | | Orientation | Reverse Complement | |  |
| Create custom LOGO ↧ | [Next Match] [Query Top] |
| Summary | Alignment |
| | Name | JASPAR2014\_HNF1B | | Database | JASPAR\_JOLMA.meme | | *p*-value | 9.80383e-05 | | *E*-value | 0.092156 | | *q*-value | 0.0581836 | | Overlap | 12 | | Offset | -1 | | Orientation | Reverse Complement | |  |
| Create custom LOGO ↧ | [Previous Match] [Query Top] |

|  |  |
| --- | --- |
| Matches to Query: M15 (O15) | Previous Next Top |

| Summary | Alignment |
| | Name | JASPAR2014\_MEF2A | | Database | JASPAR\_JOLMA.meme | | *p*-value | 9.16398e-09 | | *E*-value | 8.61414e-06 | | *q*-value | 1.72283e-05 | | Overlap | 14 | | Offset | 1 | | Orientation | Reverse Complement | |  |
| Create custom LOGO ↧ | [Next Match] [Query Top] |
| Summary | Alignment |
| | Name | JASPAR2014\_MEF2C | | Database | JASPAR\_JOLMA.meme | | *p*-value | 6.5553e-08 | | *E*-value | 6.16199e-05 | | *q*-value | 6.16199e-05 | | Overlap | 14 | | Offset | 0 | | Orientation | Reverse Complement | |  |
| Create custom LOGO ↧ | [Previous Match] [Next Match] [Query Top] |
| Summary | Alignment |
| | Name | JOLMA2013\_MEF2A\_DBD | | Database | JASPAR\_JOLMA.meme | | *p*-value | 1.62624e-07 | | *E*-value | 0.000152867 | | *q*-value | 0.000101911 | | Overlap | 12 | | Offset | -1 | | Orientation | Reverse Complement | |  |
| Create custom LOGO ↧ | [Previous Match] [Next Match] [Query Top] |
| Summary | Alignment |
| | Name | JOLMA2013\_MEF2D\_DBD | | Database | JASPAR\_JOLMA.meme | | *p*-value | 2.95997e-06 | | *E*-value | 0.00278238 | | *q*-value | 0.00139119 | | Overlap | 12 | | Offset | -1 | | Orientation | Reverse Complement | |  |
| Create custom LOGO ↧ | [Previous Match] [Next Match] [Query Top] |
| Summary | Alignment |
| | Name | JOLMA2013\_MEF2B\_full | | Database | JASPAR\_JOLMA.meme | | *p*-value | 1.52668e-05 | | *E*-value | 0.0143508 | | *q*-value | 0.00574031 | | Overlap | 12 | | Offset | -1 | | Orientation | Reverse Complement | |  |
| Create custom LOGO ↧ | [Previous Match] [Query Top] |

|  |  |
| --- | --- |
| Matches to Query: M16 (O16) | Previous Next Top |

| Summary | Alignment |
| | Name | JASPAR2014\_EGR1 | | Database | JASPAR\_JOLMA.meme | | *p*-value | 4.02745e-06 | | *E*-value | 0.00378581 | | *q*-value | 0.00752466 | | Overlap | 14 | | Offset | 0 | | Orientation | Normal | |  |
| Create custom LOGO ↧ | [Next Match] [Query Top] |
| Summary | Alignment |
| | Name | JASPAR2014\_E2F3 | | Database | JASPAR\_JOLMA.meme | | *p*-value | 9.08936e-05 | | *E*-value | 0.08544 | | *q*-value | 0.0849102 | | Overlap | 14 | | Offset | 0 | | Orientation | Normal | |  |
| Create custom LOGO ↧ | [Previous Match] [Query Top] |

|  |  |
| --- | --- |
| Matches to Query: M17 (O17) | Previous Next Top |

|  |  |
| --- | --- |
| Matches to Query: M18 (O18) | Previous Next Top |

| Summary | Alignment |
| | Name | JASPAR2014\_EWSR1-FLI1 | | Database | JASPAR\_JOLMA.meme | | *p*-value | 8.15026e-06 | | *E*-value | 0.00766124 | | *q*-value | 0.0153094 | | Overlap | 15 | | Offset | 3 | | Orientation | Normal | |  |
| Create custom LOGO ↧ | [Next Match] [Query Top] |
| Summary | Alignment |
| | Name | JASPAR2014\_ZNF263 | | Database | JASPAR\_JOLMA.meme | | *p*-value | 1.7788e-05 | | *E*-value | 0.0167208 | | *q*-value | 0.0167065 | | Overlap | 15 | | Offset | 5 | | Orientation | Normal | |  |
| Create custom LOGO ↧ | [Previous Match] [Next Match] [Query Top] |
| Summary | Alignment |
| | Name | JASPAR2014\_E2F6 | | Database | JASPAR\_JOLMA.meme | | *p*-value | 7.82044e-05 | | *E*-value | 0.0735122 | | *q*-value | 0.0489662 | | Overlap | 11 | | Offset | -1 | | Orientation | Normal | |  |
| Create custom LOGO ↧ | [Previous Match] [Query Top] |

|  |  |
| --- | --- |
| Matches to Query: M19 (O19) | Previous Next Top |

| Summary | Alignment |
| | Name | JASPAR2014\_ZNF263 | | Database | JASPAR\_JOLMA.meme | | *p*-value | 5.34347e-07 | | *E*-value | 0.000502286 | | *q*-value | 0.00100457 | | Overlap | 13 | | Offset | 6 | | Orientation | Reverse Complement | |  |
| Create custom LOGO ↧ | [Query Top] |

|  |  |
| --- | --- |
| Matches to Query: M20 (O20) | Previous Next Top |

|  |  |
| --- | --- |
| Matches to Query: M21 (O21) | Previous Next Top |

|  |  |
| --- | --- |
| Matches to Query: M22 (O22) | Previous Next Top |

| Summary | Alignment |
| | Name | JASPAR2014\_Klf4 | | Database | JASPAR\_JOLMA.meme | | *p*-value | 5.04593e-05 | | *E*-value | 0.0474317 | | *q*-value | 0.0864272 | | Overlap | 10 | | Offset | -3 | | Orientation | Normal | |  |
| Create custom LOGO ↧ | [Query Top] |

|  |  |
| --- | --- |
| Matches to Query: M23 (O23) | Previous Next Top |

|  |  |
| --- | --- |
| Matches to Query: M24 (O24) | Previous Next Top |

|  |  |
| --- | --- |
| Matches to Query: M25 (O25) | Previous Next Top |

|  |  |
| --- | --- |
| Matches to Query: M26 (O26) | Previous Next Top |

|  |  |
| --- | --- |
| Matches to Query: M27 (O27) | Previous Next Top |

|  |  |
| --- | --- |
| Matches to Query: M28 (O28) | Previous Next Top |

|  |  |
| --- | --- |
| Matches to Query: M29 (O29) | Previous Next Top |

|  |  |
| --- | --- |
| Matches to Query: M30 (O30) | Previous Next Top |

| Summary | Alignment |
| | Name | JASPAR2014\_SP2 | | Database | JASPAR\_JOLMA.meme | | *p*-value | 4.0816e-05 | | *E*-value | 0.038367 | | *q*-value | 0.0765043 | | Overlap | 15 | | Offset | 0 | | Orientation | Reverse Complement | |  |
| Create custom LOGO ↧ | [Next Match] [Query Top] |
| Summary | Alignment |
| | Name | JASPAR2014\_SP1 | | Database | JASPAR\_JOLMA.meme | | *p*-value | 9.61971e-05 | | *E*-value | 0.0904252 | | *q*-value | 0.089623 | | Overlap | 11 | | Offset | -4 | | Orientation | Reverse Complement | |  |
| Create custom LOGO ↧ | [Previous Match] [Query Top] |

|  |  |
| --- | --- |
| Matches to Query: M31 (O31) | Previous Next Top |

| Summary | Alignment |
| | Name | JASPAR2014\_RFX2 | | Database | JASPAR\_JOLMA.meme | | *p*-value | 9.29698e-08 | | *E*-value | 8.73916e-05 | | *q*-value | 0.000173606 | | Overlap | 13 | | Offset | 3 | | Orientation | Normal | |  |
| Create custom LOGO ↧ | [Next Match] [Query Top] |
| Summary | Alignment |
| | Name | JASPAR2014\_RFX5 | | Database | JASPAR\_JOLMA.meme | | *p*-value | 6.4891e-06 | | *E*-value | 0.00609976 | | *q*-value | 0.0041268 | | Overlap | 13 | | Offset | 1 | | Orientation | Normal | |  |
| Create custom LOGO ↧ | [Previous Match] [Next Match] [Query Top] |
| Summary | Alignment |
| | Name | JASPAR2014\_Rfx1 | | Database | JASPAR\_JOLMA.meme | | *p*-value | 6.62998e-06 | | *E*-value | 0.00623218 | | *q*-value | 0.0041268 | | Overlap | 11 | | Offset | 3 | | Orientation | Normal | |  |
| Create custom LOGO ↧ | [Previous Match] [Next Match] [Query Top] |
| Summary | Alignment |
| | Name | JOLMA2013\_Rfx2\_DBD | | Database | JASPAR\_JOLMA.meme | | *p*-value | 2.0476e-05 | | *E*-value | 0.0192475 | | *q*-value | 0.00446459 | | Overlap | 12 | | Offset | 4 | | Orientation | Reverse Complement | |  |
| Create custom LOGO ↧ | [Previous Match] [Next Match] [Query Top] |
| Summary | Alignment |
| | Name | JOLMA2013\_RFX2\_DBD | | Database | JASPAR\_JOLMA.meme | | *p*-value | 2.22731e-05 | | *E*-value | 0.0209367 | | *q*-value | 0.00446459 | | Overlap | 12 | | Offset | 4 | | Orientation | Reverse Complement | |  |
| Create custom LOGO ↧ | [Previous Match] [Next Match] [Query Top] |
| Summary | Alignment |
| | Name | JOLMA2013\_RFX3\_DBD | | Database | JASPAR\_JOLMA.meme | | *p*-value | 2.22731e-05 | | *E*-value | 0.0209367 | | *q*-value | 0.00446459 | | Overlap | 12 | | Offset | 4 | | Orientation | Normal | |  |
| Create custom LOGO ↧ | [Previous Match] [Next Match] [Query Top] |
| Summary | Alignment |
| | Name | JOLMA2013\_Rfx3\_DBD | | Database | JASPAR\_JOLMA.meme | | *p*-value | 2.42123e-05 | | *E*-value | 0.0227596 | | *q*-value | 0.00446459 | | Overlap | 12 | | Offset | 4 | | Orientation | Normal | |  |
| Create custom LOGO ↧ | [Previous Match] [Next Match] [Query Top] |
| Summary | Alignment |
| | Name | JOLMA2013\_RFX5\_DBD | | Database | JASPAR\_JOLMA.meme | | *p*-value | 2.62997e-05 | | *E*-value | 0.0247218 | | *q*-value | 0.00446459 | | Overlap | 12 | | Offset | 4 | | Orientation | Reverse Complement | |  |
| Create custom LOGO ↧ | [Previous Match] [Next Match] [Query Top] |
| Summary | Alignment |
| | Name | JOLMA2013\_RFX5\_DBD\_2 | | Database | JASPAR\_JOLMA.meme | | *p*-value | 3.62838e-05 | | *E*-value | 0.0341067 | | *q*-value | 0.00458994 | | Overlap | 12 | | Offset | 4 | | Orientation | Normal | |  |
| Create custom LOGO ↧ | [Previous Match] [Next Match] [Query Top] |
| Summary | Alignment |
| | Name | JOLMA2013\_RFX4\_DBD | | Database | JASPAR\_JOLMA.meme | | *p*-value | 5.32317e-05 | | *E*-value | 0.0500378 | | *q*-value | 0.00584715 | | Overlap | 12 | | Offset | 4 | | Orientation | Reverse Complement | |  |
| Create custom LOGO ↧ | [Previous Match] [Query Top] |

|  |  |
| --- | --- |
| Matches to Query: M32 (O32) | Previous Next Top |

| Summary | Alignment |
| | Name | JOLMA2013\_JDP2\_full | | Database | JASPAR\_JOLMA.meme | | *p*-value | 7.05779e-07 | | *E*-value | 0.000663432 | | *q*-value | 0.000838244 | | Overlap | 9 | | Offset | -1 | | Orientation | Reverse Complement | |  |
| Create custom LOGO ↧ | [Next Match] [Query Top] |
| Summary | Alignment |
| | Name | JOLMA2013\_Jdp2\_DBD | | Database | JASPAR\_JOLMA.meme | | *p*-value | 8.98763e-07 | | *E*-value | 0.000844838 | | *q*-value | 0.000838244 | | Overlap | 9 | | Offset | -1 | | Orientation | Reverse Complement | |  |
| Create custom LOGO ↧ | [Previous Match] [Next Match] [Query Top] |
| Summary | Alignment |
| | Name | JOLMA2013\_JDP2\_DBD | | Database | JASPAR\_JOLMA.meme | | *p*-value | 3.91405e-06 | | *E*-value | 0.0036792 | | *q*-value | 0.00243366 | | Overlap | 9 | | Offset | -1 | | Orientation | Reverse Complement | |  |
| Create custom LOGO ↧ | [Previous Match] [Next Match] [Query Top] |
| Summary | Alignment |
| | Name | JASPAR2014\_FOS | | Database | JASPAR\_JOLMA.meme | | *p*-value | 2.38229e-05 | | *E*-value | 0.0223935 | | *q*-value | 0.00858084 | | Overlap | 11 | | Offset | 0 | | Orientation | Reverse Complement | |  |
| Create custom LOGO ↧ | [Previous Match] [Next Match] [Query Top] |
| Summary | Alignment |
| | Name | JOLMA2013\_NFE2\_DBD | | Database | JASPAR\_JOLMA.meme | | *p*-value | 3.81091e-05 | | *E*-value | 0.0358226 | | *q*-value | 0.00883517 | | Overlap | 11 | | Offset | 0 | | Orientation | Reverse Complement | |  |
| Create custom LOGO ↧ | [Previous Match] [Next Match] [Query Top] |
| Summary | Alignment |
| | Name | JASPAR2014\_JUND | | Database | JASPAR\_JOLMA.meme | | *p*-value | 4.26287e-05 | | *E*-value | 0.040071 | | *q*-value | 0.00883517 | | Overlap | 11 | | Offset | 0 | | Orientation | Reverse Complement | |  |
| Create custom LOGO ↧ | [Previous Match] [Next Match] [Query Top] |
| Summary | Alignment |
| | Name | JASPAR2014\_JUN::FOS | | Database | JASPAR\_JOLMA.meme | | *p*-value | 5.57026e-05 | | *E*-value | 0.0523605 | | *q*-value | 0.0103904 | | Overlap | 7 | | Offset | -2 | | Orientation | Reverse Complement | |  |
| Create custom LOGO ↧ | [Previous Match] [Next Match] [Query Top] |
| Summary | Alignment |
| | Name | JASPAR2014\_JUNB | | Database | JASPAR\_JOLMA.meme | | *p*-value | 9.6116e-05 | | *E*-value | 0.0903491 | | *q*-value | 0.0162989 | | Overlap | 10 | | Offset | -1 | | Orientation | Reverse Complement | |  |
| Create custom LOGO ↧ | [Previous Match] [Query Top] |

|  |  |
| --- | --- |
| Matches to Query: M33 (O33) | Previous Next Top |

|  |  |
| --- | --- |
| Matches to Query: M34 (O34) | Previous Next Top |

| Summary | Alignment |
| | Name | JASPAR2014\_Tcf12 | | Database | JASPAR\_JOLMA.meme | | *p*-value | 4.68425e-05 | | *E*-value | 0.044032 | | *q*-value | 0.0808703 | | Overlap | 11 | | Offset | -1 | | Orientation | Normal | |  |
| Create custom LOGO ↧ | [Query Top] |

|  |  |
| --- | --- |
| Matches to Query: M35 (O35) | Previous Next Top |

|  |  |
| --- | --- |
| Matches to Query: M36 (O36) | Previous Next Top |

|  |  |
| --- | --- |
| Matches to Query: M37 (O37) | Previous Next Top |

|  |  |
| --- | --- |
| Matches to Query: M38 (O38) | Previous Next Top |

|  |  |
| --- | --- |
| Matches to Query: M39 (O39) | Previous Next Top |

|  |  |
| --- | --- |
| Matches to Query: M40 (O40) | Previous Next Top |

|  |  |
| --- | --- |
| Matches to Query: M41 (O41) | Previous Next Top |

| Summary | Alignment |
| | Name | JOLMA2013\_FOXC1\_DBD | | Database | JASPAR\_JOLMA.meme | | *p*-value | 5.32224e-06 | | *E*-value | 0.00500291 | | *q*-value | 0.00986496 | | Overlap | 11 | | Offset | -1 | | Orientation | Normal | |  |
| Create custom LOGO ↧ | [Next Match] [Query Top] |
| Summary | Alignment |
| | Name | JOLMA2013\_FOXC2\_DBD\_3 | | Database | JASPAR\_JOLMA.meme | | *p*-value | 1.70601e-05 | | *E*-value | 0.0160365 | | *q*-value | 0.0158108 | | Overlap | 12 | | Offset | 0 | | Orientation | Normal | |  |
| Create custom LOGO ↧ | [Previous Match] [Next Match] [Query Top] |
| Summary | Alignment |
| | Name | JOLMA2013\_FOXL1\_full\_2 | | Database | JASPAR\_JOLMA.meme | | *p*-value | 3.94742e-05 | | *E*-value | 0.0371058 | | *q*-value | 0.0243889 | | Overlap | 11 | | Offset | 2 | | Orientation | Normal | |  |
| Create custom LOGO ↧ | [Previous Match] [Next Match] [Query Top] |
| Summary | Alignment |
| | Name | JASPAR2014\_FOXP1 | | Database | JASPAR\_JOLMA.meme | | *p*-value | 8.31732e-05 | | *E*-value | 0.0781828 | | *q*-value | 0.0385411 | | Overlap | 12 | | Offset | 2 | | Orientation | Normal | |  |
| Create custom LOGO ↧ | [Previous Match] [Query Top] |

|  |  |
| --- | --- |
| Matches to Query: M42 (O42) | Previous Next Top |

|  |  |
| --- | --- |
| Matches to Query: M43 (O43) | Previous Next Top |

|  |  |
| --- | --- |
| Matches to Query: M44 (O44) | Previous Next Top |

| Summary | Alignment |
| | Name | JASPAR2014\_FOXP1 | | Database | JASPAR\_JOLMA.meme | | *p*-value | 5.10837e-05 | | *E*-value | 0.0480187 | | *q*-value | 0.0873933 | | Overlap | 13 | | Offset | 1 | | Orientation | Normal | |  |
| Create custom LOGO ↧ | [Next Match] [Query Top] |
| Summary | Alignment |
| | Name | JASPAR2014\_Foxd3 | | Database | JASPAR\_JOLMA.meme | | *p*-value | 9.36265e-05 | | *E*-value | 0.0880089 | | *q*-value | 0.0873933 | | Overlap | 11 | | Offset | -2 | | Orientation | Reverse Complement | |  |
| Create custom LOGO ↧ | [Previous Match] [Query Top] |

|  |  |
| --- | --- |
| Matches to Query: M45 (O45) | Previous Next Top |

| Summary | Alignment |
| | Name | JOLMA2013\_FOXC1\_DBD | | Database | JASPAR\_JOLMA.meme | | *p*-value | 6.41874e-06 | | *E*-value | 0.00603361 | | *q*-value | 0.0118354 | | Overlap | 13 | | Offset | -3 | | Orientation | Normal | |  |
| Create custom LOGO ↧ | [Query Top] |

|  |  |
| --- | --- |
| Matches to Query: M46 (O46) | Previous Next Top |

|  |  |
| --- | --- |
| Matches to Query: M47 (O47) | Previous Next Top |

|  |  |
| --- | --- |
| Matches to Query: M50 (O50) | Previous Next Top |

|  |  |
| --- | --- |
| Matches to Query: M52 (O52) | Previous Next Top |

|  |  |
| --- | --- |
| Matches to Query: M53 (O53) | Previous Next Top |

| Summary | Alignment |
| | Name | JOLMA2013\_ONECUT3\_DBD | | Database | JASPAR\_JOLMA.meme | | *p*-value | 5.32472e-05 | | *E*-value | 0.0500524 | | *q*-value | 0.0909741 | | Overlap | 14 | | Offset | -2 | | Orientation | Normal | |  |
| Create custom LOGO ↧ | [Next Match] [Query Top] |
| Summary | Alignment |
| | Name | JOLMA2013\_ONECUT1\_DBD | | Database | JASPAR\_JOLMA.meme | | *p*-value | 9.69675e-05 | | *E*-value | 0.0911495 | | *q*-value | 0.0909741 | | Overlap | 14 | | Offset | -2 | | Orientation | Normal | |  |
| Create custom LOGO ↧ | [Previous Match] [Query Top] |

|  |  |
| --- | --- |
| Matches to Query: M54 (O54) | Previous Next Top |

| Summary | Alignment |
| | Name | JOLMA2013\_FOXD2\_DBD | | Database | JASPAR\_JOLMA.meme | | *p*-value | 8.528e-06 | | *E*-value | 0.00801632 | | *q*-value | 0.0111215 | | Overlap | 10 | | Offset | 4 | | Orientation | Normal | |  |
| Create custom LOGO ↧ | [Next Match] [Query Top] |
| Summary | Alignment |
| | Name | JOLMA2013\_FOXB1\_DBD\_2 | | Database | JASPAR\_JOLMA.meme | | *p*-value | 1.201e-05 | | *E*-value | 0.0112894 | | *q*-value | 0.0111215 | | Overlap | 12 | | Offset | 6 | | Orientation | Reverse Complement | |  |
| Create custom LOGO ↧ | [Previous Match] [Next Match] [Query Top] |
| Summary | Alignment |
| | Name | JOLMA2013\_FOXD3\_DBD | | Database | JASPAR\_JOLMA.meme | | *p*-value | 1.9181e-05 | | *E*-value | 0.0180302 | | *q*-value | 0.0118413 | | Overlap | 10 | | Offset | 4 | | Orientation | Normal | |  |
| Create custom LOGO ↧ | [Previous Match] [Next Match] [Query Top] |
| Summary | Alignment |
| | Name | JOLMA2013\_FOXC1\_DBD\_2 | | Database | JASPAR\_JOLMA.meme | | *p*-value | 2.89596e-05 | | *E*-value | 0.027222 | | *q*-value | 0.0134086 | | Overlap | 10 | | Offset | 4 | | Orientation | Reverse Complement | |  |
| Create custom LOGO ↧ | [Previous Match] [Next Match] [Query Top] |
| Summary | Alignment |
| | Name | JOLMA2013\_FOXC2\_DBD | | Database | JASPAR\_JOLMA.meme | | *p*-value | 5.61171e-05 | | *E*-value | 0.0527501 | | *q*-value | 0.0207862 | | Overlap | 10 | | Offset | 4 | | Orientation | Reverse Complement | |  |
| Create custom LOGO ↧ | [Previous Match] [Query Top] |

|  |  |
| --- | --- |
| Matches to Query: M55 (O55) | Previous Next Top |

| Summary | Alignment |
| | Name | JASPAR2014\_FOS | | Database | JASPAR\_JOLMA.meme | | *p*-value | 1.67974e-06 | | *E*-value | 0.00157896 | | *q*-value | 0.00261298 | | Overlap | 11 | | Offset | 0 | | Orientation | Normal | |  |
| Create custom LOGO ↧ | [Next Match] [Query Top] |
| Summary | Alignment |
| | Name | JASPAR2014\_JUND | | Database | JASPAR\_JOLMA.meme | | *p*-value | 2.81072e-06 | | *E*-value | 0.00264208 | | *q*-value | 0.00261298 | | Overlap | 11 | | Offset | 0 | | Orientation | Normal | |  |
| Create custom LOGO ↧ | [Previous Match] [Next Match] [Query Top] |
| Summary | Alignment |
| | Name | JOLMA2013\_JDP2\_full | | Database | JASPAR\_JOLMA.meme | | *p*-value | 7.58705e-06 | | *E*-value | 0.00713183 | | *q*-value | 0.00461125 | | Overlap | 9 | | Offset | -1 | | Orientation | Normal | |  |
| Create custom LOGO ↧ | [Previous Match] [Next Match] [Query Top] |
| Summary | Alignment |
| | Name | JOLMA2013\_Jdp2\_DBD | | Database | JASPAR\_JOLMA.meme | | *p*-value | 9.92043e-06 | | *E*-value | 0.0093252 | | *q*-value | 0.00461125 | | Overlap | 9 | | Offset | -1 | | Orientation | Normal | |  |
| Create custom LOGO ↧ | [Previous Match] [Next Match] [Query Top] |
| Summary | Alignment |
| | Name | JOLMA2013\_JDP2\_DBD | | Database | JASPAR\_JOLMA.meme | | *p*-value | 1.75681e-05 | | *E*-value | 0.016514 | | *q*-value | 0.00653287 | | Overlap | 9 | | Offset | -1 | | Orientation | Normal | |  |
| Create custom LOGO ↧ | [Previous Match] [Next Match] [Query Top] |
| Summary | Alignment |
| | Name | JASPAR2014\_JUNB | | Database | JASPAR\_JOLMA.meme | | *p*-value | 3.59243e-05 | | *E*-value | 0.0337689 | | *q*-value | 0.00954199 | | Overlap | 10 | | Offset | 1 | | Orientation | Normal | |  |
| Create custom LOGO ↧ | [Previous Match] [Next Match] [Query Top] |
| Summary | Alignment |
| | Name | JASPAR2014\_FOSL1 | | Database | JASPAR\_JOLMA.meme | | *p*-value | 6.17827e-05 | | *E*-value | 0.0580757 | | *q*-value | 0.0111527 | | Overlap | 11 | | Offset | 0 | | Orientation | Normal | |  |
| Create custom LOGO ↧ | [Previous Match] [Next Match] [Query Top] |
| Summary | Alignment |
| | Name | JOLMA2013\_NFE2\_DBD | | Database | JASPAR\_JOLMA.meme | | *p*-value | 6.17827e-05 | | *E*-value | 0.0580757 | | *q*-value | 0.0111527 | | Overlap | 11 | | Offset | 0 | | Orientation | Normal | |  |
| Create custom LOGO ↧ | [Previous Match] [Next Match] [Query Top] |
| Summary | Alignment |
| | Name | JASPAR2014\_JUN::FOS | | Database | JASPAR\_JOLMA.meme | | *p*-value | 6.20533e-05 | | *E*-value | 0.0583301 | | *q*-value | 0.0111527 | | Overlap | 7 | | Offset | -2 | | Orientation | Normal | |  |
| Create custom LOGO ↧ | [Previous Match] [Next Match] [Query Top] |
| Summary | Alignment |
| | Name | JASPAR2014\_FOSL2 | | Database | JASPAR\_JOLMA.meme | | *p*-value | 8.81349e-05 | | *E*-value | 0.0828468 | | *q*-value | 0.0126053 | | Overlap | 10 | | Offset | 1 | | Orientation | Normal | |  |
| Create custom LOGO ↧ | [Previous Match] [Query Top] |

|  |  |
| --- | --- |
| Matches to Query: M56 (O56) | Previous Next Top |

| Summary | Alignment |
| | Name | JOLMA2013\_FOXC1\_DBD | | Database | JASPAR\_JOLMA.meme | | *p*-value | 3.33549e-06 | | *E*-value | 0.00313536 | | *q*-value | 0.00613939 | | Overlap | 13 | | Offset | 0 | | Orientation | Normal | |  |
| Create custom LOGO ↧ | [Query Top] |

|  |  |
| --- | --- |
| Matches to Query: M57 (O57) | Previous Next Top |

|  |  |
| --- | --- |
| Matches to Query: M58 (O58) | Previous Next Top |

|  |  |
| --- | --- |
| Matches to Query: M59 (O59) | Previous Next Top |

|  |  |
| --- | --- |
| Matches to Query: M60 (O60) | Previous Next Top |

|  |  |
| --- | --- |
| Matches to Query: M61 (O61) | Previous Next Top |

|  |  |
| --- | --- |
| Matches to Query: M62 (O62) | Previous Next Top |

|  |  |
| --- | --- |
| Matches to Query: M63 (O63) | Previous Next Top |

|  |  |
| --- | --- |
| Matches to Query: M64 (O64) | Previous Next Top |

|  |  |
| --- | --- |
| Matches to Query: M65 (O65) | Previous Next Top |

|  |  |
| --- | --- |
| Matches to Query: M67 (O67) | Previous Next Top |

|  |  |
| --- | --- |
| Matches to Query: M68 (O68) | Previous Next Top |

| Summary | Alignment |
| | Name | JOLMA2013\_FOXC1\_DBD | | Database | JASPAR\_JOLMA.meme | | *p*-value | 2.80368e-06 | | *E*-value | 0.00263546 | | *q*-value | 0.00511528 | | Overlap | 13 | | Offset | -2 | | Orientation | Normal | |  |
| Create custom LOGO ↧ | [Next Match] [Query Top] |
| Summary | Alignment |
| | Name | JOLMA2013\_Foxc1\_DBD | | Database | JASPAR\_JOLMA.meme | | *p*-value | 4.97806e-05 | | *E*-value | 0.0467938 | | *q*-value | 0.0340729 | | Overlap | 11 | | Offset | -4 | | Orientation | Normal | |  |
| Create custom LOGO ↧ | [Previous Match] [Next Match] [Query Top] |
| Summary | Alignment |
| | Name | JOLMA2013\_FOXC2\_DBD\_2 | | Database | JASPAR\_JOLMA.meme | | *p*-value | 5.6026e-05 | | *E*-value | 0.0526644 | | *q*-value | 0.0340729 | | Overlap | 11 | | Offset | -4 | | Orientation | Normal | |  |
| Create custom LOGO ↧ | [Previous Match] [Next Match] [Query Top] |
| Summary | Alignment |
| | Name | JOLMA2013\_FOXC2\_DBD\_3 | | Database | JASPAR\_JOLMA.meme | | *p*-value | 9.01815e-05 | | *E*-value | 0.0847706 | | *q*-value | 0.0411338 | | Overlap | 12 | | Offset | -1 | | Orientation | Normal | |  |
| Create custom LOGO ↧ | [Previous Match] [Query Top] |

|  |  |
| --- | --- |
| Matches to Query: M71 (O71) | Previous Next Top |

|  |  |
| --- | --- |
| Matches to Query: M74 (O74) | Previous Next Top |

|  |  |
| --- | --- |
| Matches to Query: M75 (O75) | Previous Next Top |

| Summary | Alignment |
| | Name | JOLMA2013\_Mafb\_DBD\_3 | | Database | JASPAR\_JOLMA.meme | | *p*-value | 9.35831e-05 | | *E*-value | 0.0879681 | | *q*-value | 0.098018 | | Overlap | 11 | | Offset | 3 | | Orientation | Normal | |  |
| Create custom LOGO ↧ | [Query Top] |

|  |  |
| --- | --- |
| Matches to Query: M76 (O76) | Previous Next Top |

|  |  |
| --- | --- |
| Matches to Query: M77 (O77) | Previous Next Top |

| Summary | Alignment |
| | Name | JOLMA2013\_JDP2\_full | | Database | JASPAR\_JOLMA.meme | | *p*-value | 1.19939e-06 | | *E*-value | 0.00112742 | | *q*-value | 0.00111621 | | Overlap | 9 | | Offset | -1 | | Orientation | Normal | |  |
| Create custom LOGO ↧ | [Next Match] [Query Top] |
| Summary | Alignment |
| | Name | JOLMA2013\_Jdp2\_DBD | | Database | JASPAR\_JOLMA.meme | | *p*-value | 1.19939e-06 | | *E*-value | 0.00112742 | | *q*-value | 0.00111621 | | Overlap | 9 | | Offset | -1 | | Orientation | Normal | |  |
| Create custom LOGO ↧ | [Previous Match] [Next Match] [Query Top] |
| Summary | Alignment |
| | Name | JOLMA2013\_JDP2\_DBD | | Database | JASPAR\_JOLMA.meme | | *p*-value | 7.71716e-06 | | *E*-value | 0.00725413 | | *q*-value | 0.004788 | | Overlap | 9 | | Offset | -1 | | Orientation | Normal | |  |
| Create custom LOGO ↧ | [Previous Match] [Next Match] [Query Top] |
| Summary | Alignment |
| | Name | JOLMA2013\_NFE2\_DBD | | Database | JASPAR\_JOLMA.meme | | *p*-value | 4.79703e-05 | | *E*-value | 0.0450921 | | *q*-value | 0.0127553 | | Overlap | 11 | | Offset | 0 | | Orientation | Normal | |  |
| Create custom LOGO ↧ | [Previous Match] [Next Match] [Query Top] |
| Summary | Alignment |
| | Name | JASPAR2014\_JUN::FOS | | Database | JASPAR\_JOLMA.meme | | *p*-value | 0.000103903 | | *E*-value | 0.0976692 | | *q*-value | 0.0218967 | | Overlap | 7 | | Offset | -2 | | Orientation | Normal | |  |
| Create custom LOGO ↧ | [Previous Match] [Next Match] [Query Top] |
| Summary | Alignment |
| | Name | JASPAR2014\_FOS | | Database | JASPAR\_JOLMA.meme | | *p*-value | 0.000105877 | | *E*-value | 0.0995246 | | *q*-value | 0.0218967 | | Overlap | 11 | | Offset | 0 | | Orientation | Normal | |  |
| Create custom LOGO ↧ | [Previous Match] [Query Top] |

|  |  |
| --- | --- |
| Matches to Query: M78 (O78) | Previous Next Top |

|  |  |
| --- | --- |
| Matches to Query: M79 (O79) | Previous Next Top |

|  |  |
| --- | --- |
| Matches to Query: M80 (O80) | Previous Next Top |

|  |  |
| --- | --- |
| Matches to Query: M81 (O81) | Previous Next Top |

|  |  |
| --- | --- |
| Matches to Query: M82 (O82) | Previous Next Top |

|  |  |
| --- | --- |
| Matches to Query: M83 (O83) | Previous Next Top |

|  |  |
| --- | --- |
| Matches to Query: M84 (O84) | Previous Next Top |

|  |  |
| --- | --- |
| Matches to Query: M85 (O85) | Previous Next Top |

|  |  |
| --- | --- |
| Matches to Query: M86 (O86) | Previous Next Top |

|  |  |
| --- | --- |
| Matches to Query: M87 (O87) | Previous Next Top |

| Summary | Alignment |
| | Name | JOLMA2013\_NFE2\_DBD | | Database | JASPAR\_JOLMA.meme | | *p*-value | 8.4256e-07 | | *E*-value | 0.000792006 | | *q*-value | 0.00156657 | | Overlap | 11 | | Offset | 0 | | Orientation | Normal | |  |
| Create custom LOGO ↧ | [Next Match] [Query Top] |
| Summary | Alignment |
| | Name | JOLMA2013\_JDP2\_full | | Database | JASPAR\_JOLMA.meme | | *p*-value | 3.20281e-06 | | *E*-value | 0.00301064 | | *q*-value | 0.00198499 | | Overlap | 9 | | Offset | -1 | | Orientation | Normal | |  |
| Create custom LOGO ↧ | [Previous Match] [Next Match] [Query Top] |
| Summary | Alignment |
| | Name | JOLMA2013\_Jdp2\_DBD | | Database | JASPAR\_JOLMA.meme | | *p*-value | 3.20281e-06 | | *E*-value | 0.00301064 | | *q*-value | 0.00198499 | | Overlap | 9 | | Offset | -1 | | Orientation | Normal | |  |
| Create custom LOGO ↧ | [Previous Match] [Next Match] [Query Top] |
| Summary | Alignment |
| | Name | JOLMA2013\_JDP2\_DBD | | Database | JASPAR\_JOLMA.meme | | *p*-value | 1.11613e-05 | | *E*-value | 0.0104916 | | *q*-value | 0.00478501 | | Overlap | 9 | | Offset | -1 | | Orientation | Normal | |  |
| Create custom LOGO ↧ | [Previous Match] [Next Match] [Query Top] |
| Summary | Alignment |
| | Name | JASPAR2014\_JUND | | Database | JASPAR\_JOLMA.meme | | *p*-value | 1.28678e-05 | | *E*-value | 0.0120957 | | *q*-value | 0.00478501 | | Overlap | 11 | | Offset | 0 | | Orientation | Normal | |  |
| Create custom LOGO ↧ | [Previous Match] [Next Match] [Query Top] |
| Summary | Alignment |
| | Name | JASPAR2014\_Nfe2l2 | | Database | JASPAR\_JOLMA.meme | | *p*-value | 7.94054e-05 | | *E*-value | 0.074641 | | *q*-value | 0.0135248 | | Overlap | 12 | | Offset | 3 | | Orientation | Normal | |  |
| Create custom LOGO ↧ | [Previous Match] [Next Match] [Query Top] |
| Summary | Alignment |
| | Name | JASPAR2014\_FOS | | Database | JASPAR\_JOLMA.meme | | *p*-value | 8.00154e-05 | | *E*-value | 0.0752145 | | *q*-value | 0.0135248 | | Overlap | 11 | | Offset | 0 | | Orientation | Reverse Complement | |  |
| Create custom LOGO ↧ | [Previous Match] [Next Match] [Query Top] |
| Summary | Alignment |
| | Name | JASPAR2014\_FOSL1 | | Database | JASPAR\_JOLMA.meme | | *p*-value | 8.00154e-05 | | *E*-value | 0.0752145 | | *q*-value | 0.0135248 | | Overlap | 11 | | Offset | 0 | | Orientation | Normal | |  |
| Create custom LOGO ↧ | [Previous Match] [Query Top] |

|  |  |
| --- | --- |
| Matches to Query: M88 (O88) | Previous Next Top |

|  |  |
| --- | --- |
| Matches to Query: M89 (O89) | Previous Next Top |

| Summary | Alignment |
| | Name | JASPAR2014\_Rfx1 | | Database | JASPAR\_JOLMA.meme | | *p*-value | 4.00998e-09 | | *E*-value | 3.76938e-06 | | *q*-value | 6.15254e-06 | | Overlap | 14 | | Offset | 0 | | Orientation | Normal | |  |
| Create custom LOGO ↧ | [Next Match] [Query Top] |
| Summary | Alignment |
| | Name | JASPAR2014\_RFX2 | | Database | JASPAR\_JOLMA.meme | | *p*-value | 6.61456e-09 | | *E*-value | 6.21769e-06 | | *q*-value | 6.15254e-06 | | Overlap | 14 | | Offset | 0 | | Orientation | Normal | |  |
| Create custom LOGO ↧ | [Previous Match] [Next Match] [Query Top] |
| Summary | Alignment |
| | Name | JOLMA2013\_RFX3\_DBD | | Database | JASPAR\_JOLMA.meme | | *p*-value | 7.97211e-08 | | *E*-value | 7.49379e-05 | | *q*-value | 2.19906e-05 | | Overlap | 14 | | Offset | 1 | | Orientation | Reverse Complement | |  |
| Create custom LOGO ↧ | [Previous Match] [Next Match] [Query Top] |
| Summary | Alignment |
| | Name | JOLMA2013\_RFX2\_DBD | | Database | JASPAR\_JOLMA.meme | | *p*-value | 1.10444e-07 | | *E*-value | 0.000103817 | | *q*-value | 2.19906e-05 | | Overlap | 14 | | Offset | 1 | | Orientation | Reverse Complement | |  |
| Create custom LOGO ↧ | [Previous Match] [Next Match] [Query Top] |
| Summary | Alignment |
| | Name | JOLMA2013\_RFX5\_DBD\_2 | | Database | JASPAR\_JOLMA.meme | | *p*-value | 1.10444e-07 | | *E*-value | 0.000103817 | | *q*-value | 2.19906e-05 | | Overlap | 14 | | Offset | 1 | | Orientation | Normal | |  |
| Create custom LOGO ↧ | [Previous Match] [Next Match] [Query Top] |
| Summary | Alignment |
| | Name | JOLMA2013\_Rfx3\_DBD | | Database | JASPAR\_JOLMA.meme | | *p*-value | 1.10444e-07 | | *E*-value | 0.000103817 | | *q*-value | 2.19906e-05 | | Overlap | 14 | | Offset | 1 | | Orientation | Reverse Complement | |  |
| Create custom LOGO ↧ | [Previous Match] [Next Match] [Query Top] |
| Summary | Alignment |
| | Name | JOLMA2013\_Rfx2\_DBD | | Database | JASPAR\_JOLMA.meme | | *p*-value | 1.30451e-07 | | *E*-value | 0.000122624 | | *q*-value | 2.19906e-05 | | Overlap | 14 | | Offset | 1 | | Orientation | Normal | |  |
| Create custom LOGO ↧ | [Previous Match] [Next Match] [Query Top] |
| Summary | Alignment |
| | Name | JOLMA2013\_RFX5\_DBD | | Database | JASPAR\_JOLMA.meme | | *p*-value | 1.41852e-07 | | *E*-value | 0.000133341 | | *q*-value | 2.19906e-05 | | Overlap | 14 | | Offset | 1 | | Orientation | Reverse Complement | |  |
| Create custom LOGO ↧ | [Previous Match] [Next Match] [Query Top] |
| Summary | Alignment |
| | Name | JOLMA2013\_RFX4\_DBD | | Database | JASPAR\_JOLMA.meme | | *p*-value | 3.20942e-07 | | *E*-value | 0.000301686 | | *q*-value | 3.73156e-05 | | Overlap | 14 | | Offset | 1 | | Orientation | Normal | |  |
| Create custom LOGO ↧ | [Previous Match] [Next Match] [Query Top] |
| Summary | Alignment |
| | Name | JOLMA2013\_NFATC1\_full\_3 | | Database | JASPAR\_JOLMA.meme | | *p*-value | 2.91197e-05 | | *E*-value | 0.0273725 | | *q*-value | 0.00285113 | | Overlap | 14 | | Offset | 0 | | Orientation | Reverse Complement | |  |
| Create custom LOGO ↧ | [Previous Match] [Next Match] [Query Top] |
| Summary | Alignment |
| | Name | JASPAR2014\_RFX5 | | Database | JASPAR\_JOLMA.meme | | *p*-value | 3.49051e-05 | | *E*-value | 0.0328108 | | *q*-value | 0.0030921 | | Overlap | 12 | | Offset | -2 | | Orientation | Normal | |  |
| Create custom LOGO ↧ | [Previous Match] [Query Top] |

|  |  |
| --- | --- |
| Matches to Query: M90 (O90) | Previous Next Top |

|  |  |
| --- | --- |
| Matches to Query: M91 (O91) | Previous Next Top |

| Summary | Alignment |
| | Name | JOLMA2013\_JDP2\_DBD | | Database | JASPAR\_JOLMA.meme | | *p*-value | 2.37577e-08 | | *E*-value | 2.23322e-05 | | *q*-value | 2.21579e-05 | | Overlap | 9 | | Offset | -3 | | Orientation | Normal | |  |
| Create custom LOGO ↧ | [Next Match] [Query Top] |
| Summary | Alignment |
| | Name | JOLMA2013\_JDP2\_full | | Database | JASPAR\_JOLMA.meme | | *p*-value | 1.31843e-07 | | *E*-value | 0.000123932 | | *q*-value | 7.82344e-05 | | Overlap | 9 | | Offset | -3 | | Orientation | Normal | |  |
| Create custom LOGO ↧ | [Previous Match] [Next Match] [Query Top] |
| Summary | Alignment |
| | Name | JOLMA2013\_Jdp2\_DBD | | Database | JASPAR\_JOLMA.meme | | *p*-value | 2.09707e-07 | | *E*-value | 0.000197125 | | *q*-value | 7.82344e-05 | | Overlap | 9 | | Offset | -3 | | Orientation | Normal | |  |
| Create custom LOGO ↧ | [Previous Match] [Next Match] [Query Top] |
| Summary | Alignment |
| | Name | JASPAR2014\_FOS | | Database | JASPAR\_JOLMA.meme | | *p*-value | 3.71283e-06 | | *E*-value | 0.00349006 | | *q*-value | 0.000981064 | | Overlap | 11 | | Offset | -2 | | Orientation | Reverse Complement | |  |
| Create custom LOGO ↧ | [Previous Match] [Next Match] [Query Top] |
| Summary | Alignment |
| | Name | JASPAR2014\_BATF::JUN | | Database | JASPAR\_JOLMA.meme | | *p*-value | 4.20758e-06 | | *E*-value | 0.00395512 | | *q*-value | 0.000981064 | | Overlap | 10 | | Offset | -4 | | Orientation | Reverse Complement | |  |
| Create custom LOGO ↧ | [Previous Match] [Next Match] [Query Top] |
| Summary | Alignment |
| | Name | JASPAR2014\_JUNB | | Database | JASPAR\_JOLMA.meme | | *p*-value | 5.34848e-06 | | *E*-value | 0.00502758 | | *q*-value | 0.000997667 | | Overlap | 11 | | Offset | -1 | | Orientation | Normal | |  |
| Create custom LOGO ↧ | [Previous Match] [Next Match] [Query Top] |
| Summary | Alignment |
| | Name | JASPAR2014\_JUND | | Database | JASPAR\_JOLMA.meme | | *p*-value | 8.37949e-06 | | *E*-value | 0.00787672 | | *q*-value | 0.00142095 | | Overlap | 11 | | Offset | -2 | | Orientation | Reverse Complement | |  |
| Create custom LOGO ↧ | [Previous Match] [Next Match] [Query Top] |
| Summary | Alignment |
| | Name | JASPAR2014\_FOSL2 | | Database | JASPAR\_JOLMA.meme | | *p*-value | 9.32891e-06 | | *E*-value | 0.00876917 | | *q*-value | 0.00145012 | | Overlap | 11 | | Offset | -1 | | Orientation | Normal | |  |
| Create custom LOGO ↧ | [Previous Match] [Next Match] [Query Top] |
| Summary | Alignment |
| | Name | JOLMA2013\_NFE2\_DBD | | Database | JASPAR\_JOLMA.meme | | *p*-value | 2.88228e-05 | | *E*-value | 0.0270934 | | *q*-value | 0.00384028 | | Overlap | 11 | | Offset | -2 | | Orientation | Normal | |  |
| Create custom LOGO ↧ | [Previous Match] [Next Match] [Query Top] |
| Summary | Alignment |
| | Name | JASPAR2014\_JUN::FOS | | Database | JASPAR\_JOLMA.meme | | *p*-value | 6.06657e-05 | | *E*-value | 0.0570257 | | *q*-value | 0.00628674 | | Overlap | 7 | | Offset | -4 | | Orientation | Normal | |  |
| Create custom LOGO ↧ | [Previous Match] [Query Top] |

|  |  |
| --- | --- |
| Matches to Query: M92 (O92) | Previous Next Top |

|  |  |
| --- | --- |
| Matches to Query: M93 (O93) | Previous Next Top |

|  |  |
| --- | --- |
| Matches to Query: M94 (O94) | Previous Next Top |

|  |  |
| --- | --- |
| Matches to Query: M95 (O95) | Previous Next Top |

|  |  |
| --- | --- |
| Matches to Query: M96 (O96) | Previous Next Top |

| Summary | Alignment |
| | Name | JASPAR2014\_SP1 | | Database | JASPAR\_JOLMA.meme | | *p*-value | 8.57357e-05 | | *E*-value | 0.0805916 | | *q*-value | 0.147615 | | Overlap | 11 | | Offset | -2 | | Orientation | Reverse Complement | |  |
| Create custom LOGO ↧ | [Query Top] |

|  |  |
| --- | --- |
| Matches to Query: M97 (O97) | Previous Next Top |

|  |  |
| --- | --- |
| Matches to Query: M98 (O98) | Previous Next Top |

|  |  |
| --- | --- |
| Matches to Query: M99 (O99) | Previous Next Top |

|  |  |
| --- | --- |
| Matches to Query: M100 (O100) | Previous Next Top |

|  |  |
| --- | --- |
| Matches to Query: M101 (O101) | Previous Next Top |

|  |  |
| --- | --- |
| Matches to Query: M102 (O102) | Previous Next Top |

|  |  |
| --- | --- |
| Matches to Query: M103 (O103) | Previous Next Top |

|  |  |
| --- | --- |
| Matches to Query: M104 (O104) | Previous Next Top |

|  |  |
| --- | --- |
| Matches to Query: M106 (O106) | Previous Next Top |

|  |  |
| --- | --- |
| Matches to Query: M108 (O108) | Previous Next Top |

|  |  |
| --- | --- |
| Matches to Query: M110 (O110) | Previous Next Top |

|  |  |
| --- | --- |
| Matches to Query: M112 (O112) | Previous Next Top |

|  |  |
| --- | --- |
| Matches to Query: M113 (O113) | Previous Next Top |

| Summary | Alignment |
| | Name | JOLMA2013\_FOXC1\_DBD | | Database | JASPAR\_JOLMA.meme | | *p*-value | 4.59293e-05 | | *E*-value | 0.0431736 | | *q*-value | 0.0841802 | | Overlap | 10 | | Offset | 0 | | Orientation | Normal | |  |
| Create custom LOGO ↧ | [Query Top] |

|  |  |
| --- | --- |
| Matches to Query: M114 (O114) | Previous Next Top |

| Summary | Alignment |
| | Name | JASPAR2014\_TFAP2C | | Database | JASPAR\_JOLMA.meme | | *p*-value | 1.28902e-05 | | *E*-value | 0.0121168 | | *q*-value | 0.0139841 | | Overlap | 10 | | Offset | 2 | | Orientation | Reverse Complement | |  |
| Create custom LOGO ↧ | [Next Match] [Query Top] |
| Summary | Alignment |
| | Name | JOLMA2013\_TFAP2C\_full\_3 | | Database | JASPAR\_JOLMA.meme | | *p*-value | 1.65037e-05 | | *E*-value | 0.0155135 | | *q*-value | 0.0139841 | | Overlap | 10 | | Offset | 1 | | Orientation | Reverse Complement | |  |
| Create custom LOGO ↧ | [Previous Match] [Next Match] [Query Top] |
| Summary | Alignment |
| | Name | JOLMA2013\_TFAP2B\_DBD\_2 | | Database | JASPAR\_JOLMA.meme | | *p*-value | 2.39022e-05 | | *E*-value | 0.022468 | | *q*-value | 0.0139841 | | Overlap | 10 | | Offset | 1 | | Orientation | Reverse Complement | |  |
| Create custom LOGO ↧ | [Previous Match] [Next Match] [Query Top] |
| Summary | Alignment |
| | Name | JOLMA2013\_Tcfap2a\_DBD\_2 | | Database | JASPAR\_JOLMA.meme | | *p*-value | 3.12101e-05 | | *E*-value | 0.0293375 | | *q*-value | 0.0139841 | | Overlap | 10 | | Offset | 1 | | Orientation | Reverse Complement | |  |
| Create custom LOGO ↧ | [Previous Match] [Next Match] [Query Top] |
| Summary | Alignment |
| | Name | JOLMA2013\_TFAP2A\_DBD\_2 | | Database | JASPAR\_JOLMA.meme | | *p*-value | 3.76816e-05 | | *E*-value | 0.0354207 | | *q*-value | 0.0139841 | | Overlap | 10 | | Offset | 1 | | Orientation | Reverse Complement | |  |
| Create custom LOGO ↧ | [Previous Match] [Next Match] [Query Top] |
| Summary | Alignment |
| | Name | JOLMA2013\_TFAP2C\_DBD\_2 | | Database | JASPAR\_JOLMA.meme | | *p*-value | 7.17287e-05 | | *E*-value | 0.067425 | | *q*-value | 0.0221828 | | Overlap | 10 | | Offset | 1 | | Orientation | Reverse Complement | |  |
| Create custom LOGO ↧ | [Previous Match] [Query Top] |

|  |  |
| --- | --- |
| Matches to Query: M115 (O115) | Previous Next Top |

|  |  |
| --- | --- |
| Matches to Query: M116 (O116) | Previous Next Top |

|  |  |
| --- | --- |
| Matches to Query: M117 (O117) | Previous Next Top |

|  |  |
| --- | --- |
| Matches to Query: M118 (O118) | Previous Next Top |

| Summary | Alignment |
| | Name | JASPAR2014\_NHLH1 | | Database | JASPAR\_JOLMA.meme | | *p*-value | 9.99684e-05 | | *E*-value | 0.0939703 | | *q*-value | 0.100528 | | Overlap | 10 | | Offset | 0 | | Orientation | Reverse Complement | |  |
| Create custom LOGO ↧ | [Query Top] |

|  |  |
| --- | --- |
| Matches to Query: M119 (O119) | Previous Next Top |

|  |  |
| --- | --- |
| Matches to Query: M120 (O120) | Previous Next Top |

|  |  |
| --- | --- |
| Matches to Query: M121 (O121) | Previous Next Top |

|  |  |
| --- | --- |
| Matches to Query: M122 (O122) | Previous Next Top |

| Summary | Alignment |
| | Name | JASPAR2014\_Meis1 | | Database | JASPAR\_JOLMA.meme | | *p*-value | 9.30961e-05 | | *E*-value | 0.0875104 | | *q*-value | 0.175021 | | Overlap | 10 | | Offset | 4 | | Orientation | Reverse Complement | |  |
| Create custom LOGO ↧ | [Query Top] |

|  |  |
| --- | --- |
| Matches to Query: M123 (O123) | Previous Next Top |

|  |  |
| --- | --- |
| Matches to Query: M124 (O124) | Previous Next Top |

|  |  |
| --- | --- |
| Matches to Query: M125 (O125) | Previous Next Top |

|  |  |
| --- | --- |
| Matches to Query: M126 (O126) | Previous Next Top |

|  |  |
| --- | --- |
| Matches to Query: M127 (O127) | Previous Next Top |

| Summary | Alignment |
| | Name | JASPAR2014\_HNF1B | | Database | JASPAR\_JOLMA.meme | | *p*-value | 4.07577e-06 | | *E*-value | 0.00383122 | | *q*-value | 0.00761083 | | Overlap | 11 | | Offset | 0 | | Orientation | Normal | |  |
| Create custom LOGO ↧ | [Next Match] [Query Top] |
| Summary | Alignment |
| | Name | JOLMA2013\_HNF1A\_full | | Database | JASPAR\_JOLMA.meme | | *p*-value | 4.1215e-05 | | *E*-value | 0.0387421 | | *q*-value | 0.0276333 | | Overlap | 11 | | Offset | 2 | | Orientation | Normal | |  |
| Create custom LOGO ↧ | [Previous Match] [Next Match] [Query Top] |
| Summary | Alignment |
| | Name | JOLMA2013\_HNF1B\_full | | Database | JASPAR\_JOLMA.meme | | *p*-value | 4.5006e-05 | | *E*-value | 0.0423056 | | *q*-value | 0.0276333 | | Overlap | 11 | | Offset | 1 | | Orientation | Normal | |  |
| Create custom LOGO ↧ | [Previous Match] [Next Match] [Query Top] |
| Summary | Alignment |
| | Name | JOLMA2013\_HNF1B\_full\_2 | | Database | JASPAR\_JOLMA.meme | | *p*-value | 9.61909e-05 | | *E*-value | 0.0904194 | | *q*-value | 0.0299368 | | Overlap | 11 | | Offset | 2 | | Orientation | Normal | |  |
| Create custom LOGO ↧ | [Previous Match] [Query Top] |

|  |  |
| --- | --- |
| Matches to Query: M128 (O128) | Previous Next Top |

|  |  |
| --- | --- |
| Matches to Query: M129 (O129) | Previous Next Top |

|  |  |
| --- | --- |
| Matches to Query: M130 (O130) | Previous Next Top |

|  |  |
| --- | --- |
| Matches to Query: M131 (O131) | Previous Next Top |

| Summary | Alignment |
| | Name | JASPAR2014\_Tcf3 | | Database | JASPAR\_JOLMA.meme | | *p*-value | 1.43366e-05 | | *E*-value | 0.0134764 | | *q*-value | 0.0268577 | | Overlap | 11 | | Offset | 0 | | Orientation | Reverse Complement | |  |
| Create custom LOGO ↧ | [Next Match] [Query Top] |
| Summary | Alignment |
| | Name | JASPAR2014\_Tcf12 | | Database | JASPAR\_JOLMA.meme | | *p*-value | 9.34492e-05 | | *E*-value | 0.0878423 | | *q*-value | 0.0688504 | | Overlap | 9 | | Offset | 2 | | Orientation | Normal | |  |
| Create custom LOGO ↧ | [Previous Match] [Query Top] |

|  |  |
| --- | --- |
| Matches to Query: M132 (O132) | Previous Next Top |

|  |  |
| --- | --- |
| Matches to Query: M133 (O133) | Previous Next Top |

| Summary | Alignment |
| | Name | JASPAR2014\_SMAD2::SMAD3::SMAD4 | | Database | JASPAR\_JOLMA.meme | | *p*-value | 5.69768e-05 | | *E*-value | 0.0535582 | | *q*-value | 0.107116 | | Overlap | 12 | | Offset | 1 | | Orientation | Reverse Complement | |  |
| Create custom LOGO ↧ | [Query Top] |

|  |  |
| --- | --- |
| Matches to Query: M134 (O134) | Previous Next Top |

|  |  |
| --- | --- |
| Matches to Query: M135 (O135) | Previous Next Top |

| Summary | Alignment |
| | Name | JASPAR2014\_Tcf12 | | Database | JASPAR\_JOLMA.meme | | *p*-value | 6.80893e-05 | | *E*-value | 0.0640039 | | *q*-value | 0.0533263 | | Overlap | 9 | | Offset | 2 | | Orientation | Normal | |  |
| Create custom LOGO ↧ | [Next Match] [Query Top] |
| Summary | Alignment |
| | Name | JASPAR2014\_Tcf3 | | Database | JASPAR\_JOLMA.meme | | *p*-value | 8.07735e-05 | | *E*-value | 0.075927 | | *q*-value | 0.0533263 | | Overlap | 9 | | Offset | 2 | | Orientation | Normal | |  |
| Create custom LOGO ↧ | [Previous Match] [Next Match] [Query Top] |
| Summary | Alignment |
| | Name | JASPAR2014\_Myog | | Database | JASPAR\_JOLMA.meme | | *p*-value | 9.59176e-05 | | *E*-value | 0.0901626 | | *q*-value | 0.0533263 | | Overlap | 11 | | Offset | 0 | | Orientation | Reverse Complement | |  |
| Create custom LOGO ↧ | [Previous Match] [Query Top] |

|  |  |
| --- | --- |
| Matches to Query: M136 (O136) | Previous Next Top |

|  |  |
| --- | --- |
| Matches to Query: M137 (O137) | Previous Next Top |

|  |  |
| --- | --- |
| Matches to Query: M138 (O138) | Previous Next Top |

|  |  |
| --- | --- |
| Matches to Query: M139 (O139) | Previous Next Top |

| Summary | Alignment |
| | Name | JASPAR2014\_Bach1::Mafk | | Database | JASPAR\_JOLMA.meme | | *p*-value | 6.17352e-06 | | *E*-value | 0.00580311 | | *q*-value | 0.0115218 | | Overlap | 11 | | Offset | -1 | | Orientation | Reverse Complement | |  |
| Create custom LOGO ↧ | [Next Match] [Query Top] |
| Summary | Alignment |
| | Name | JASPAR2014\_NFE2::MAF | | Database | JASPAR\_JOLMA.meme | | *p*-value | 2.62513e-05 | | *E*-value | 0.0246762 | | *q*-value | 0.0226174 | | Overlap | 12 | | Offset | 2 | | Orientation | Reverse Complement | |  |
| Create custom LOGO ↧ | [Previous Match] [Next Match] [Query Top] |
| Summary | Alignment |
| | Name | JASPAR2014\_Nfe2l2 | | Database | JASPAR\_JOLMA.meme | | *p*-value | 3.63558e-05 | | *E*-value | 0.0341745 | | *q*-value | 0.0226174 | | Overlap | 10 | | Offset | -2 | | Orientation | Reverse Complement | |  |
| Create custom LOGO ↧ | [Previous Match] [Query Top] |

|  |  |
| --- | --- |
| Matches to Query: M140 (O140) | Previous Next Top |

|  |  |
| --- | --- |
| Matches to Query: M141 (O141) | Previous Next Top |

|  |  |
| --- | --- |
| Matches to Query: M142 (O142) | Previous Next Top |

|  |  |
| --- | --- |
| Matches to Query: M143 (O143) | Previous Next Top |

| Summary | Alignment |
| | Name | JOLMA2013\_ONECUT3\_DBD | | Database | JASPAR\_JOLMA.meme | | *p*-value | 3.90215e-05 | | *E*-value | 0.0366802 | | *q*-value | 0.0728663 | | Overlap | 12 | | Offset | 1 | | Orientation | Normal | |  |
| Create custom LOGO ↧ | [Next Match] [Query Top] |
| Summary | Alignment |
| | Name | JOLMA2013\_ONECUT1\_DBD | | Database | JASPAR\_JOLMA.meme | | *p*-value | 9.77177e-05 | | *E*-value | 0.0918547 | | *q*-value | 0.0825512 | | Overlap | 12 | | Offset | 1 | | Orientation | Normal | |  |
| Create custom LOGO ↧ | [Previous Match] [Query Top] |

|  |  |
| --- | --- |
| Matches to Query: M144 (O144) | Previous Next Top |

|  |  |
| --- | --- |
| Matches to Query: M145 (O145) | Previous Next Top |

|  |  |
| --- | --- |
| Matches to Query: M146 (O146) | Previous Next Top |

| Summary | Alignment |
| | Name | JOLMA2013\_RFX4\_DBD\_2 | | Database | JASPAR\_JOLMA.meme | | *p*-value | 1.52892e-08 | | *E*-value | 1.43719e-05 | | *q*-value | 2.42049e-05 | | Overlap | 13 | | Offset | 1 | | Orientation | Reverse Complement | |  |
| Create custom LOGO ↧ | [Next Match] [Query Top] |
| Summary | Alignment |
| | Name | JOLMA2013\_Rfx2\_DBD\_2 | | Database | JASPAR\_JOLMA.meme | | *p*-value | 3.38342e-08 | | *E*-value | 3.18041e-05 | | *q*-value | 2.42049e-05 | | Overlap | 13 | | Offset | 1 | | Orientation | Reverse Complement | |  |
| Create custom LOGO ↧ | [Previous Match] [Next Match] [Query Top] |
| Summary | Alignment |
| | Name | JOLMA2013\_RFX2\_DBD\_2 | | Database | JASPAR\_JOLMA.meme | | *p*-value | 5.21297e-08 | | *E*-value | 4.90019e-05 | | *q*-value | 2.42049e-05 | | Overlap | 13 | | Offset | 1 | | Orientation | Reverse Complement | |  |
| Create custom LOGO ↧ | [Previous Match] [Next Match] [Query Top] |
| Summary | Alignment |
| | Name | JOLMA2013\_RFX3\_DBD\_2 | | Database | JASPAR\_JOLMA.meme | | *p*-value | 5.21297e-08 | | *E*-value | 4.90019e-05 | | *q*-value | 2.42049e-05 | | Overlap | 13 | | Offset | 1 | | Orientation | Reverse Complement | |  |
| Create custom LOGO ↧ | [Previous Match] [Next Match] [Query Top] |
| Summary | Alignment |
| | Name | JASPAR2014\_RFX5 | | Database | JASPAR\_JOLMA.meme | | *p*-value | 1.17693e-05 | | *E*-value | 0.0110632 | | *q*-value | 0.00242878 | | Overlap | 12 | | Offset | -1 | | Orientation | Normal | |  |
| Create custom LOGO ↧ | [Previous Match] [Query Top] |

|  |  |
| --- | --- |
| Matches to Query: M147 (O147) | Previous Next Top |

|  |  |
| --- | --- |
| Matches to Query: M148 (O148) | Previous Next Top |

|  |  |
| --- | --- |
| Matches to Query: M149 (O149) | Previous Next Top |

|  |  |
| --- | --- |
| Matches to Query: M150 (O150) | Previous Next Top |

| Summary | Alignment |
| | Name | JOLMA2013\_ONECUT3\_DBD | | Database | JASPAR\_JOLMA.meme | | *p*-value | 4.71635e-06 | | *E*-value | 0.00443337 | | *q*-value | 0.00869532 | | Overlap | 13 | | Offset | 0 | | Orientation | Normal | |  |
| Create custom LOGO ↧ | [Next Match] [Query Top] |
| Summary | Alignment |
| | Name | JOLMA2013\_ONECUT1\_DBD | | Database | JASPAR\_JOLMA.meme | | *p*-value | 1.66555e-05 | | *E*-value | 0.0156562 | | *q*-value | 0.0153535 | | Overlap | 13 | | Offset | 0 | | Orientation | Normal | |  |
| Create custom LOGO ↧ | [Previous Match] [Next Match] [Query Top] |
| Summary | Alignment |
| | Name | JOLMA2013\_ONECUT2\_DBD | | Database | JASPAR\_JOLMA.meme | | *p*-value | 3.2252e-05 | | *E*-value | 0.0303169 | | *q*-value | 0.0198205 | | Overlap | 13 | | Offset | 0 | | Orientation | Normal | |  |
| Create custom LOGO ↧ | [Previous Match] [Next Match] [Query Top] |
| Summary | Alignment |
| | Name | JOLMA2013\_ONECUT1\_full | | Database | JASPAR\_JOLMA.meme | | *p*-value | 6.00755e-05 | | *E*-value | 0.056471 | | *q*-value | 0.0232492 | | Overlap | 13 | | Offset | 0 | | Orientation | Normal | |  |
| Create custom LOGO ↧ | [Previous Match] [Next Match] [Query Top] |
| Summary | Alignment |
| | Name | JOLMA2013\_FOXL1\_full\_2 | | Database | JASPAR\_JOLMA.meme | | *p*-value | 6.30518e-05 | | *E*-value | 0.0592687 | | *q*-value | 0.0232492 | | Overlap | 13 | | Offset | 0 | | Orientation | Normal | |  |
| Create custom LOGO ↧ | [Previous Match] [Query Top] |

|  |  |
| --- | --- |
| Matches to Query: M151 (O151) | Previous Next Top |

|  |  |
| --- | --- |
| Matches to Query: M152 (O152) | Previous Next Top |

| Summary | Alignment |
| | Name | JASPAR2014\_FOXP1 | | Database | JASPAR\_JOLMA.meme | | *p*-value | 9.25379e-07 | | *E*-value | 0.000869856 | | *q*-value | 0.00171709 | | Overlap | 14 | | Offset | 0 | | Orientation | Reverse Complement | |  |
| Create custom LOGO ↧ | [Next Match] [Query Top] |
| Summary | Alignment |
| | Name | JASPAR2014\_FOXP2 | | Database | JASPAR\_JOLMA.meme | | *p*-value | 9.74467e-06 | | *E*-value | 0.00915999 | | *q*-value | 0.00904089 | | Overlap | 11 | | Offset | -1 | | Orientation | Reverse Complement | |  |
| Create custom LOGO ↧ | [Previous Match] [Next Match] [Query Top] |
| Summary | Alignment |
| | Name | JOLMA2013\_FOXC2\_DBD\_3 | | Database | JASPAR\_JOLMA.meme | | *p*-value | 5.13469e-05 | | *E*-value | 0.0482661 | | *q*-value | 0.031759 | | Overlap | 12 | | Offset | -1 | | Orientation | Reverse Complement | |  |
| Create custom LOGO ↧ | [Previous Match] [Query Top] |

|  |  |
| --- | --- |
| Matches to Query: M153 (O153) | Previous Next Top |

|  |  |
| --- | --- |
| Matches to Query: M154 (O154) | Previous Next Top |

| Summary | Alignment |
| | Name | JOLMA2013\_ONECUT3\_DBD | | Database | JASPAR\_JOLMA.meme | | *p*-value | 4.54292e-05 | | *E*-value | 0.0427035 | | *q*-value | 0.0531136 | | Overlap | 14 | | Offset | 0 | | Orientation | Normal | |  |
| Create custom LOGO ↧ | [Next Match] [Query Top] |
| Summary | Alignment |
| | Name | JOLMA2013\_ONECUT1\_DBD | | Database | JASPAR\_JOLMA.meme | | *p*-value | 7.06377e-05 | | *E*-value | 0.0663994 | | *q*-value | 0.0531136 | | Overlap | 14 | | Offset | 0 | | Orientation | Normal | |  |
| Create custom LOGO ↧ | [Previous Match] [Next Match] [Query Top] |
| Summary | Alignment |
| | Name | JOLMA2013\_FOXL1\_full\_2 | | Database | JASPAR\_JOLMA.meme | | *p*-value | 9.16132e-05 | | *E*-value | 0.0861164 | | *q*-value | 0.0531136 | | Overlap | 13 | | Offset | 0 | | Orientation | Normal | |  |
| Create custom LOGO ↧ | [Previous Match] [Query Top] |

|  |  |
| --- | --- |
| Matches to Query: M155 (O155) | Previous Next Top |

| Summary | Alignment |
| | Name | JASPAR2014\_Nfe2l2 | | Database | JASPAR\_JOLMA.meme | | *p*-value | 6.0193e-05 | | *E*-value | 0.0565815 | | *q*-value | 0.113163 | | Overlap | 13 | | Offset | 2 | | Orientation | Reverse Complement | |  |
| Create custom LOGO ↧ | [Query Top] |

|  |  |
| --- | --- |
| Matches to Query: M156 (O156) | Previous Next Top |

|  |  |
| --- | --- |
| Matches to Query: M157 (O157) | Previous Next Top |

|  |  |
| --- | --- |
| Matches to Query: M158 (O158) | Previous Next Top |

|  |  |
| --- | --- |
| Matches to Query: M159 (O159) | Previous Next Top |

|  |  |
| --- | --- |
| Matches to Query: M160 (O160) | Previous Next Top |

| Summary | Alignment |
| | Name | JOLMA2013\_HNF1B\_full | | Database | JASPAR\_JOLMA.meme | | *p*-value | 1.14283e-07 | | *E*-value | 0.000107426 | | *q*-value | 7.16364e-05 | | Overlap | 13 | | Offset | -1 | | Orientation | Normal | |  |
| Create custom LOGO ↧ | [Next Match] [Query Top] |
| Summary | Alignment |
| | Name | JOLMA2013\_HNF1B\_full\_2 | | Database | JASPAR\_JOLMA.meme | | *p*-value | 1.28158e-07 | | *E*-value | 0.000120469 | | *q*-value | 7.16364e-05 | | Overlap | 14 | | Offset | 0 | | Orientation | Reverse Complement | |  |
| Create custom LOGO ↧ | [Previous Match] [Next Match] [Query Top] |
| Summary | Alignment |
| | Name | JASPAR2014\_HNF1A | | Database | JASPAR\_JOLMA.meme | | *p*-value | 1.57094e-07 | | *E*-value | 0.000147668 | | *q*-value | 7.16364e-05 | | Overlap | 14 | | Offset | 0 | | Orientation | Normal | |  |
| Create custom LOGO ↧ | [Previous Match] [Next Match] [Query Top] |
| Summary | Alignment |
| | Name | JOLMA2013\_HNF1A\_full | | Database | JASPAR\_JOLMA.meme | | *p*-value | 4.59316e-07 | | *E*-value | 0.000431757 | | *q*-value | 0.000167563 | | Overlap | 14 | | Offset | 0 | | Orientation | Normal | |  |
| Create custom LOGO ↧ | [Previous Match] [Next Match] [Query Top] |
| Summary | Alignment |
| | Name | JASPAR2014\_HNF1B | | Database | JASPAR\_JOLMA.meme | | *p*-value | 9.91793e-07 | | *E*-value | 0.000932285 | | *q*-value | 0.000258439 | | Overlap | 12 | | Offset | -2 | | Orientation | Normal | |  |
| Create custom LOGO ↧ | [Previous Match] [Query Top] |

|  |  |
| --- | --- |
| Matches to Query: M162 (O162) | Previous Next Top |

|  |  |
| --- | --- |
| Matches to Query: M164 (O164) | Previous Next Top |

| Summary | Alignment |
| | Name | JASPAR2014\_FOXF2 | | Database | JASPAR\_JOLMA.meme | | *p*-value | 9.25444e-05 | | *E*-value | 0.0869917 | | *q*-value | 0.172052 | | Overlap | 14 | | Offset | 0 | | Orientation | Reverse Complement | |  |
| Create custom LOGO ↧ | [Query Top] |

|  |  |
| --- | --- |
| Matches to Query: M165 (O165) | Previous Next Top |

|  |  |
| --- | --- |
| Matches to Query: M166 (O166) | Previous Next Top |

| Summary | Alignment |
| | Name | JASPAR2014\_MAFF | | Database | JASPAR\_JOLMA.meme | | *p*-value | 3.87233e-05 | | *E*-value | 0.0363999 | | *q*-value | 0.0353007 | | Overlap | 15 | | Offset | 1 | | Orientation | Reverse Complement | |  |
| Create custom LOGO ↧ | [Next Match] [Query Top] |
| Summary | Alignment |
| | Name | JASPAR2014\_FOS | | Database | JASPAR\_JOLMA.meme | | *p*-value | 6.38772e-05 | | *E*-value | 0.0600446 | | *q*-value | 0.0353007 | | Overlap | 9 | | Offset | -6 | | Orientation | Normal | |  |
| Create custom LOGO ↧ | [Previous Match] [Next Match] [Query Top] |
| Summary | Alignment |
| | Name | JASPAR2014\_NFE2::MAF | | Database | JASPAR\_JOLMA.meme | | *p*-value | 6.44885e-05 | | *E*-value | 0.0606192 | | *q*-value | 0.0353007 | | Overlap | 14 | | Offset | -1 | | Orientation | Reverse Complement | |  |
| Create custom LOGO ↧ | [Previous Match] [Next Match] [Query Top] |
| Summary | Alignment |
| | Name | JASPAR2014\_JUN::FOS | | Database | JASPAR\_JOLMA.meme | | *p*-value | 7.58622e-05 | | *E*-value | 0.0713105 | | *q*-value | 0.0353007 | | Overlap | 7 | | Offset | -8 | | Orientation | Reverse Complement | |  |
| Create custom LOGO ↧ | [Previous Match] [Query Top] |

|  |  |
| --- | --- |
| Matches to Query: M167 (O167) | Previous Next Top |

|  |  |
| --- | --- |
| Matches to Query: M168 (O168) | Previous Next Top |

| Summary | Alignment |
| | Name | JOLMA2013\_FOXC1\_DBD | | Database | JASPAR\_JOLMA.meme | | *p*-value | 2.75577e-06 | | *E*-value | 0.00259042 | | *q*-value | 0.00506949 | | Overlap | 13 | | Offset | 0 | | Orientation | Normal | |  |
| Create custom LOGO ↧ | [Next Match] [Query Top] |
| Summary | Alignment |
| | Name | JOLMA2013\_FOXL1\_full\_2 | | Database | JASPAR\_JOLMA.meme | | *p*-value | 5.33456e-05 | | *E*-value | 0.0501449 | | *q*-value | 0.0394501 | | Overlap | 13 | | Offset | -1 | | Orientation | Normal | |  |
| Create custom LOGO ↧ | [Previous Match] [Next Match] [Query Top] |
| Summary | Alignment |
| | Name | JOLMA2013\_ONECUT3\_DBD | | Database | JASPAR\_JOLMA.meme | | *p*-value | 6.66317e-05 | | *E*-value | 0.0626338 | | *q*-value | 0.0394501 | | Overlap | 14 | | Offset | -1 | | Orientation | Normal | |  |
| Create custom LOGO ↧ | [Previous Match] [Query Top] |

|  |  |
| --- | --- |
| Matches to Query: M169 (O169) | Previous Next Top |

|  |  |
| --- | --- |
| Matches to Query: M170 (O170) | Previous Next Top |

|  |  |
| --- | --- |
| Matches to Query: M171 (O171) | Previous Next Top |

|  |  |
| --- | --- |
| Matches to Query: M172 (O172) | Previous Next Top |

|  |  |
| --- | --- |
| Matches to Query: M173 (O173) | Previous Next Top |

|  |  |
| --- | --- |
| Matches to Query: M174 (O174) | Previous Next Top |

|  |  |
| --- | --- |
| Matches to Query: M175 (O175) | Previous Next Top |

|  |  |
| --- | --- |
| Matches to Query: M176 (O176) | Previous Next Top |

|  |  |
| --- | --- |
| Matches to Query: M177 (O177) | Previous Next Top |

| Summary | Alignment |
| | Name | JASPAR2014\_KLF5 | | Database | JASPAR\_JOLMA.meme | | *p*-value | 1.09064e-05 | | *E*-value | 0.010252 | | *q*-value | 0.0203769 | | Overlap | 10 | | Offset | -2 | | Orientation | Normal | |  |
| Create custom LOGO ↧ | [Query Top] |

|  |  |
| --- | --- |
| Matches to Query: M178 (O178) | Previous Next Top |

|  |  |
| --- | --- |
| Matches to Query: M179 (O179) | Previous Next Top |

| Summary | Alignment |
| | Name | JOLMA2013\_FOXC1\_DBD | | Database | JASPAR\_JOLMA.meme | | *p*-value | 8.41086e-06 | | *E*-value | 0.00790621 | | *q*-value | 0.0155019 | | Overlap | 13 | | Offset | 0 | | Orientation | Normal | |  |
| Create custom LOGO ↧ | [Next Match] [Query Top] |
| Summary | Alignment |
| | Name | JOLMA2013\_ONECUT3\_DBD | | Database | JASPAR\_JOLMA.meme | | *p*-value | 8.10893e-05 | | *E*-value | 0.0762239 | | *q*-value | 0.0684166 | | Overlap | 14 | | Offset | -1 | | Orientation | Normal | |  |
| Create custom LOGO ↧ | [Previous Match] [Query Top] |

|  |  |
| --- | --- |
| Matches to Query: M180 (O180) | Previous Next Top |

| Summary | Alignment |
| | Name | JASPAR2014\_FOXP1 | | Database | JASPAR\_JOLMA.meme | | *p*-value | 2.78882e-05 | | *E*-value | 0.0262149 | | *q*-value | 0.0524299 | | Overlap | 15 | | Offset | -1 | | Orientation | Reverse Complement | |  |
| Create custom LOGO ↧ | [Query Top] |

|  |  |
| --- | --- |
| Matches to Query: M181 (O181) | Previous Next Top |

|  |  |
| --- | --- |
| Matches to Query: M182 (O182) | Previous Next Top |

|  |  |
| --- | --- |
| Matches to Query: M183 (O183) | Previous Next Top |

|  |  |
| --- | --- |
| Matches to Query: M184 (O184) | Previous Next Top |

| Summary | Alignment |
| | Name | JOLMA2013\_TFAP4\_full | | Database | JASPAR\_JOLMA.meme | | *p*-value | 8.03361e-05 | | *E*-value | 0.075516 | | *q*-value | 0.0928548 | | Overlap | 10 | | Offset | -2 | | Orientation | Reverse Complement | |  |
| Create custom LOGO ↧ | [Query Top] |

|  |  |
| --- | --- |
| Matches to Query: M185 (O185) | Previous Next Top |

| Summary | Alignment |
| | Name | JASPAR2014\_KLF5 | | Database | JASPAR\_JOLMA.meme | | *p*-value | 1.47392e-07 | | *E*-value | 0.000138548 | | *q*-value | 0.000276563 | | Overlap | 10 | | Offset | -4 | | Orientation | Normal | |  |
| Create custom LOGO ↧ | [Next Match] [Query Top] |
| Summary | Alignment |
| | Name | JASPAR2014\_SP1 | | Database | JASPAR\_JOLMA.meme | | *p*-value | 6.33658e-06 | | *E*-value | 0.00595638 | | *q*-value | 0.00594492 | | Overlap | 11 | | Offset | -4 | | Orientation | Normal | |  |
| Create custom LOGO ↧ | [Previous Match] [Next Match] [Query Top] |
| Summary | Alignment |
| | Name | JASPAR2014\_Klf4 | | Database | JASPAR\_JOLMA.meme | | *p*-value | 1.52658e-05 | | *E*-value | 0.0143499 | | *q*-value | 0.00954818 | | Overlap | 10 | | Offset | -4 | | Orientation | Reverse Complement | |  |
| Create custom LOGO ↧ | [Previous Match] [Next Match] [Query Top] |
| Summary | Alignment |
| | Name | JASPAR2014\_SP2 | | Database | JASPAR\_JOLMA.meme | | *p*-value | 6.35065e-05 | | *E*-value | 0.0596961 | | *q*-value | 0.0262582 | | Overlap | 14 | | Offset | 1 | | Orientation | Normal | |  |
| Create custom LOGO ↧ | [Previous Match] [Next Match] [Query Top] |
| Summary | Alignment |
| | Name | JASPAR2014\_EGR1 | | Database | JASPAR\_JOLMA.meme | | *p*-value | 6.99703e-05 | | *E*-value | 0.065772 | | *q*-value | 0.0262582 | | Overlap | 12 | | Offset | 2 | | Orientation | Normal | |  |
| Create custom LOGO ↧ | [Previous Match] [Query Top] |

|  |  |
| --- | --- |
| Matches to Query: M186 (O186) | Previous Next Top |

|  |  |
| --- | --- |
| Matches to Query: M187 (O187) | Previous Next Top |

|  |  |
| --- | --- |
| Matches to Query: M188 (O188) | Previous Next Top |

|  |  |
| --- | --- |
| Matches to Query: M189 (O189) | Previous Next Top |

| Summary | Alignment |
| | Name | JASPAR2014\_EGR1 | | Database | JASPAR\_JOLMA.meme | | *p*-value | 4.36128e-06 | | *E*-value | 0.0040996 | | *q*-value | 0.0081922 | | Overlap | 14 | | Offset | -3 | | Orientation | Reverse Complement | |  |
| Create custom LOGO ↧ | [Query Top] |

|  |  |
| --- | --- |
| Matches to Query: M190 (O190) | Previous Next Top |

|  |  |
| --- | --- |
| Matches to Query: M191 (O191) | Previous Next Top |

|  |  |
| --- | --- |
| Matches to Query: M193 (O193) | Previous Next Top |

|  |  |
| --- | --- |
| Matches to Query: M194 (O194) | Previous Next Top |

|  |  |
| --- | --- |
| Matches to Query: M195 (O195) | Previous Next Top |

|  |  |
| --- | --- |
| Matches to Query: M196 (O196) | Previous Next Top |

|  |  |
| --- | --- |
| Matches to Query: M197 (O197) | Previous Next Top |

|  |  |
| --- | --- |
| Matches to Query: M200 (O200) | Previous Next Top |

|  |  |
| --- | --- |
| Matches to Query: M201 (O201) | Previous Next Top |

| Summary | Alignment |
| | Name | JASPAR2014\_EGR1 | | Database | JASPAR\_JOLMA.meme | | *p*-value | 9.29387e-07 | | *E*-value | 0.000873623 | | *q*-value | 0.00149837 | | Overlap | 14 | | Offset | -4 | | Orientation | Normal | |  |
| Create custom LOGO ↧ | [Next Match] [Query Top] |
| Summary | Alignment |
| | Name | JASPAR2014\_SP2 | | Database | JASPAR\_JOLMA.meme | | *p*-value | 2.19971e-06 | | *E*-value | 0.00206773 | | *q*-value | 0.00149837 | | Overlap | 15 | | Offset | -3 | | Orientation | Normal | |  |
| Create custom LOGO ↧ | [Previous Match] [Next Match] [Query Top] |
| Summary | Alignment |
| | Name | JASPAR2014\_SP1 | | Database | JASPAR\_JOLMA.meme | | *p*-value | 2.43178e-06 | | *E*-value | 0.00228588 | | *q*-value | 0.00149837 | | Overlap | 11 | | Offset | -3 | | Orientation | Normal | |  |
| Create custom LOGO ↧ | [Previous Match] [Next Match] [Query Top] |
| Summary | Alignment |
| | Name | JOLMA2013\_KLF16\_DBD | | Database | JASPAR\_JOLMA.meme | | *p*-value | 4.32675e-05 | | *E*-value | 0.0406714 | | *q*-value | 0.0199948 | | Overlap | 11 | | Offset | -3 | | Orientation | Normal | |  |
| Create custom LOGO ↧ | [Previous Match] [Query Top] |

|  |  |
| --- | --- |
| Matches to Query: M202 (O202) | Previous Next Top |

|  |  |
| --- | --- |
| Matches to Query: M204 (O204) | Previous Next Top |

|  |  |
| --- | --- |
| Matches to Query: M205 (O205) | Previous Next Top |

|  |  |
| --- | --- |
| Matches to Query: M206 (O206) | Previous Next Top |

|  |  |
| --- | --- |
| Matches to Query: M207 (O207) | Previous Next Top |

|  |  |
| --- | --- |
| Matches to Query: M208 (O208) | Previous Next Top |

|  |  |
| --- | --- |
| Matches to Query: M212 (O212) | Previous Next Top |

|  |  |
| --- | --- |
| Matches to Query: M213 (O213) | Previous Next Top |

|  |  |
| --- | --- |
| Matches to Query: M217 (O217) | Previous Next Top |

|  |  |
| --- | --- |
| Matches to Query: M218 (O218) | Previous Next Top |

| Summary | Alignment |
| | Name | JASPAR2014\_SP1 | | Database | JASPAR\_JOLMA.meme | | *p*-value | 8.10321e-06 | | *E*-value | 0.00761702 | | *q*-value | 0.00855888 | | Overlap | 11 | | Offset | -3 | | Orientation | Normal | |  |
| Create custom LOGO ↧ | [Next Match] [Query Top] |
| Summary | Alignment |
| | Name | JASPAR2014\_KLF5 | | Database | JASPAR\_JOLMA.meme | | *p*-value | 9.16201e-06 | | *E*-value | 0.00861229 | | *q*-value | 0.00855888 | | Overlap | 10 | | Offset | -3 | | Orientation | Normal | |  |
| Create custom LOGO ↧ | [Previous Match] [Next Match] [Query Top] |
| Summary | Alignment |
| | Name | JOLMA2013\_SP1\_DBD | | Database | JASPAR\_JOLMA.meme | | *p*-value | 5.395e-05 | | *E*-value | 0.050713 | | *q*-value | 0.0271333 | | Overlap | 11 | | Offset | -3 | | Orientation | Normal | |  |
| Create custom LOGO ↧ | [Previous Match] [Next Match] [Query Top] |
| Summary | Alignment |
| | Name | JASPAR2014\_Zfx | | Database | JASPAR\_JOLMA.meme | | *p*-value | 5.80906e-05 | | *E*-value | 0.0546052 | | *q*-value | 0.0271333 | | Overlap | 14 | | Offset | 0 | | Orientation | Reverse Complement | |  |
| Create custom LOGO ↧ | [Previous Match] [Next Match] [Query Top] |
| Summary | Alignment |
| | Name | JOLMA2013\_KLF16\_DBD | | Database | JASPAR\_JOLMA.meme | | *p*-value | 8.46578e-05 | | *E*-value | 0.0795784 | | *q*-value | 0.0275394 | | Overlap | 11 | | Offset | -3 | | Orientation | Normal | |  |
| Create custom LOGO ↧ | [Previous Match] [Next Match] [Query Top] |
| Summary | Alignment |
| | Name | JASPAR2014\_EGR1 | | Database | JASPAR\_JOLMA.meme | | *p*-value | 8.84402e-05 | | *E*-value | 0.0831338 | | *q*-value | 0.0275394 | | Overlap | 14 | | Offset | -3 | | Orientation | Normal | |  |
| Create custom LOGO ↧ | [Previous Match] [Query Top] |

|  |  |
| --- | --- |
| Matches to Query: M219 (O219) | Previous Next Top |

|  |  |
| --- | --- |
| Matches to Query: M220 (O220) | Previous Next Top |

|  |  |
| --- | --- |
| Matches to Query: M221 (O221) | Previous Next Top |

|  |  |
| --- | --- |
| Matches to Query: M222 (O222) | Previous Next Top |

|  |  |
| --- | --- |
| Matches to Query: M225 (O225) | Previous Next Top |

| Summary | Alignment |
| | Name | JOLMA2013\_LMX1A\_DBD | | Database | JASPAR\_JOLMA.meme | | *p*-value | 6.79582e-05 | | *E*-value | 0.0638807 | | *q*-value | 0.0424401 | | Overlap | 8 | | Offset | -2 | | Orientation | Normal | |  |
| Create custom LOGO ↧ | [Next Match] [Query Top] |
| Summary | Alignment |
| | Name | JOLMA2013\_LMX1B\_DBD | | Database | JASPAR\_JOLMA.meme | | *p*-value | 9.47949e-05 | | *E*-value | 0.0891072 | | *q*-value | 0.0424401 | | Overlap | 8 | | Offset | -2 | | Orientation | Normal | |  |
| Create custom LOGO ↧ | [Previous Match] [Query Top] |

|  |  |
| --- | --- |
| Matches to Query: M226 (O226) | Previous Next Top |

| Summary | Alignment |
| | Name | JASPAR2014\_ZNF263 | | Database | JASPAR\_JOLMA.meme | | *p*-value | 6.37098e-09 | | *E*-value | 5.98872e-06 | | *q*-value | 1.19774e-05 | | Overlap | 20 | | Offset | 1 | | Orientation | Reverse Complement | |  |
| Create custom LOGO ↧ | [Query Top] |

|  |  |
| --- | --- |
| Matches to Query: M228 (O228) | Previous Next Top |

|  |  |
| --- | --- |
| Matches to Query: M229 (O229) | Previous Next Top |

|  |  |
| --- | --- |
| Matches to Query: M230 (O230) | Previous Next Top |

| Summary | Alignment |
| | Name | JASPAR2014\_EGR1 | | Database | JASPAR\_JOLMA.meme | | *p*-value | 1.70033e-05 | | *E*-value | 0.0159831 | | *q*-value | 0.0316654 | | Overlap | 14 | | Offset | 0 | | Orientation | Normal | |  |
| Create custom LOGO ↧ | [Query Top] |

|  |  |
| --- | --- |
| Matches to Query: M231 (O231) | Previous Next Top |

|  |  |
| --- | --- |
| Matches to Query: M232 (O232) | Previous Next Top |

| Summary | Alignment |
| | Name | JASPAR2014\_EBF1 | | Database | JASPAR\_JOLMA.meme | | *p*-value | 2.00084e-05 | | *E*-value | 0.0188079 | | *q*-value | 0.0376158 | | Overlap | 11 | | Offset | -5 | | Orientation | Normal | |  |
| Create custom LOGO ↧ | [Query Top] |

|  |  |
| --- | --- |
| Matches to Query: M233 (O233) | Previous Next Top |

| Summary | Alignment |
| | Name | JASPAR2014\_ZNF263 | | Database | JASPAR\_JOLMA.meme | | *p*-value | 2.8748e-06 | | *E*-value | 0.00270231 | | *q*-value | 0.00540463 | | Overlap | 20 | | Offset | 1 | | Orientation | Normal | |  |
| Create custom LOGO ↧ | [Next Match] [Query Top] |
| Summary | Alignment |
| | Name | JASPAR2014\_EWSR1-FLI1 | | Database | JASPAR\_JOLMA.meme | | *p*-value | 8.95644e-05 | | *E*-value | 0.0841906 | | *q*-value | 0.0841906 | | Overlap | 18 | | Offset | -2 | | Orientation | Normal | |  |
| Create custom LOGO ↧ | [Previous Match] [Query Top] |

|  |  |
| --- | --- |
| Matches to Query: M234 (O234) | Previous Next Top |

|  |  |
| --- | --- |
| Matches to Query: M235 (O235) | Previous Next Top |

| Summary | Alignment |
| | Name | JASPAR2014\_TFAP2C | | Database | JASPAR\_JOLMA.meme | | *p*-value | 4.84946e-07 | | *E*-value | 0.000455849 | | *q*-value | 0.00088718 | | Overlap | 15 | | Offset | -1 | | Orientation | Normal | |  |
| Create custom LOGO ↧ | [Next Match] [Query Top] |
| Summary | Alignment |
| | Name | JOLMA2013\_Tcfap2a\_DBD\_3 | | Database | JASPAR\_JOLMA.meme | | *p*-value | 3.54817e-05 | | *E*-value | 0.0333528 | | *q*-value | 0.0254699 | | Overlap | 13 | | Offset | -3 | | Orientation | Reverse Complement | |  |
| Create custom LOGO ↧ | [Previous Match] [Next Match] [Query Top] |
| Summary | Alignment |
| | Name | JOLMA2013\_TFAP2C\_full\_2 | | Database | JASPAR\_JOLMA.meme | | *p*-value | 5.41731e-05 | | *E*-value | 0.0509227 | | *q*-value | 0.0254699 | | Overlap | 13 | | Offset | -3 | | Orientation | Reverse Complement | |  |
| Create custom LOGO ↧ | [Previous Match] [Next Match] [Query Top] |
| Summary | Alignment |
| | Name | JASPAR2014\_TFAP2A | | Database | JASPAR\_JOLMA.meme | | *p*-value | 6.6273e-05 | | *E*-value | 0.0622966 | | *q*-value | 0.0254699 | | Overlap | 15 | | Offset | -1 | | Orientation | Normal | |  |
| Create custom LOGO ↧ | [Previous Match] [Next Match] [Query Top] |
| Summary | Alignment |
| | Name | JOLMA2013\_TFAP2C\_DBD\_3 | | Database | JASPAR\_JOLMA.meme | | *p*-value | 6.96111e-05 | | *E*-value | 0.0654344 | | *q*-value | 0.0254699 | | Overlap | 13 | | Offset | -3 | | Orientation | Reverse Complement | |  |
| Create custom LOGO ↧ | [Previous Match] [Query Top] |

|  |  |
| --- | --- |
| Matches to Query: M238 (O238) | Previous Next Top |

Previous Top

##### TOMTOM version

4.10.0 (Release date: Wed May 21 10:35:36 2014 +1000)

##### Reference

Shobhit Gupta, JA Stamatoyannopolous, Timothy Bailey and William Stafford Noble,
"Quantifying similarity between motifs",
*Genome Biology*, **8**(2):R24, 2007.

##### Command line summary

tomtom -thresh 0.1 -evalue -oc HuPanIs\_StretchEnhancersDHS\_RemovedRepeats\_JJ\_TOMTOM HuPanIs\_StretchEnhancersDHS\_RemovedRepeats.meme /home/quangdx/meme/db/motif\_databases/JASPAR\_JOLMA.meme  
Background letter frequencies (from first motif database):  

A: 0.250   C: 0.250   G: 0.250   T: 0.250

  
Result calculation took 3326.145 seconds

show model parameters...

##### Model parameters

distance\_measure = value: "pearson"
threshold = type: "evalue"
background = from: "first\_target", A: "0.25", C: "0.25", G: "0.25", T: "0.25"
host = kr03n20.gc.nih.gov
when = Wed Aug 6 10:26:26 2014

hide model parameters...

  
  
  
  
  
  
  
  
  
  
  
  
  
  
  
  
  
  
  
  
  
  
  
  
  
  
  
  
  
  
  
  
  
  
  
  
  
  
  
  
  
  
  
  
  
  
  
  
  
  
  
  
  
